# Transcriptome Sequencing of CeRNA Network Constructing in Status Epilepticus Mice Treated by Low-Frequency Repetitive Transcranial Magnetic Stimulation

**DOI:** 10.1007/s12031-023-02108-z

**Published:** 2023-05-03

**Authors:** Shaotian Zhang, Huihui Zou, Xiaopei Zou, Jiaqia Ke, Bofang Zheng, Xinrun Chen, Xianju Zhou, Jiana Wei

**Affiliations:** 1 Department of Neurology, Second Affiliated Hospital, Guangzhou Medical University, No.250 East Changgang Rd, Guangzhou, 510260 China; 2 Special Medical Service Center, Neuroscience Center, Integrated Hospital of Traditional Chinese Medicine, Southern Medical University, No.13 Shi Liu Gang Rd, Haizhu District, Guangzhou, Guangdong China; 3 Department of Neurology, Neuroscience Center, Integrated Hospital of Traditional Chinese Medicine, Southern Medical University, No.13 Shi Liu Gang Rd, Haizhu District, Guangzhou, Guangdong 510315 China; 4 Department of Clinical Medicine, The First Clinical College of Guangzhou Medical University, Guangzhou, Guangdong 510315 China

**Keywords:** Status epilepticus, Transcriptome sequencing, Low-frequency rTMS, CeRNAs, LncRNA, MiRNA, Interaction network

## Abstract

It is shown that great progress was recently made in the treatment of repetitive transcranial magnetic stimulation (rTMS) for neurological and psychiatric diseases. This study aimed to address how rTMS exerted it therapeutic effects by regulating competitive endogenous RNAs (ceRNAs) of lncRNA-miRNA-mRNA. The distinction of lncRNA, miRNA and mRNA expression in male status epilepticus (SE) mice treated by two different ways, low-frequency rTMS (LF-rTMS) vs. sham rTMS, was analyzed by high-throughput sequencing. The Gene Ontology (GO) functional enrichment and Kyoto Encyclopedia of Genes and Genomes (KEGG) pathway enrichment analyses were carried out. Gene–Gene Cross Linkage Network was established; pivotal genes were screened out. qRT-PCR was used to verify gene–gene interactions. Our results showed that there were 1615 lncRNAs, 510 mRNAs, and 17 miRNAs differentially which were expressed between the LF-rTMS group and the sham rTMS group. The expression difference of these lncRNAs, mRNAs, and miRNAs by microarray detection were consistent with the results by qPCR. GO functional enrichment showed that immune-associated molecular mechanisms, biological processes, and GABA-A receptor activity played a role in SE mice treated with LF-rTMS. KEGG pathway enrichment analysis revealed that differentially expressed genes were correlated to T cell receptor signaling pathway, primary immune deficiency and Th17 cell differentiation signaling pathway. Gene–gene cross linkage network was established on the basis of Pearson’s correlation coefficient and miRNA. In conclusion, LF-rTMS alleviates SE through regulating the GABA-A receptor activity transmission, improving immune functions, and biological processes, suggesting the underlying ceRNA molecular mechanisms of LF-rTMS treatment for epilepsy.

**Supplementary Information:**

The online version contains supplementary material available at 10.1007/s12031-023-02108-z.

## Introduction

Epilepsy, not rare as a neurological disease, has a negative impact on sufferers’ lives (Devinsky et al. 2018). Epilepsy, with a character of unexplained spontaneous episodes on a regular basis, is considered to be an important cause of death and disability. Much progress has been made in the treatment of epilepsy over the past 20 years, but a significant challenge still exists. The current treatment of epilepsy focuses on preventing or inhibiting the symptoms of epilepsy, far from delaying the progression of the disease. Therefore, exploring new therapies is of great significance for the recovery of epileptic patients.

Since the establishment of non-invasive transcranial magnetic stimulation (TMS) in the 1980s (Barker et al. 1986, 1985). Repetitive TMS (rTMS) protocols are applicable for stimulating the human brain, including patterned and straightforward mode (e.g., theta burst stimulation) that make it no longer difficult to examine or change brain plasticity in disease-free volunteers and patients with neurological disorders. rTMS is one type of TMS that can be applied widely in the treatment of various neuropsychiatric diseases, such as Alzheimer’s disease, epilepsy, depression and anxiety disorder, sleep disorder, and neurodevelopmental disorders (Lefaucheur et al. 2020; Somaa et al. 2022).

The basic fundamental of TMS is to provoke magnetic fields above the brain to activate cortical neurons indirectly through synaptic inputs (Driver et al. 2009; Ruff et al. 2006). It was reported that rTMS can activate cortical areas far from the stimulation center and generate long-term electrophysiological changes (Rehn et al. 2018). Thus TMS can transmit EEG signals to the distal end of the stimulus center via synaptic connections (Post and Keck 2001). There is evidence that high-frequency (HF) rTMS (>5 Hz) evokes an excitatory effect, while low-frequency (LF) rTMS (<1 Hz) leads to an inhibitory effect in the brain (Fitzgerald et al. 2006). LF-rTMS is still a promising treatment approach for epilepsy (Shon et al. 2019; Zhang et al. 2019; Li et al. 2019a) although there is no strong clinical evidence to support the efficacy of LF-rTMS for epilepsy (Walton et al. 2021). Long-time depression (LTD) is considered to be one of the key mechanisms for LF-rTMS to alter cortico-motor excitability; the molecular mechanisms underlying LTD may involve NMDA receptors on the postsynaptic membrane (Chervyakov et al. 2015). Other studies suggest that LF-rTMS can lower the depolarization response of mouse neurons to achieve the purpose of anticonvulsant (Chameh et al. 2015; Shojaei et al. 2014). Furthermore, LF-rTMS caused the release of some anticonvulsant substances to cerebrospinal fluid and as a result produced antiepileptic effect (Anschel et al. 2003). rTMS improved the release of neurotransmitters, such as 5-HT, glutamate, and gamma-aminobutyric acid (GABA), in the specific brain regions of animals (Keck et al. 2000) and depressed patients (Baeken et al. 2011).

Recently, many studies have demonstrated that differentially expressed lncRNAs (DELs) and differentially expressed miRNA (DEMs) play a necessary role in normal physiological functions by modulating gene expression at epigenetic, transcription and post-transcription level (Beermann et al. 2016). Furthermore, increasing evidence suggests that DELs and DEMs are emerging in the pathogenesis, diagnosis, and treatment of epilepsy (Ghafouri-Fard et al. 2022; Henshall et al. 2016). Unlike protein-coding genes, lncRNAs have significant advances as diagnostic and prognostic biomarkers. It is currently known that lncRNA is involved in nervous system development, synapse occurrence, and synaptic plasticity. The disorder of lncRNA may cause neurological diseases such as epilepsy, Parkinson’s disease (PD), and Alzheimer’s disease (AD) (Yamada 2019). LncRNA and miRNA are probably related to specific biological processes in epilepsy, including ion/gated channels and GABA receptors (Gross and Tiwari 2018; Haenisch et al. 2016). LncRNA might also be involved in neuronal apoptosis, neuroinflammation, and cognitive impairment in the development of epilepsy (Villa et al. 2019). Specifically, miR-211 and miR-128 are essential factors that directly induce epilepsy in mice (Fan et al. 2016; Tan et al. 2013). Therefore, ceRNAs are expected to exert their roles in the treatment of epilepsy.

As mentioned above, LF-rTMS is a potential therapeutic strategy for epilepsy, but until now there is no evidence about the ceRNA molecular mechanisms of LF-rTMS treatment for epilepsy. In the present study, we established a gene–gene cross linkage network based on LF-rTMS related competitive endogenous RNAs (ceRNAs), indicating a new direction for exploring the intrinsic mechanisms of LF-rTMS treatment.

## Materials and Methods

### Mouse Models of Status Epilepticus

Adult male C57BL/6 mice (22–28 g) purchased from Changzhou Cavens Laboratory Animal Co., Ltd. were raised at 25 °C, 60% humidity, 12 h:12 h light–dark cycle. Sufficient water and food was provided. Establishment of status epilepticus (SE) model was performed by intraperitoneal injection of pilocarpine. Specifically, mice were weighed and given intraperitoneal injection of pilocarpine (300 mg/kg, Sigma, St. Louis, MO, USA), 30 min before intraperitoneal injection of methyl scopolamine (2 ml/kg, Sigma, St. Louis, MO, USA) to block the peripheral cholinergic side effects of pilocarpine. The seizures of mice were observed, and the severity of seizures was ranked by the modified Racine score (Racine 1972). The SE mice were considered for this study. Intraperitoneally injection of 4 mg/kg diazepam (Sigma, St. Louis, MO, USA) to quell seizures was performed after SE onset for 2 h. LF-rTMS was delivered with the following parameters: 0.5 Hz, 600 pulses, 20 min, 20% intensity, 5 consecutive days (one in the morning and one in the afternoon). At 6 h after magnetic stimulation of different time points, the mice were killed, and the cortex were collected. Animal experiments complied with the Institutional Animal Care and Use Committee (IACUC) of Southern Medical University.

### Procedure for Microelectrode Implantation, Recording, and Analysis of Video-EEG Recording

After rTMS, mice were implanted with stainless steel microelectrodes. In brief, mice in each group were anesthetized by inhalation of 2% isoflurane and then fixed on the brain stereotaxic apparatus. Electrodes were implanted in bilateral somatosensory cortex (Bregma, −1.0 mm; lateral, 2.0 mm; depth, 1.8 mm) and reference electrodes were implanted in shallow frontal cortex (Bregma, + 2.6 mm; lateral, 1.8 mm; depth, 0.5 mm). EEG was recorded 5 days after surgery. Acquisition system included 8 single-channel biological amplifiers (AD Instruments Ltd., Sydney, Australia) and analog–digital converter (PowerLab ML870, AD Instruments), acquisition conditions: sampling rate 200 Hz, time constant 0.1 s, low-pass filter 60 by using the software Lab chart V6.0 or higher (AD Instruments). Simultaneous digital vEEG recording was conducted using high-resolution infrared cameras. Abnormal EEG (most likely paroxysmal) was analyzed to monitor epileptic seizures in mice. The epileptiform spikes were different from artifacts according to amplitude and duration of spikes and interspike interval. All epileptic spikes above baseline form a spike wave group. The times of spontaneous recurrent seizure (SRS) incidence and the spike rate were quantified using NeuroScore 3.2.0 software.

### Experimental Procedures

Mice were distributed into two groups: (a) the SE group (*n* = 12) with the sham stimulation (the coil was far from the surface of the brain, thus failing to produce an effective magnetic stimulation) and (b) the SE group with rTMS (*n* = 14). Wild-type C57BL/6 mice were placed into a restraint device for rTMS (Zhang et al. 2019). On the animal head center, a 70-mm figure-of-eight coil (YIRUIDE, Wuhan, China, 3.0 Tesla) was placed. LF-rTMS was delivered with the following parameters: 0.5 Hz, 600 pulses, 20 min, 20% intensity (triggering no obvious muscle twitches of limbs), and 5 days (once each morning and afternoon). For the sake of adapting to the restraint device, naive mice were put into it three times (lasting for 1 to 2 min every time) a day before the first rTMS. Six hours after the whole rTMS (day 5), cervical dislocation was adopted to sacrifice the mice. Then their cerebral cortex were taken out and kept at −80 °C before needed. The cortex from two different mice in each group was used for transcriptome sequencing of ceRNA network constructing.

### RNA Extraction and Microarray Experiments

The experiments carried out were in the lab of OeBiotech Corporation (Shanghai, China). Agilent Mouse lncRNA Microarray V3 (4*180 K, Design ID: 084,388) and miRNA Microarray Release 21.0 (8*60 K, Design ID: 070,155) were used in this experiment (Tang et al. 2019). In short, TRIzol method was employed to extract RNA. Then, it was quantified through NanoDrop ND-2000 (Thermo Scientific). Agilent Bio Analyzer 2100 (Agilent Technologies) was used to evaluate RNA integrity. The sample labeling, microarray hybridization, and washing were administered under manufacturer’s standard protocol. In summary, the RNA was amplified according to requirements of the manufacturer, then labeled with cyanine-3-CTP, purified, and hybridized to the chip. Furthermore, the arrays were finally scanned by the Agilent Scanner G2505C (Agilent Technologies) after washing.

### Construction of lncRNA-miRNA-mRNA

The ceRNA network was constructed by the differentially expressed lncRNAs (DELs), mRNAs (DEGs), and miRNAs (DEMs). Firstly, the forecasted targets of DEMs were from miRanda as well as Pearson correlation coefficient (PCC). miRanda (http://www.microrna.org/microrna/getDownloads.do) was employed to forecast the miRNA responsive elements (MRE) of target genes. Moreover, binding sites of assayed miRNAs on basis of the sequences of lncRNAs and mRNAs were also from it (Enright et al. 2003). Before the determination of overlap RNAs, these forecasted mRNAs and lncRNAs were in comparison with those assayed from microarray. The pseudo-positive of coupled miRNA-mRNA and miRNA-lncRNA was reduced after filtrating the highly coupled miRNA-mRNA and miRNA-lncRNA through the PCC and calculating the *P* value of PCC of every single couple through Fisher’s asymptotic distribution (Guttman et al. 2009). The interactions of negative PCC were supposed to be selected only when its *P* value < 0.01. Then shared couples from the forecasted coupled miRNA-mRNA as well as miRNA-lncRNA through miRanda and PCC were included for further analyses. Finally, a ceRNA network correlated with the SE mice treated with LF-rTMS was plotted by Cytoscape 3.7.2 (https://cytoscape.org/) in reference to forecasted shared couples of miRNA-mRNA as well as miRNA-lncRNA.

### Quantitative Reverse Transcription-Polymerase Chain Reaction Analysis

To confirm results of microarray, real-time reverse transcription polymerase chain reaction (RT-PCR) was adopted to detect the expression levels of cirRNAs, lncRNAs, and miRNAs and mRNAs in the cerebral cortex of C57BL/6 mice. According to the corresponding sequences in GenBank, primers were synthesized by Guangzhou Aiji Biotechnology Co. Ltd. RNA was extracted strictly according to the TRIzol method, and the obtained RNA A260/A280 > 1.8 met the purity requirement. The total RNA extracted was subjected to reverse transcription and PCR amplification according to the instructions of RT-PCR reaction kit (TaKaRa). PCR reaction conditions: 95 °C 30 s, 95 °C 5 s, 60 °C 20 s, a total of 40 cycles. The confirmation data is shown in Supplemental Fig. 1.

**Fig. 1:**
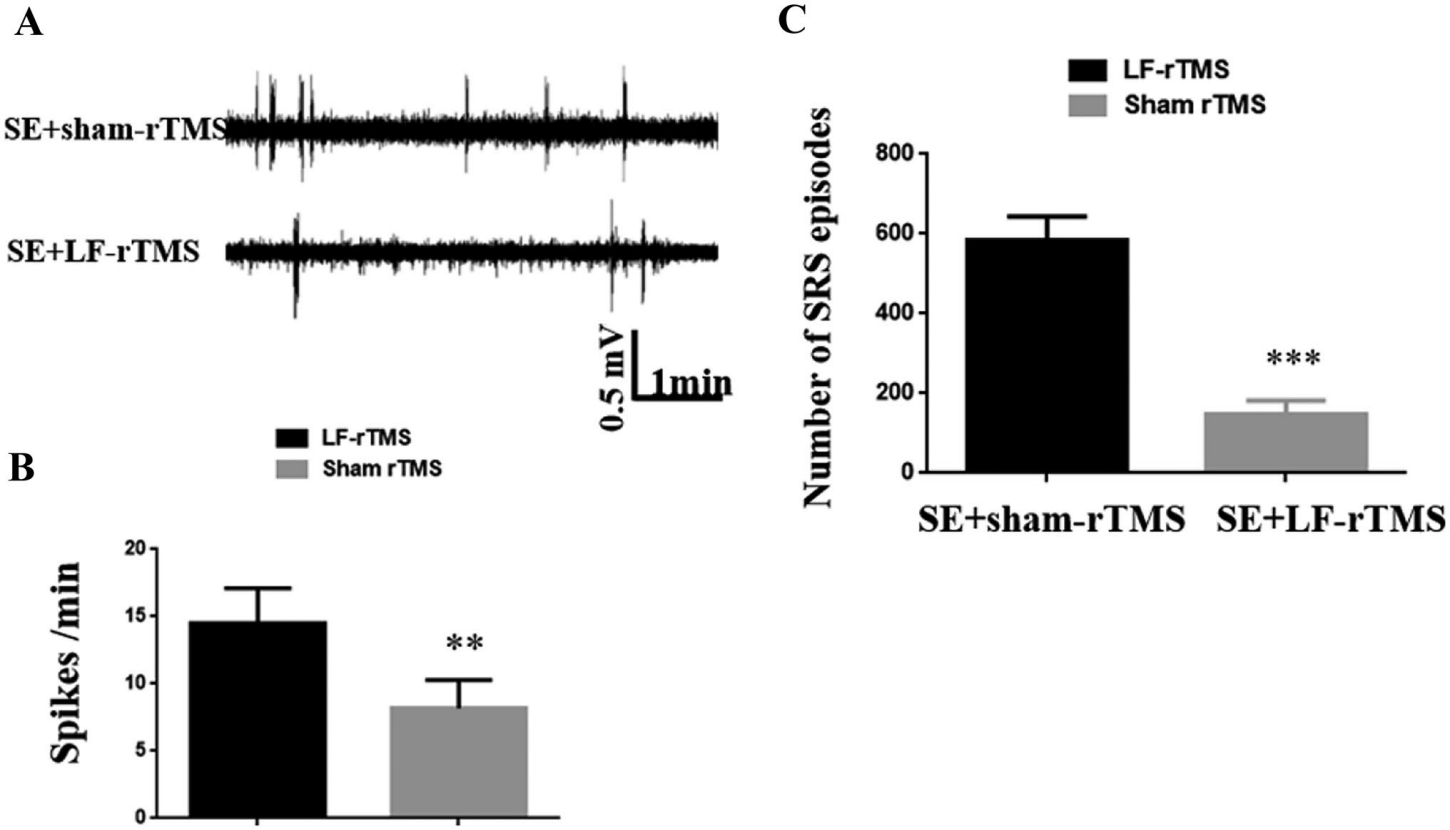
LF-rTMS significantly exerts an anti-epileptogenic effect during SE. **A** Representative EEG traces from the LF-rTMS group and the sham rTMS group; **B** the comparison between the numbers of SRS between the sham rTMS group(*n*=12) and the rTMS group (*n*=14); **C** the comparison of epileptiform frequencies between the two groups. **p < 0.01, ***p < 0.001, Student’s *t* test

### Data Analysis

The array images were examined by feature extraction software (version 10.7.1.1, Agilent Technologies) to elicit the original data. Next, the fundamental analysis of the original data was accomplished by Gene spring (version 13.1, Agilent Technologies). Primarily, the original data was standardized processing by the quantile algorithm. The lncRNA microarray probes t with fewest one conditions out of 2 conditions tagged in “P” were selected for further data analysis. The miRNA Microarray probes that at least 100.0 percent of samples in either condition have tagged in “Detected” were selected for next data analysis. Differential expression of lncRNAs (DELs), mRNAs (DEGs), and miRNAs (DEMs) were obtained by calculating fold change and *P* value obtained by *t* test. Ones with fold change ≥ 2.0 and *P* value < 0.05 were considered as differentially expressed genes. The same genes were selected from the differentially expressed target genes predicted by the three databases (TargetScan, microRNAorg, PITA). Afterward, GO analysis and KEGG analysis was utilized to expound the roles of these differentially expressed mRNAs and the target mRNAs of abnormal miRNAs. Finally, hierarchical clustering and volcano plots were performed to describe the distinct mRNA, lncRNA, or miRNA expression patterns schema among samples.

## Results

### LF-rTMS Significantly Decreases Epileptiform Discharges and Spontaneous Seizures During SE

To determine whether LF-rTMS exerted a significant anti-epileptogenic effect in SE mice, we applied video-EEG to monitor epileptiform discharges and seizure behaviors after magnetic stimulation. As shown in Fig. 1, in contrast to the sham rTMS group (*n*=12), the amount of epileptic spikes in LF-rTMS group (*n*=14) was decreased significantly (Fig. 1A–B). Correspondingly, the number of spontaneous recurrent seizures was also decreased in SE mice treated with LF-rTMS (Fig. 1C).

### Differentially Expressed RNAs in SE Mice Treated with Low-Frequency rTMS

**Fig. 2:**
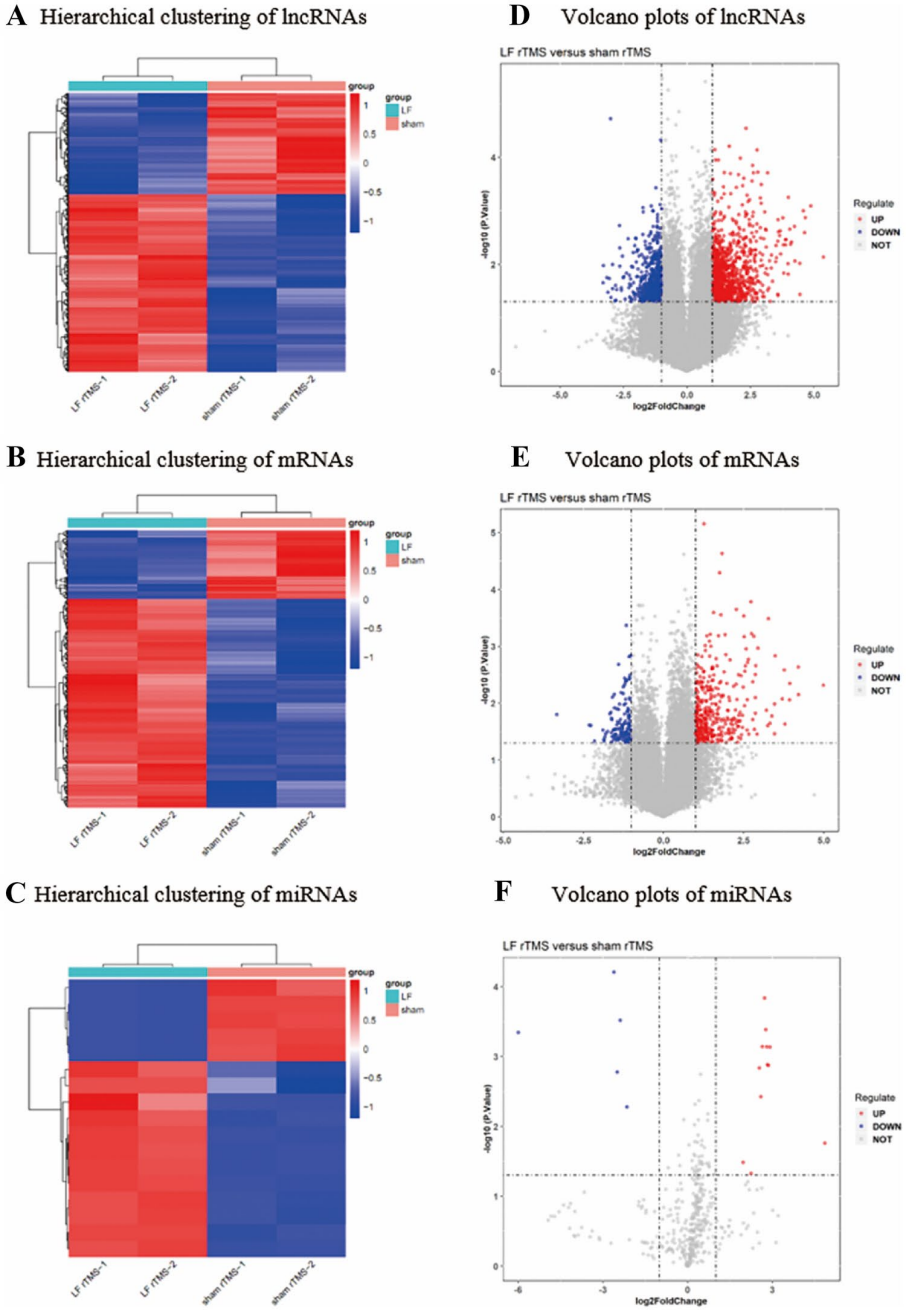
Hierarchical clustering and volcano plots of dysregulated miRNA, mRNA, and lncRNA. Hierarchical clustering shows all dysregulated lncRNAs (**A**), mRNAs (**C**), and miRNAs (**E**) in the LF-rTMS-treated mice as compared to the sham rTMS-treated mice. Red represents high relative expression, and blue represents low relative expression. Volcano plots were performed to assess the variation and reproducibility of lncRNAs (**B**), mRNAs (**D**), and miRNAs (**E**) expression between the LF-rTMS-treated mice as compared to the sham rTMS-treated mice. Red spots indicate upregulated genes, while blue spots represent downregulated genes

The standardized original data from array graph were utilized to assess the expression levels of lncRNAs, mRNAs, and miRNA in the sham rTMS-treated SE mice and the LF-rTMS-treated SE mice. A total of 1615 significantly dysregulated (1029 up- and 586 downregulated) lncRNAs were determined (Supplementary Table S1). A sum of 510 genes were recognized to be distinctly expressed in SE mice treated with LF-rTMS when compared to the sham rTMS-treated SE mice. These genes included 383 upregulated genes and 127 downregulated genes (Supplementary Table S2). There were 209 upregulated and 68 downregulated circRNAs (Supplementary Table S3). A total of 17 differentially expressed miRNAs were initially screened, including 12 upregulated and 5 downregulated miRNAs (Supplementary Table S4). The volcano and heat maps are shown in Figs. 1 and 2. The top upregulated and downregulated 10 significantly DELs, DEMs, and DEGs are listed in Table 1. The distinguishable lncRNA, mRNA, and miRNA expression patterns in the two groups were displayed by hierarchical clustering (Fig. 2). The differentiable expression patterns of lncRNA, mRNA, and miRNA in the two groups were rendered by accomplishing hierarchical clustering.

**Table 1:** Differentially expressed genes (DEGs) in SE mice treated with LF-rTMS as compared with SE mice treated with sham rTMS

**Regulate**	**lncRNA**			**mRNA**			**miRNA**		
	**TargetID**	**log**_**2**_**FC**	***P*.Value**	**GeneSymbol**	**log**_**2**_**FC**	***P*.Value**	**miRNA name**	**log**_**2**_**FC**	***P* value**
Up	NONMMUT135597.1	5.36851	0.00734	Lsmem1	4.98728	0.00487	mmu-miR-6984-3p	4.85315	0.01743
	XR_870399	4.87991	0.00080	Zfp235	4.21518	0.00713	mmu-miR-28a-5p	2.91005	0.00073
	NONMMUT113376.1	4.66178	0.00134	Isl1	4.21013	0.00233	mmu-miR-376a-5p	2.86186	0.00133
	NONMMUT136502.1	4.63616	0.00256	Asb3	3.94305	0.00451	mmu-miR-138–2-3p	2.82168	0.00131
	NONMMUT017997.2	4.57706	0.00099	Slc22a26	3.93373	0.00881	mmu-miR-7216-5p	2.79403	0.00073
	NONMMUT130005.1	4.45396	0.03657	Lpxn	3.79102	0.02338	mmu-miR-342-5p	2.76946	0.00041
	NONMMUT120599.1	4.44599	0.00958	Tcaf3	3.77073	0.00261	mmu-miR-186-5p	2.72357	0.00015
	NONMMUT083899.1	4.25848	0.00869	Armcx6	3.70858	0.00842	mmu-miR-379-3p	2.64449	0.00072
	NONMMUT115312.1	4.23432	0.00195	Rbm47	3.48942	0.00148	mmu-miR-3102-3p	2.59325	0.00378
	NONMMUT017995.2	4.11820	0.00383	Irf6	3.46616	0.03459	mmu-miR-29b-1-5p	2.53349	0.00146
Down	NONMMUT124392.1	−3.30967	0.02232	Gm904	−3.32593	0.01590	mmu-miR-7222-3p	−5.99159	0.00045
	NONMMUT085198.1	−3.28886	0.01050	Timm10b	−2.31445	0.02437	mmu-miR-6973b-5p	−2.60895	0.00006
	NONMMUT131055.1	−3.15322	0.01775	CA465148	−2.26467	0.02471	mmu-miR-1934-3p	−2.49315	0.00168
	NONMMUT120897.1	−3.11824	0.03348	Nuf2	−2.15046	0.04716	mmu-miR-19a-3p	−2.38574	0.00030
	NR_040578	−3.06152	0.01840	Olfr180	−1.89311	0.02974	mmu-miR-744-5p	−2.14357	0.00529
	NR_015609	−3.00397	0.00002	Novel	−1.85536	0.04408	NA	NA	NA
	XR_863618	−2.99008	0.04145	Cbln3	−1.84957	0.04584	NA	NA	NA
	TC1616118	−2.93429	0.01006	Podxl	−1.82505	0.03964	NA	NA	NA
	NONMMUT016592.2	−2.92660	0.02872	Kdm2a	−1.80582	0.03478	NA	NA	NA
	NONMMUT116230.1	−2.83651	0.04839	Olfr124	−1.74116	0.03158	NA	NA	NA

### The GO and the KEGG Pathway Enrichment Analysis

**Fig. 3:**
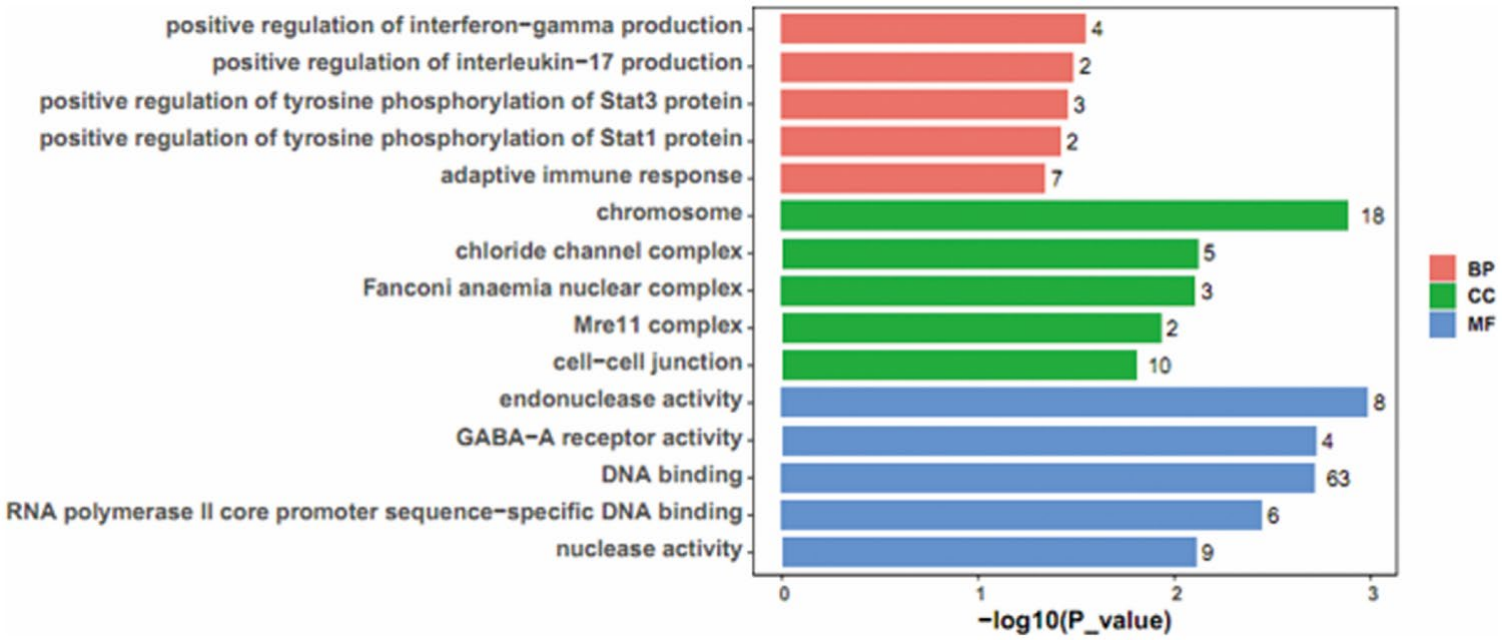
Top 5 GO pathway enrichment items of differentially expressed mRNAs between the LF-rTMS-treated mice as compared to the sham rTMS-treated mice. The GO enrichment items were sorted by significance separately in biological process, cellular component, and molecular function

**Fig. 4:**
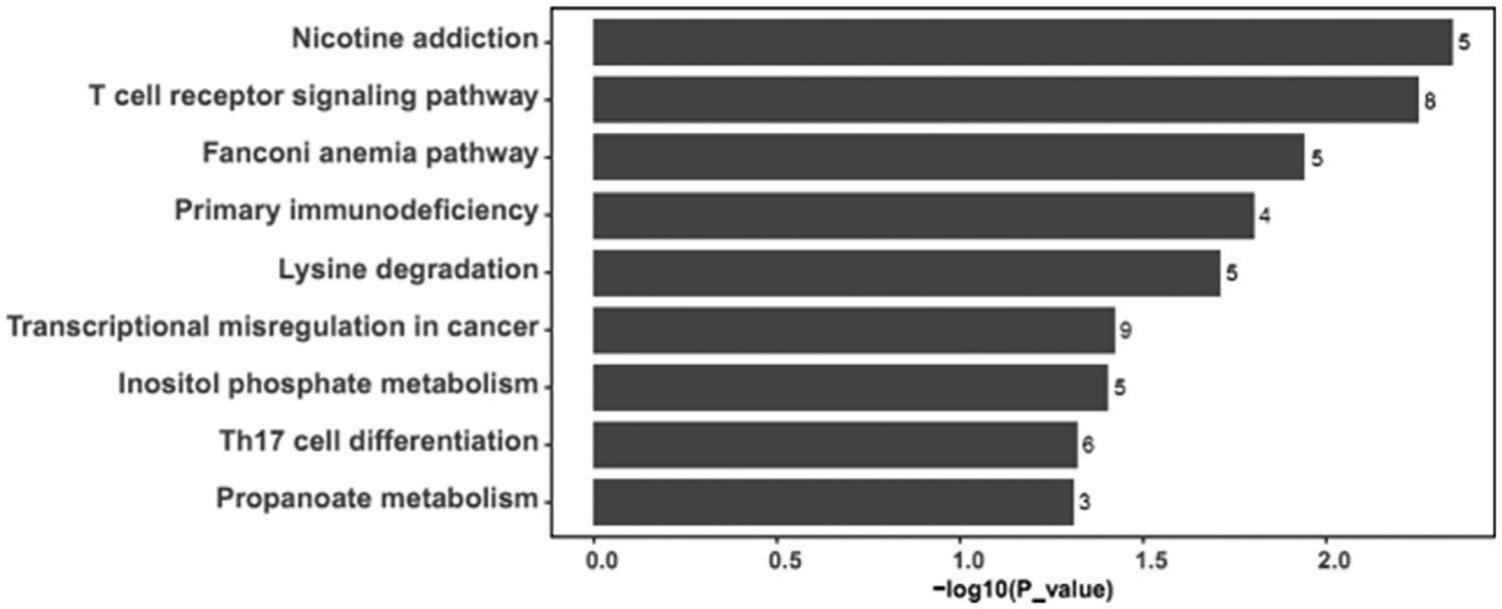
KEGG pathway enrichment of differentially expressed mRNAs

The GO and the KEGG pathway enrichment analyses were carried out on DEGs in the lncRNA-related ceRNA network. Using the Cluster Profile package in R software, GO analysis displayed DEGs were related to “GABA-A receptor activity” (Fig. 3). KEGG pathway analysis utilizing the Cluster Profiler package indicated DEGs had an obvious relevance with various immune-related pathways, including T cell receptor signaling pathway, primary immunodeficiency, and Th17 cell differentiation as well (Fig. 4).

### The ceRNA Network in SE Mice Treated with LF- rTMS

**Fig. 5:**
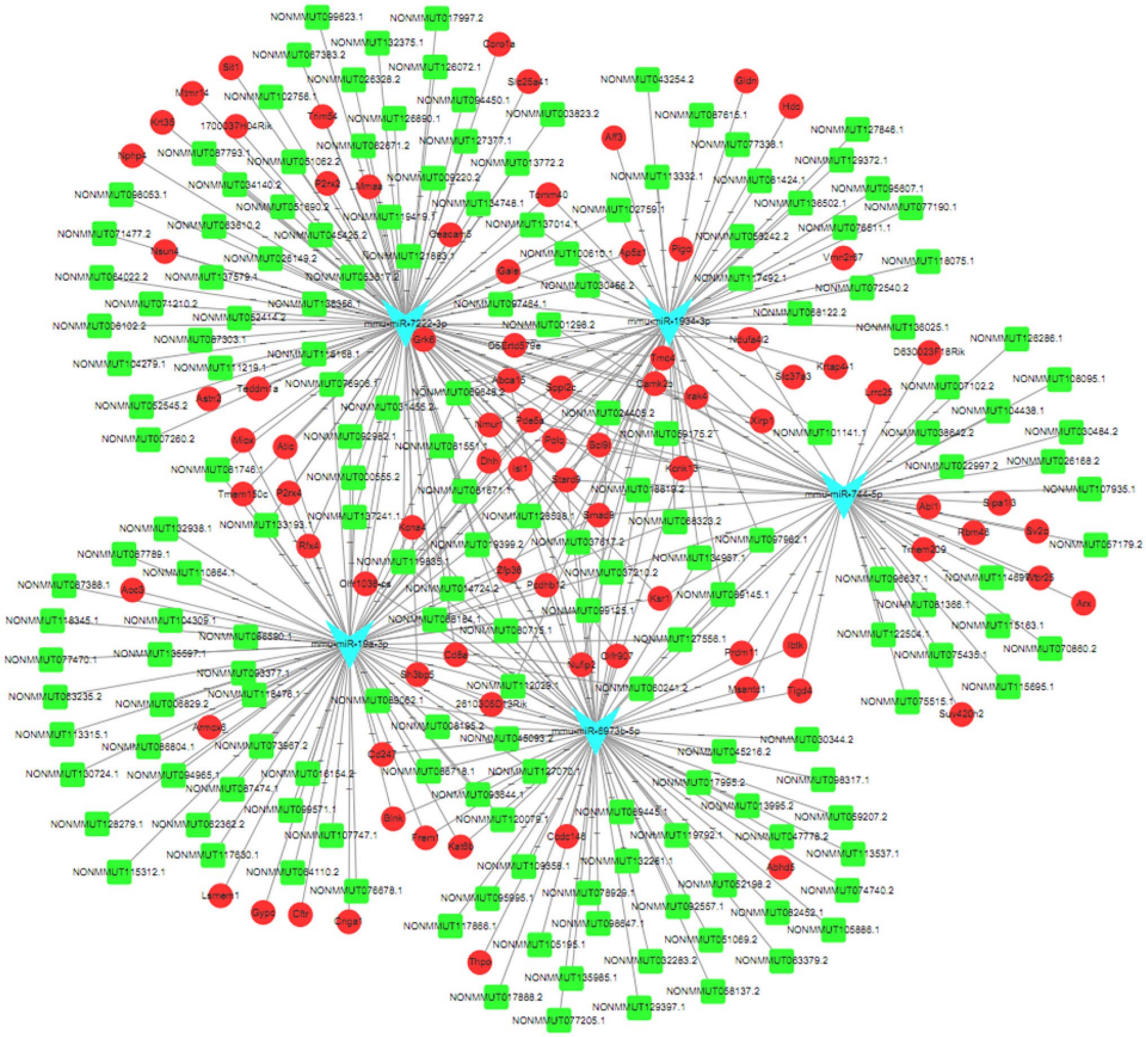
ceRNA network responded to the lncRNA, mRNA, and miRNA variations the LF-rTMS-treated mice as compared to the sham rTMS-treated mice. There were 180 lncRNA nodes, 84 mRNA nodes, and 5 miRNA nodes in this network. Green squares represent lncRNAs, red circles represent coding genes, and blue triangles represent miRNAs

CeRNAs share MREs and modulate RNA transcripts by competitively interacting with general miRNA molecules. CeRNA network was established on the basis of DELs, DEGs, and DEMs in the SE mice treated with LF-rTMS and in the SE mice treated with sham rTMS. Modulation forecasted through miRanda and PCC were between DEMs and the targets (miRNA-mRNA or miRNA-lncRNA). Then these shared couples of DEMs and the targets were picked for constructing ceRNA modulatory network. Next, combined the miRNA-mRNA interplay with lncRNA-miRNA interplay, a network of ceRNA was established by Cytoscape. Nodes in the network indicated lncRNAs, mRNAs, and miRNAs. The linked lines in the network symbolized interaction of these RNAs (Fig. 5). A ceRNA network map was obtained by combining the related pairs obtained above and was visualized them with Cytoscape. There were 269 nodes in this network, including 180 lncRNAs, 5 miRNAs, and 84 mRNAs (Fig. 5).

## Discussion

LncRNAs have been demonstrated to participate in multiple biological processes, including post-transcriptional and epigenetic regulation. It was reported that lncRNA inhibited the development of epilepsy by regulating miRNA to decrease neuronal apoptosis and neuroinflammation in vitro studies (Feng et al. 2020, 2021; Han et al. 2020). However, there was still a lack of global lncRNA-associated crosstalk in the treatment of LF-rTMS for epilepsy.

It is widely believed that miRNAs not only decrease mRNA reliability by linking with miRNA response element (MRE) but also block its translation. Owing to MRE, a single miRNA can target RNAs in “one to multiple.” On the contrary, a single mRNA or LncRNA is modulated by miRNAs in “one to multiple” (Jeyapalan et al. 2011). Hereby, this ceRNA interaction may be a universal post-transcriptional modification. Accumulating evidence suggests that lncRNAs act as endogenous ceRNA through competitive inhibition of miRNA, thereby regulating other transcripts. CeRNA is involved in neuronal development, synaptic transmission, and ion channels related to epileptogenesis. Additionally, it can also regulate neuronal inflammation, neuron apoptosis, and autophagy to delay the progression of epilepsy. The detail functions and special expression pattern of miRNAs and lncRNAs in epilepsy remained largely unclear. However, the tricky ceRNA interplay mechanisms of lncRNA–miRNA-mRNA in LF-rTMS treatment for epilepsy remained to be investigated. In this study, we identified 1029 upregulated and 586 downregulated lncRNAs, 12 upregulated and 5 downregulated miRNAs, 383 upregulated, and 127 downregulated mRNAs in the LF-rTMS-treated mice as compared to the control mice. Although lncRNAs are considered to attract miRNAs competitively and then affect mRNAs expression, their biological characteristics and clinical relevance are still unclear. The constructed ceRNA regulatory network included five miRNAs (mmu-miR-7222-3p, mmu-miR-744-5p, mmu-miR-1934-3p, mmu-miR-19a-3p, and mmu-miR-8973b-5p), but their roles in epilepsy still awaited be further investigated.

Critical nodes identified in some studies and highly connected with other nodes of ceRNA network would be viewed as its topological properties to evaluate the essentiality of genes (Li et al. 2019b). As recently hypothesized, ceRNA is viewed as a new post-transcriptional regulation circuit. The lncRNAs, of this ceRNA network, connect with mRNAs that share MREs (Fan et al. 2018). After decade, hundreds of studies have explored the mechanisms of ceRNA in various neurological diseases, for instance, ischemic stroke and epilepsy (Brennan and Henshall 2020; Homanics et al. 1997; Jiang et al. 2017). To examine lncRNAs markedly linked with LF-rTMS treatment of epilepsy, in this study, the mRNA and lncRNA expression alternation in mice after LF-rTMS, combined with miRNA-target interactions to establish ceRNA network, were utilized to investigate the potential roles of these lncRNAs in the molecular mechanisms of LF-rTMS treatment for epilepsy.

No definite signal pathway has been confirmed to be responsible for LF-rTMS treatment for epilepsy. In the present study, the differentially expressed genes appeared to take part in signaling pathways, involving GABA-A receptor activity, Th17 cell differentiation, T cell receptor signaling pathway, primary immunodeficiency, and so on. GABA is a major neurotransmitter controlling neuronal excitability. The suspected contacts in GABA and epilepsy as well as in GABA and brain development have been recognized (Homanics et al. 1997). Differential co-assembly of α1-GABARs was associated with epileptic encephalopathy (Hannan et al. 2020). Protrudin can modulate seizure activities by regulating GABA receptor (Lu et al. 2019). HMGB1/CXCL12-mediated immunity and Th17 cells were highly suggested as the core of autoimmune epilepsy (Han et al. 2020). Compared with antibody-mediated diseases, T-cell-mediated disease had higher risk of epilepsy (Geis et al. 2019). On the basis of our bioinformatics analysis, we speculated that GABA-A receptor activity, Th17 cell differentiation, and T cell receptor signaling pathway might be responsible pathways involved in the molecular mechanisms of LF-rTMS treatment for epilepsy. Based on the role of LF-rTMS in regulating synaptic-associated protein level, glutamate concentration and GABA receptor, and even affecting GSK, TGF, and other signaling pathways (Dolgova et al. 2021; Tan et al. 2018), we explored the potential mechanisms of miRNA changes after LF-TMS treatment for epilepsy. Among the 12 upregulated and 5 downregulated miRNAs of rTMS treatment group, we searched for several miRNAs that directly or indirectly affect epilepsy. According to previous reports, MiR-28 could up-regulate AQP4 (AQP4) by inhibiting aldehyde dehydrogenase 2 (ALDH2) and promote the recovery of water homeostasis after epilepsy (Hubbard et al. 2016; Li et al. 2018). AQP4 was believed as the core of maintaining water and potassium homeostasis in brain, and AQP4 knockout mice showed spontaneous recurrent seizures with increased frequency and duration (Szu et al. 2020). In addition, mir-138 inhibited axon regeneration (Liu et al. 2013). Furthermore, mir-186 controls the surface expression of GluA2 (ionic glutamate receptor A2) in hippocampal neurons (Silva et al. 2019). Downregulated miR-744 could downregulate Npas4 as an intrinsic regulator of seizures and epilepsy (Choy et al. 2017; Shan et al. 2018). Together, these alterations might represent an intrinsic therapeutic mechanism of LF-rTMS for epilepsy.

In conclusion, a ceRNA based on lncRNA-miRNA-mRNA network was established in the present study, providing molecular mechanisms of LF-rTMS treatment for epilepsy. Furthermore, lncRNA-miRNA-mRNA pairs might view as valuable detecting biomarkers or candidate curative targets for epilepsy.

## Supplementary Information

Below is the link to the electronic supplementary material.

Supplementary file1 (DOCX 235 KB)

Supplementary file2 (DOCX 88 KB)

Supplementary file3 (DOCX 55 KB)

Supplementary file4 (DOCX 27 KB)

Supplementary file5 (PDF 52 KB)

## Data Availability

The data that supports the findings of this study is available from the corresponding author upon reasonable request.
