# Supplementary material for: Transcriptome Sequencing of CeRNA Network Constructing in Status Epilepticus Mice Treated by Low-Frequency Repetitive Transcranial Magnetic Stimulation
Source: J Mol Neurosci. 2023 May 3;73(4-5):316–26. doi: 10.1007/s12031-023-02108-z (PMC10200785; doi:10.1007/s12031-023-02108-z)
Supplement: Supplementary file 1 — Supplementary file1 (DOCX 235 KB) [file 12031_2023_2108_MOESM1_ESM.docx]

**Table** **S1:** **Significantly** **and** **differentially** **expressed** **lncRNAs** **in** **low** **frequency** **rTMS** **and** **sham** **rTMS** **mice.**

| **ProbeName** | **P** **value** | **Fold** **Change** **(abs)** | **Regulation** | **BioType** | **TargetID** |
| --- | --- | --- | --- | --- | --- |
| CUST_OEV3_035361 | 0.043493994 | 2.3440285 | down | noncoding | NONMMUT093889.1 |
| CUST_OEV3_064767 | 0.026282802 | 2.0050964 | up | noncoding | AK140968 |
| CUST_OEV3_092138 | 0.030415043 | 2.4036841 | up | noncoding | XR_377870 |
| CUST_OEV3_033575 | 0.009571797 | 2.4446235 | down | noncoding | NONMMUT096136.1 |
| CUST_OEV3_020285 | 0.04145188 | 6.770221 | up | noncoding | XR_375943 |
| CUST_OEV3_033331 | 0.049772207 | 4.5681434 | down | noncoding | NONMMUT096441.1 |
| CUST_OEV3_001246 | 3.52E-04 | 2.7169788 | up | noncoding | NovelTID_00003317 |
| CUST_OEV3_068290 | 0.016261954 | 2.4578552 | down | noncoding | AK089822 |
| CUST_OEV3_048340 | 0.004617376 | 3.2985222 | up | noncoding | NONMMUT076906.1 |
| CUST_OEV3_003972 | 0.012478541 | 2.5832283 | down | noncoding | NONMMUT135255.1 |
| CUST_OEV3_049856 | 0.023230663 | 2.0199645 | up | noncoding | S89400 |
| CUST_OEV3_008470 | 0.031995017 | 2.1076648 | down | noncoding | NONMMUT129518.1 |
| CUST_OEV3_020523 | 0.0432732 | 2.3882997 | up | noncoding | NONMMUT113442.1 |
| CUST_OEV3_029844 | 0.020138336 | 3.4905155 | up | noncoding | NONMMUT100902.1 |
| CUST_OEV3_038759 | 0.036910675 | 2.8555794 | up | noncoding | NONMMUT089400.1 |
| CUST_OEV3_022856 | 0.03290015 | 2.0086362 | up | noncoding | NONMMUT110179.1 |
| CUST_OEV3_011590 | 0.008503218 | 2.5692694 | down | noncoding | NONMMUT125420.1 |
| CUST_OEV3_009937 | 0.045070168 | 3.1561859 | up | noncoding | NONMMUT127621.1 |
| CUST_OEV3_092630 | 0.049126986 | 2.192381 | up | noncoding | AK050709 |
| CUST_OEV3_037248 | 0.034137264 | 2.3347151 | up | noncoding | NONMMUT091420.1 |
| CUST_OEV3_013798 | 0.003031904 | 4.244375 | up | noncoding | NONMMUT122504.1 |
| CUST_OEV3_051205 | 0.04860963 | 3.2005143 | down | noncoding | NONMMUT070820.2 |
| CUST_OEV3_042304 | 0.012625697 | 2.225155 | up | noncoding | NONMMUT084719.1 |
| CUST_OEV3_069846 | 0.027370956 | 4.894943 | down | noncoding | AK138982 |
| CUST_OEV3_044064 | 0.001730006 | 2.0332143 | up | noncoding | NONMMUT082452.1 |
| CUST_OEV3_027876 | 0.021253467 | 2.4709847 | down | noncoding | NONMMUT103515.1 |
| CUST_OEV3_058958 | 0.034346692 | 2.1867495 | down | noncoding | NONMMUT047415.2 |
| CUST_OEV3_048639 | 0.004270463 | 2.0266492 | up | noncoding | NONMMUT076510.1 |
| CUST_OEV3_049621 | 0.019945795 | 2.7580285 | down | noncoding | NONMMUT075215.1 |
| CUST_OEV3_060489 | 0.029401518 | 6.809523 | down | noncoding | NONMMUT042650.2 |
| CUST_OEV3_092917 | 0.021460919 | 2.7848566 | up | noncoding | BU936458 |
| CUST_OEV3_060926 | 0.008012193 | 3.1204438 | down | noncoding | AK036723 |
| CUST_OEV3_029871 | 0.003200078 | 2.1072557 | down | noncoding | NONMMUT100860.1 |
| CUST_OEV3_002915 | 0.021124233 | 3.1397839 | up | noncoding | NONMMUT136666.1 |
| CUST_OEV3_050644 | 0.041357744 | 3.0437336 | up | noncoding | NONMMUT072551.2 |
| CUST_OEV3_035267 | 0.040254727 | 2.7568254 | down | noncoding | NONMMUT094000.1 |
| CUST_OEV3_007151 | 0.002014028 | 4.136562 | up | noncoding | NONMMUT131103.1 |
| CUST_OEV3_032371 | 0.020467408 | 2.109212 | up | noncoding | NONMMUT097696.1 |
| CUST_OEV3_058210 | 0.038280293 | 3.2173429 | down | noncoding | AK082548 |
| CUST_OEV3_024111 | 0.020454628 | 3.607559 | up | noncoding | NONMMUT108486.1 |
| CUST_OEV3_005184 | 0.049523998 | 2.0491905 | down | noncoding | NONMMUT133679.1 |
| CUST_OEV3_046188 | 0.047297493 | 4.3357034 | up | noncoding | NONMMUT079658.1 |
| CUST_OEV3_005456 | 0.016436642 | 2.640981 | up | noncoding | NONMMUT133321.1 |
| CUST_OEV3_003589 | 0.011370578 | 2.9748802 | down | noncoding | NONMMUT135748.1 |
| CUST_OEV3_015012 | 0.021770515 | 2.4261158 | down | noncoding | NONMMUT120883.1 |
| CUST_OEV3_000941 | 0.049682878 | 2.1772673 | down | noncoding | NovelTID_00004268 |
| CUST_OEV3_033886 | 0.043316655 | 2.5487428 | down | noncoding | NONMMUT095736.1 |
| CUST_OEV3_048897 | 0.027679645 | 2.5218778 | up | noncoding | NONMMUT076161.1 |
| CUST_OEV3_044646 | 0.004869879 | 2.005878 | up | noncoding | XR_380162 |
| CUST_OEV3_044045 | 0.009753755 | 2.4609077 | up | noncoding | NONMMUT082482.1 |
| A_55_P2085460 | 0.002209552 | 8.584222 | up | noncoding | NR_015467 |
| CUST_OEV3_037594 | 0.019489845 | 2.7837384 | down | noncoding | NONMMUT090980.1 |
| CUST_OEV3_026235 | 0.010975714 | 5.096716 | up | noncoding | NONMMUT105623.1 |
| CUST_OEV3_027520 | 0.006324023 | 2.2218914 | down | noncoding | NONMMUT103962.1 |
| CUST_OEV3_009504 | 0.038542338 | 2.6161504 | up | noncoding | NONMMUT128193.1 |
| CUST_OEV3_092233 | 0.004319109 | 13.943758 | up | noncoding | BC072574 |
| CUST_OEV3_024790 | 0.006977179 | 3.6903741 | down | noncoding | NONMMUT107558.1 |
| CUST_OEV3_009942 | 0.034232084 | 2.042385 | down | noncoding | NONMMUT127616.1 |
| CUST_OEV3_064231 | 0.010090753 | 2.6624968 | up | noncoding | AK162857 |
| CUST_OEV3_066285 | 0.009638401 | 4.7974763 | up | noncoding | ENSMUST00000150553 |

| CUST_OEV3_061889 | 0.040653456 | 4.954317 | up | noncoding | AK052813 |
| --- | --- | --- | --- | --- | --- |
| CUST_OEV3_067222 | 0.008463005 | 2.2569764 | up | noncoding | TC1614534 |
| CUST_OEV3_033499 | 0.017526006 | 2.608353 | up | noncoding | NONMMUT096238.1 |
| CUST_OEV3_020521 | 0.026108745 | 2.3890836 | down | noncoding | AK156870 |
| CUST_OEV3_026284 | 0.02992559 | 2.0633965 | up | noncoding | NONMMUT105561.1 |
| CUST_OEV3_059545 | 0.047822665 | 2.2601137 | down | noncoding | AK136902 |
| CUST_OEV3_014025 | 0.046322495 | 3.100565 | down | noncoding | NONMMUT122177.1 |
| CUST_OEV3_023019 | 0.049158543 | 2.0804262 | down | noncoding | NONMMUT109960.1 |
| CUST_OEV3_018548 | 0.025283467 | 4.1895003 | up | noncoding | NONMMUT116120.1 |
| CUST_OEV3_041316 | 0.015921982 | 3.4386578 | up | noncoding | NONMMUT086063.1 |
| CUST_OEV3_032975 | 0.003637479 | 2.1493778 | up | noncoding | NONMMUT096909.1 |
| CUST_OEV3_065075 | 0.026874715 | 3.0124018 | down | noncoding | AK037859 |
| CUST_OEV3_048835 | 0.030271761 | 2.0558748 | up | noncoding | NONMMUT076238.1 |
| CUST_OEV3_090286 | 0.009565447 | 2.0473087 | up | noncoding | AK155887 |
| CUST_OEV3_021006 | 0.03475474 | 2.3121839 | down | noncoding | NONMMUT112800.1 |
| CUST_OEV3_024766 | 0.04002609 | 2.3671665 | down | noncoding | XR_400068 |
| CUST_OEV3_021189 | 0.025214616 | 3.0504405 | up | noncoding | NONMMUT112528.1 |
| CUST_OEV3_091968 | 0.024970958 | 4.5994916 | down | noncoding | AK054385 |
| CUST_OEV3_019436 | 0.029700262 | 4.2045693 | up | noncoding | NONMMUT114926.1 |
| CUST_OEV3_071040 | 0.001417408 | 3.4472585 | up | noncoding | NONMMUT011859.2 |
| CUST_OEV3_034596 | 0.022236627 | 5.8854227 | up | noncoding | NONMMUT094854.1 |
| A_55_P2841581 | 0.048385307 | 2.192327 | down | noncoding | XR_379322 |
| CUST_OEV3_058359 | 0.022158878 | 4.285924 | down | noncoding | AK081632 |
| CUST_OEV3_025250 | 0.04861753 | 2.3973083 | down | noncoding | AK017050 |
| CUST_OEV3_067870 | 0.01279604 | 2.0476575 | down | noncoding | ENSMUST00000183840 |
| CUST_OEV3_048514 | 0.005667415 | 2.2998488 | up | noncoding | NONMMUT076678.1 |
| CUST_OEV3_025961 | 0.029609706 | 2.5444098 | up | noncoding | NONMMUT106011.1 |
| CUST_OEV3_037172 | 0.038360998 | 4.44254 | up | noncoding | XR_382531 |
| CUST_OEV3_061411 | 0.023707923 | 2.432237 | down | noncoding | NONMMUT039777.2 |
| CUST_OEV3_033525 | 0.043760248 | 2.2657275 | down | noncoding | NONMMUT096205.1 |
| CUST_OEV3_008821 | 0.009353943 | 2.6894736 | down | noncoding | NONMMUT129119.1 |
| CUST_OEV3_016251 | 0.044622928 | 2.286238 | down | noncoding | NONMMUT119179.1 |
| CUST_OEV3_003873 | 0.040055677 | 2.8354135 | down | noncoding | NONMMUT135390.1 |
| CUST_OEV3_053161 | 1.12E-04 | 2.176224 | up | noncoding | NONMMUT065274.2 |
| CUST_OEV3_017399 | 0.029326495 | 2.7542944 | up | noncoding | NONMMUT117632.1 |
| CUST_OEV3_091319 | 0.024738848 | 2.2290018 | up | noncoding | AK036198 |
| A_52_P590898 | 0.033463553 | 2.6542938 | down | noncoding | NR_002870 |
| CUST_OEV3_028630 | 0.04588354 | 2.495951 | down | noncoding | NONMMUT102533.1 |
| CUST_OEV3_018605 | 0.031293128 | 2.0440078 | down | noncoding | NONMMUT116046.1 |
| A_66_P139078 | 0.024919752 | 2.0813298 | down | noncoding | NR_045832 |
| CUST_OEV3_012675 | 0.03134648 | 3.0744724 | up | noncoding | NONMMUT123949.1 |
| CUST_OEV3_007193 | 0.010906816 | 4.628869 | up | noncoding | NONMMUT131042.1 |
| CUST_OEV3_002157 | 0.04503763 | 4.521842 | down | noncoding | NovelTID_00000115 |
| CUST_OEV3_031612 | 0.005377691 | 5.825567 | up | noncoding | NONMMUT098646.1 |
| CUST_OEV3_053819 | 3.72E-04 | 2.3300242 | down | noncoding | AK080945 |
| CUST_OEV3_058095 | 0.036969803 | 2.5210307 | up | noncoding | NONMMUT050016.2 |
| CUST_OEV3_035326 | 0.041141015 | 3.0252151 | up | noncoding | NONMMUT093926.1 |
| CUST_OEV3_038856 | 0.040211175 | 2.4168782 | down | noncoding | NONMMUT089271.1 |
| A_55_P2041700 | 0.029600324 | 2.220616 | down | noncoding | NR_015348 |
| CUST_OEV3_009120 | 0.018775424 | 2.6120567 | up | noncoding | NONMMUT128704.1 |
| CUST_OEV3_019124 | 0.018471573 | 2.3055525 | up | noncoding | NONMMUT115347.1 |
| CUST_OEV3_012628 | 0.003981114 | 2.2403972 | down | noncoding | NONMMUT124019.1 |
| CUST_OEV3_031349 | 0.017115567 | 2.3147378 | up | noncoding | XR_384970 |
| CUST_OEV3_071502 | 0.028253833 | 3.8577104 | down | noncoding | NONMMUT010421.2 |
| A_66_P134821 | 0.025351249 | 2.0688636 | up | noncoding | NR_040510 |
| CUST_OEV3_051796 | 0.02698657 | 3.0820665 | up | noncoding | AK138634 |
| CUST_OEV3_005524 | 0.034970347 | 3.0736952 | up | noncoding | NONMMUT133228.1 |
| CUST_OEV3_033598 | 0.022404034 | 2.807022 | down | noncoding | NONMMUT096109.1 |
| CUST_OEV3_023321 | 0.03461342 | 2.264687 | down | noncoding | NONMMUT109570.1 |
| CUST_OEV3_007384 | 0.025160937 | 2.1044571 | up | noncoding | NONMMUT130818.1 |
| CUST_OEV3_021425 | 0.01603029 | 2.172408 | down | noncoding | NONMMUT112186.1 |
| CUST_OEV3_009781 | 0.008831749 | 2.0025585 | up | noncoding | NONMMUT127846.1 |
| CUST_OEV3_039386 | 0.021919658 | 2.082815 | down | noncoding | NONMMUT088570.1 |

| CUST_OEV3_007951 | 0.023579707 | 2.3345323 | down | noncoding | NONMMUT130175.1 |
| --- | --- | --- | --- | --- | --- |
| CUST_OEV3_039981 | 0.002616605 | 3.4846504 | up | noncoding | NONMMUT087793.1 |
| CUST_OEV3_016047 | 0.040919688 | 2.7002645 | down | noncoding | NONMMUT119472.1 |
| A_52_P296452 | 0.012066584 | 6.5348697 | up | noncoding | NR_040461 |
| CUST_OEV3_020257 | 0.03504056 | 2.2401845 | down | noncoding | NONMMUT113830.1 |
| CUST_OEV3_006184 | 0.013488572 | 6.748848 | up | noncoding | NONMMUT132375.1 |
| CUST_OEV3_030635 | 0.020589896 | 3.0695999 | up | noncoding | NONMMUT099886.1 |
| CUST_OEV3_003719 | 0.012875289 | 2.1124547 | up | noncoding | NONMMUT135566.1 |
| CUST_OEV3_069999 | 0.011363274 | 4.709744 | up | noncoding | AK146862 |
| CUST_OEV3_005760 | 5.60E-04 | 15.997673 | up | noncoding | NONMMUT132938.1 |
| A_55_P2750839 | 0.005717335 | 4.6769643 | up | noncoding | XR_380118 |
| CUST_OEV3_037308 | 0.047473095 | 2.091732 | up | noncoding | NONMMUT091342.1 |
| CUST_OEV3_018466 | 0.048392147 | 7.142919 | down | noncoding | NONMMUT116230.1 |
| A_66_P131815 | 0.029524371 | 2.6928108 | up | noncoding | NR_126072 |
| CUST_OEV3_009434 | 0.001459506 | 12.037275 | up | noncoding | NONMMUT128279.1 |
| CUST_OEV3_045952 | 0.03048696 | 2.8329053 | up | noncoding | NONMMUT079977.1 |
| CUST_OEV3_043011 | 0.046891406 | 6.9614906 | down | noncoding | NONMMUT083794.1 |
| CUST_OEV3_055579 | 0.045358915 | 5.922549 | up | noncoding | AK041698 |
| CUST_OEV3_040450 | 0.024048325 | 4.2659206 | up | noncoding | NONMMUT087191.1 |
| CUST_OEV3_042095 | 0.012388887 | 5.268998 | up | noncoding | NONMMUT084995.1 |
| CUST_OEV3_011718 | 0.01827195 | 2.6728878 | up | noncoding | NONMMUT125259.1 |
| CUST_OEV3_014050 | 0.005992675 | 4.3242207 | down | noncoding | NONMMUT122141.1 |
| CUST_OEV3_090426 | 0.005253127 | 2.1199946 | up | noncoding | AK015600 |
| CUST_OEV3_057141 | 0.00748352 | 2.2695627 | up | noncoding | AK007181 |
| CUST_OEV3_027361 | 0.010811302 | 2.2172556 | down | noncoding | NONMMUT104156.1 |
| CUST_OEV3_043268 | 0.009470816 | 2.1542509 | up | noncoding | NONMMUT083455.1 |
| CUST_OEV3_020206 | 0.038913764 | 2.2356703 | down | noncoding | NONMMUT113898.1 |
| CUST_OEV3_018234 | 0.002354474 | 12.774076 | up | noncoding | NONMMUT116548.1 |
| CUST_OEV3_093684 | 0.032162014 | 2.7849889 | down | noncoding | TC1665813 |
| CUST_OEV3_001776 | 0.006187272 | 2.3205519 | up | noncoding | XR_381391 |
| CUST_OEV3_021657 | 0.03149472 | 4.369956 | up | noncoding | NONMMUT111868.1 |
| CUST_OEV3_066573 | 0.008278311 | 2.169411 | up | noncoding | XR_384454 |
| CUST_OEV3_026425 | 0.010567424 | 3.4453528 | up | noncoding | NONMMUT105382.1 |
| CUST_OEV3_074776 | 0.045748148 | 2.1722882 | down | noncoding | NONMMUT000593.2 |
| CUST_OEV3_032012 | 0.014618419 | 14.088203 | up | noncoding | NONMMUT098158.1 |
| CUST_OEV3_032078 | 0.026676025 | 2.1449728 | down | noncoding | NONMMUT098078.1 |
| CUST_OEV3_034426 | 0.011951157 | 8.779745 | up | noncoding | NONMMUT095069.1 |
| CUST_OEV3_018874 | 0.048111536 | 2.4361365 | up | noncoding | NONMMUT115678.1 |
| CUST_OEV3_044408 | 0.040436212 | 2.8809462 | down | noncoding | NONMMUT081988.1 |
| CUST_OEV3_002517 | 0.021721065 | 3.0715005 | down | noncoding | NONMMUT137211.1 |
| CUST_OEV3_045434 | 0.031990487 | 2.4684653 | down | noncoding | NONMMUT080658.1 |
| CUST_OEV3_022950 | 0.043415856 | 3.3824573 | down | noncoding | NONMMUT110046.1 |
| A_66_P136432 | 0.005206568 | 5.5749893 | up | noncoding | XR_869600 |
| CUST_OEV3_072088 | 0.02997334 | 2.4662411 | up | noncoding | NONMMUT008802.2 |
| CUST_OEV3_021943 | 0.012294368 | 3.9537692 | up | noncoding | NONMMUT111453.1 |
| A_52_P474699 | 0.014355974 | 2.7054298 | up | noncoding | XR_401822 |
| CUST_OEV3_007651 | 0.049881745 | 3.2167985 | up | noncoding | NONMMUT130520.1 |
| CUST_OEV3_004865 | 0.012948447 | 2.519395 | down | noncoding | NONMMUT134097.1 |
| CUST_OEV3_019770 | 0.019208 | 2.1408052 | up | noncoding | NONMMUT114449.1 |
| CUST_OEV3_008507 | 0.021862071 | 7.361654 | up | noncoding | NONMMUT129481.1 |
| CUST_OEV3_053549 | 0.025341796 | 2.5326014 | down | noncoding | NONMMUT064015.2 |
| CUST_OEV3_000802 | 0.01792857 | 2.6592383 | down | noncoding | NovelTID_00004638 |
| CUST_OEV3_009899 | 0.046967957 | 2.161608 | down | noncoding | NONMMUT127671.1 |
| CUST_OEV3_067882 | 0.03901678 | 2.2149615 | up | noncoding | AK081591 |
| CUST_OEV3_000366 | 0.019950107 | 2.9098082 | up | noncoding | NovelTID_00006060 |
| CUST_OEV3_070599 | 0.043895923 | 2.117112 | up | noncoding | AK007203 |
| CUST_OEV3_008569 | 0.031999093 | 2.3487444 | up | noncoding | NONMMUT129419.1 |
| CUST_OEV3_007998 | 0.020722764 | 2.5950184 | down | noncoding | XR_378837 |
| CUST_OEV3_025125 | 0.009440248 | 3.018712 | down | noncoding | NONMMUT107118.1 |
| A_51_P199175 | 0.027496602 | 2.095486 | up | noncoding | NR_045040 |
| CUST_OEV3_068270 | 0.005661237 | 2.3040752 | down | noncoding | XR_383319 |
| CUST_OEV3_039330 | 0.001151183 | 5.0476356 | up | noncoding | NONMMUT088643.1 |
| CUST_OEV3_005545 | 0.023233153 | 6.129762 | up | noncoding | NONMMUT133202.1 |

| A_55_P2403620 | 0.021531342 | 2.6747694 | down | noncoding | NR_033541 |
| --- | --- | --- | --- | --- | --- |
| CUST_OEV3_053761 | 0.009645292 | 2.4565568 | up | noncoding | NONMMUT063379.2 |
| CUST_OEV3_065186 | 0.026585381 | 2.5584586 | up | noncoding | NONMMUT029032.2 |
| CUST_OEV3_017197 | 0.004622772 | 2.0222764 | down | noncoding | TC1632302 |
| CUST_OEV3_074931 | 0.034190223 | 2.9709694 | up | noncoding | AK153594 |
| CUST_OEV3_026290 | 0.031625487 | 2.7148871 | up | noncoding | NONMMUT105555.1 |
| CUST_OEV3_059326 | 0.045547705 | 2.120481 | up | noncoding | AK051427 |
| CUST_OEV3_007375 | 0.007182803 | 2.047084 | down | noncoding | NONMMUT130827.1 |
| CUST_OEV3_020392 | 0.02885017 | 5.240364 | up | noncoding | NONMMUT113628.1 |
| CUST_OEV3_005220 | 0.019204943 | 2.5908217 | up | noncoding | NONMMUT133635.1 |
| A_55_P2376011 | 0.00601445 | 2.110066 | down | noncoding | NR_131129 |
| CUST_OEV3_002981 | 0.046043072 | 2.389641 | down | noncoding | NONMMUT136566.1 |
| CUST_OEV3_035564 | 0.004272673 | 3.2350261 | up | noncoding | NONMMUT093644.1 |
| CUST_OEV3_093820 | 0.049343035 | 2.6258042 | down | noncoding | ENSMUST00000122590.1 |
| CUST_OEV3_040550 | 0.013614543 | 3.0230832 | down | noncoding | NONMMUT087063.1 |
| CUST_OEV3_064939 | 0.026561318 | 2.4740543 | up | noncoding | TC1720294 |
| CUST_OEV3_052081 | 0.006220912 | 2.139948 | up | noncoding | AK161954 |
| A_66_P100812 | 0.001537861 | 3.5829873 | up | noncoding | NR_126088 |
| A_55_P2585870 | 0.018564533 | 4.591721 | up | noncoding | XR_387088 |
| CUST_OEV3_072197 | 0.048369166 | 2.036015 | down | noncoding | AK084330 |
| CUST_OEV3_024735 | 0.011228063 | 3.008946 | down | noncoding | NONMMUT107621.1 |
| CUST_OEV3_008168 | 0.025361 | 2.8553443 | down | noncoding | NONMMUT129899.1 |
| CUST_OEV3_008403 | 0.01832288 | 5.925665 | up | noncoding | NONMMUT129596.1 |
| CUST_OEV3_048113 | 0.003448982 | 2.2623827 | up | noncoding | NONMMUT077205.1 |
| CUST_OEV3_037171 | 0.034432456 | 6.4720464 | up | noncoding | XR_382531 |
| A_51_P441745 | 0.041657392 | 2.366425 | down | noncoding | XR_168507 |
| CUST_OEV3_090189 | 0.023970906 | 2.3294454 | up | noncoding | KnowTID_00006212 |
| CUST_OEV3_044074 | 0.03448371 | 2.902478 | up | noncoding | NONMMUT082440.1 |
| CUST_OEV3_017662 | 0.047675107 | 2.0222104 | up | noncoding | NONMMUT117313.1 |
| CUST_OEV3_010200 | 0.009766417 | 2.8030252 | up | noncoding | NONMMUT127267.1 |
| CUST_OEV3_021545 | 0.003455055 | 4.6962533 | up | noncoding | NONMMUT112029.1 |
| CUST_OEV3_002624 | 0.044342097 | 3.2105591 | down | noncoding | NONMMUT137068.1 |
| CUST_OEV3_005091 | 0.017424323 | 2.1553648 | down | noncoding | NONMMUT133809.1 |
| CUST_OEV3_071622 | 0.026577178 | 2.2922585 | down | noncoding | AK083550 |
| CUST_OEV3_058689 | 0.037831485 | 2.36988 | up | noncoding | NONMMUT048070.2 |
| CUST_OEV3_051584 | 0.003168357 | 2.175374 | up | noncoding | AK038248 |
| CUST_OEV3_041058 | 0.017743934 | 2.2942398 | down | noncoding | NONMMUT086379.1 |
| CUST_OEV3_003519 | 0.019554587 | 3.447058 | down | noncoding | NONMMUT135841.1 |
| CUST_OEV3_010503 | 9.95E-04 | 2.5388873 | down | noncoding | NONMMUT126843.1 |
| A_66_P119394 | 0.038773373 | 4.70975 | up | noncoding | NR_002857 |
| CUST_OEV3_051266 | 0.027393745 | 2.2917624 | up | noncoding | AK136595 |
| CUST_OEV3_025976 | 0.030054484 | 3.5562296 | up | noncoding | NONMMUT105993.1 |
| CUST_OEV3_058619 | 0.036532834 | 2.070259 | down | noncoding | AK143955 |
| CUST_OEV3_013547 | 0.017064473 | 3.0050275 | up | noncoding | NONMMUT122823.1 |
| CUST_OEV3_026291 | 0.02236325 | 2.0260684 | up | noncoding | NONMMUT105554.1 |
| CUST_OEV3_020749 | 0.045264527 | 2.123479 | down | noncoding | NONMMUT113133.1 |
| CUST_OEV3_093252 | 0.008975075 | 2.2714946 | up | noncoding | ENSMUST00000162316 |
| CUST_OEV3_051347 | 0.04098887 | 2.0007293 | up | noncoding | AK141005 |
| CUST_OEV3_010812 | 0.025219647 | 2.33134 | up | noncoding | NONMMUT126391.1 |
| CUST_OEV3_067150 | 0.03503382 | 3.0240695 | down | noncoding | AK034004 |
| CUST_OEV3_008481 | 0.01906187 | 3.9846406 | up | noncoding | NONMMUT129507.1 |
| CUST_OEV3_093518 | 0.006571859 | 6.5343504 | up | noncoding | NR_033324 |
| CUST_OEV3_044547 | 0.029928705 | 2.1614194 | up | noncoding | NONMMUT081808.1 |
| CUST_OEV3_027126 | 0.017797293 | 2.07511 | down | noncoding | NONMMUT104452.1 |
| CUST_OEV3_005205 | 0.03457998 | 2.0267255 | down | noncoding | XR_406094 |
| CUST_OEV3_043515 | 0.010497202 | 2.831103 | down | noncoding | NONMMUT083128.1 |
| A_55_P2761935 | 0.023190513 | 4.796233 | down | noncoding | XR_865015 |
| CUST_OEV3_047625 | 0.034639247 | 2.0786715 | up | noncoding | NONMMUT077855.1 |
| CUST_OEV3_067791 | 0.029114941 | 3.0348766 | up | noncoding | AK038008 |
| CUST_OEV3_006548 | 6.74E-04 | 5.2742558 | up | noncoding | NONMMUT131893.1 |
| CUST_OEV3_092594 | 0.03878382 | 2.8656638 | up | noncoding | AK081955 |
| CUST_OEV3_060316 | 0.007712343 | 2.370718 | up | noncoding | AK040638 |
| CUST_OEV3_070704 | 0.022293668 | 2.0131786 | down | noncoding | XR_389226 |

| CUST_OEV3_011667 | 0.011288406 | 2.737308 | up | noncoding | NONMMUT125323.1 |
| --- | --- | --- | --- | --- | --- |
| CUST_OEV3_017204 | 7.75E-04 | 4.523309 | up | noncoding | TC1776918 |
| CUST_OEV3_093312 | 0.033024702 | 2.1497443 | up | noncoding | ENSMUST00000157791.1 |
| CUST_OEV3_049398 | 0.002206814 | 16.596096 | up | noncoding | NONMMUT075515.1 |
| A_55_P2509964 | 0.00364772 | 2.681393 | up | noncoding | NR_045190 |
| CUST_OEV3_005575 | 0.028138123 | 2.0001414 | down | noncoding | NONMMUT133167.1 |
| CUST_OEV3_016326 | 0.023482386 | 3.2027228 | down | noncoding | NONMMUT119055.1 |
| CUST_OEV3_032200 | 0.020586949 | 2.9581406 | up | noncoding | NONMMUT097903.1 |
| CUST_OEV3_048024 | 0.002306848 | 8.664177 | up | noncoding | TC1640327 |
| CUST_OEV3_066990 | 0.032310724 | 2.1749299 | down | noncoding | AK013013 |
| CUST_OEV3_013427 | 0.015271585 | 3.1804838 | up | noncoding | NONMMUT122978.1 |
| CUST_OEV3_035220 | 0.04157766 | 2.2065482 | down | noncoding | NONMMUT094060.1 |
| A_55_P2035757 | 0.027576126 | 2.5006595 | down | noncoding | NR_026561 |
| CUST_OEV3_092302 | 0.013995075 | 2.492814 | up | noncoding | ENSMUST00000202075.1 |
| CUST_OEV3_055219 | 0.039757077 | 2.652382 | down | noncoding | NONMMUT058346.2 |
| CUST_OEV3_020952 | 0.02872092 | 2.1511908 | down | noncoding | NONMMUT112870.1 |
| CUST_OEV3_046716 | 0.019281056 | 2.6656883 | up | noncoding | CN836058 |
| CUST_OEV3_065020 | 0.023086393 | 2.153286 | down | noncoding | AK046960 |
| CUST_OEV3_016248 | 0.015665965 | 2.7651079 | up | noncoding | NONMMUT119182.1 |
| CUST_OEV3_007182 | 0.017750116 | 8.896399 | down | noncoding | NONMMUT131055.1 |
| CUST_OEV3_034497 | 0.001143464 | 5.149461 | up | noncoding | NONMMUT094965.1 |
| CUST_OEV3_042995 | 0.012061142 | 2.9068408 | up | noncoding | NONMMUT083816.1 |
| CUST_OEV3_073964 | 0.024561057 | 2.232631 | down | noncoding | AK148560 |
| CUST_OEV3_004185 | 0.003801136 | 5.726628 | up | noncoding | NONMMUT134967.1 |
| CUST_OEV3_066174 | 0.025942678 | 2.4018235 | up | noncoding | AK141655 |
| A_65_P18181 | 0.0415535 | 2.1816077 | up | noncoding | NR_073425 |
| CUST_OEV3_032091 | 0.003324524 | 8.120368 | up | noncoding | NONMMUT098053.1 |
| CUST_OEV3_040288 | 0.0010611 | 6.1086664 | up | noncoding | NONMMUT087388.1 |
| CUST_OEV3_063799 | 0.041933246 | 6.1906667 | up | noncoding | AK156467 |
| A_55_P2550910 | 0.0038932 | 2.536048 | up | noncoding | XR_871362 |
| CUST_OEV3_053758 | 0.003302897 | 4.0166264 | down | noncoding | NONMMUT063391.2 |
| CUST_OEV3_069124 | 0.028508332 | 2.0178292 | up | noncoding | AK150676 |
| CUST_OEV3_069452 | 0.028720671 | 7.603142 | down | noncoding | NONMMUT016592.2 |
| CUST_OEV3_071687 | 0.034847572 | 4.2280498 | down | noncoding | AK089832 |
| CUST_OEV3_043143 | 0.012115018 | 2.9222093 | up | noncoding | XR_388623 |
| CUST_OEV3_040224 | 0.007240145 | 2.7221043 | up | noncoding | NONMMUT087474.1 |
| CUST_OEV3_068971 | 0.020931566 | 4.216433 | up | noncoding | AK085352 |
| CUST_OEV3_006273 | 0.013107491 | 2.1807358 | up | noncoding | NONMMUT132262.1 |
| CUST_OEV3_003891 | 0.038357385 | 2.218628 | up | noncoding | NONMMUT135364.1 |
| CUST_OEV3_036099 | 0.008825495 | 3.5621703 | up | noncoding | NONMMUT092982.1 |
| CUST_OEV3_072743 | 0.041555308 | 2.4356108 | up | noncoding | AK140168 |
| CUST_OEV3_048078 | 0.03407038 | 2.078793 | down | noncoding | NONMMUT077252.1 |
| CUST_OEV3_029724 | 0.006399886 | 12.890371 | up | noncoding | NONMMUT101082.1 |
| CUST_OEV3_040474 | 0.009576869 | 2.59957 | down | noncoding | NONMMUT087154.1 |
| CUST_OEV3_021367 | 0.047242623 | 2.1643853 | up | noncoding | NONMMUT112271.1 |
| CUST_OEV3_048484 | 0.018261613 | 2.449315 | up | noncoding | NONMMUT076722.1 |
| CUST_OEV3_024557 | 0.026380224 | 2.2218597 | down | noncoding | NONMMUT107862.1 |
| CUST_OEV3_011427 | 0.01685749 | 4.605638 | up | noncoding | NONMMUT125636.1 |
| CUST_OEV3_020216 | 0.009815931 | 2.0283673 | up | noncoding | XR_375991 |
| CUST_OEV3_007224 | 0.010970878 | 2.3780794 | up | noncoding | NONMMUT131004.1 |
| CUST_OEV3_049009 | 0.03531738 | 4.500795 | up | noncoding | XR_387272 |
| CUST_OEV3_071950 | 0.033868328 | 2.7843373 | down | noncoding | NONMMUT009170.2 |
| CUST_OEV3_041155 | 0.024415005 | 2.2086468 | up | noncoding | NONMMUT086266.1 |
| CUST_OEV3_006989 | 0.028187795 | 2.5065532 | down | noncoding | NONMMUT131317.1 |
| CUST_OEV3_041468 | 0.012622221 | 2.3250792 | down | noncoding | NONMMUT085849.1 |
| CUST_OEV3_019359 | 0.048111048 | 2.5035026 | down | noncoding | NONMMUT115031.1 |
| CUST_OEV3_014628 | 0.001257799 | 2.7794495 | up | noncoding | NONMMUT121375.1 |
| CUST_OEV3_019796 | 0.047593154 | 2.0994458 | up | noncoding | NONMMUT114421.1 |
| CUST_OEV3_073532 | 0.040626194 | 2.1749225 | down | noncoding | AK081155 |
| CUST_OEV3_068074 | 0.03626128 | 2.1856978 | down | noncoding | NONMMUT020768.2 |
| CUST_OEV3_009448 | 0.03565223 | 4.083799 | up | noncoding | NONMMUT128265.1 |
| CUST_OEV3_058099 | 0.025475742 | 2.6556294 | down | noncoding | NONMMUT050006.2 |
| A_66_P130890 | 0.039611705 | 3.3733954 | up | noncoding | XR_863709 |

| CUST_OEV3_034380 | 0.020375341 | 5.5654016 | up | noncoding | NONMMUT095127.1 |
| --- | --- | --- | --- | --- | --- |
| CUST_OEV3_013780 | 0.047896907 | 2.21609 | up | noncoding | NONMMUT122527.1 |
| CUST_OEV3_024169 | 0.03331731 | 3.5885837 | up | noncoding | NONMMUT108412.1 |
| CUST_OEV3_013473 | 0.0492099 | 3.0726054 | down | noncoding | NONMMUT122923.1 |
| CUST_OEV3_023958 | 0.027118884 | 3.1107612 | up | noncoding | NONMMUT108719.1 |
| CUST_OEV3_012727 | 0.02772871 | 3.5619473 | down | noncoding | NONMMUT123888.1 |
| CUST_OEV3_033099 | 0.04418209 | 2.2259421 | down | noncoding | XR_383858 |
| CUST_OEV3_018556 | 0.035813157 | 3.367362 | up | noncoding | NONMMUT116111.1 |
| CUST_OEV3_045288 | 0.029076034 | 2.0450132 | down | noncoding | NONMMUT080845.1 |
| CUST_OEV3_023142 | 0.028040674 | 3.2445493 | up | noncoding | NONMMUT109804.1 |
| CUST_OEV3_013356 | 0.009555084 | 2.3773084 | up | noncoding | NONMMUT123065.1 |
| CUST_OEV3_090356 | 5.09E-04 | 5.1676974 | up | noncoding | AK037544 |
| CUST_OEV3_035298 | 0.040971085 | 3.390484 | down | noncoding | NONMMUT093963.1 |
| CUST_OEV3_018788 | 0.03950038 | 2.570862 | up | noncoding | NONMMUT115788.1 |
| CUST_OEV3_003370 | 0.044858355 | 2.183203 | down | noncoding | NONMMUT136026.1 |
| CUST_OEV3_044540 | 0.023013841 | 2.2047215 | down | noncoding | NONMMUT081815.1 |
| CUST_OEV3_054568 | 0.013782527 | 2.467323 | up | noncoding | NONMMUT060495.2 |
| CUST_OEV3_014169 | 0.009740883 | 2.1347294 | up | noncoding | NONMMUT121994.1 |
| CUST_OEV3_028211 | 0.03522146 | 4.413737 | up | noncoding | NONMMUT103098.1 |
| CUST_OEV3_038050 | 0.024963407 | 7.011059 | down | noncoding | NONMMUT090376.1 |
| CUST_OEV3_060070 | 0.018897118 | 3.424425 | down | noncoding | AK009453 |
| CUST_OEV3_004494 | 0.026690047 | 2.9916198 | up | noncoding | NONMMUT134550.1 |
| CUST_OEV3_058595 | 0.03420839 | 3.6152132 | down | noncoding | AK043368 |
| CUST_OEV3_020822 | 0.041046433 | 2.1910803 | down | noncoding | NONMMUT113027.1 |
| CUST_OEV3_064419 | 0.030978302 | 2.604646 | down | noncoding | AK042614 |
| CUST_OEV3_010613 | 0.005626638 | 5.890946 | up | noncoding | NONMMUT126683.1 |
| CUST_OEV3_050944 | 0.009489497 | 2.3911297 | up | noncoding | AK137951 |
| CUST_OEV3_072398 | 0.019350091 | 2.1545393 | up | noncoding | AK137173 |
| CUST_OEV3_008809 | 0.01061149 | 2.776984 | up | noncoding | XR_387904 |
| CUST_OEV3_019192 | 0.04780655 | 2.450619 | down | noncoding | NONMMUT115259.1 |
| CUST_OEV3_069088 | 0.010379992 | 2.166391 | down | noncoding | AK135300 |
| CUST_OEV3_053516 | 0.00785615 | 2.5944378 | up | noncoding | AK148588 |
| CUST_OEV3_068519 | 0.016559953 | 2.5792003 | up | noncoding | AK051144 |
| CUST_OEV3_030215 | 0.035060298 | 2.1145265 | up | noncoding | NONMMUT100383.1 |
| A_55_P2835846 | 0.017432014 | 2.2135637 | up | noncoding | XR_871169 |
| A_52_P598530 | 0.018397287 | 8.348536 | down | noncoding | NR_040578 |
| CUST_OEV3_019351 | 0.046984196 | 2.1171134 | down | noncoding | NONMMUT115044.1 |
| CUST_OEV3_010982 | 0.0402794 | 3.6363347 | down | noncoding | NONMMUT126154.1 |
| A_55_P2067463 | 0.043309793 | 3.0286758 | down | noncoding | NR_038059 |
| CUST_OEV3_040955 | 0.011670396 | 2.2355535 | down | noncoding | NONMMUT086550.1 |
| CUST_OEV3_057296 | 0.04937099 | 3.6511326 | up | noncoding | AK150257 |
| CUST_OEV3_044509 | 0.034108922 | 3.5919864 | up | noncoding | NONMMUT081861.1 |
| CUST_OEV3_007770 | 0.02198039 | 3.4719446 | up | noncoding | NONMMUT130376.1 |
| CUST_OEV3_054470 | 0.0358779 | 4.089721 | up | noncoding | NONMMUT060882.2 |
| CUST_OEV3_063262 | 0.017621893 | 3.5247643 | down | noncoding | NONMMUT034571.2 |
| CUST_OEV3_032546 | 0.00610082 | 2.437974 | up | noncoding | NONMMUT097460.1 |
| CUST_OEV3_067753 | 0.04005933 | 3.0970683 | up | noncoding | NR_015485 |
| CUST_OEV3_073331 | 0.011698258 | 2.8534913 | up | noncoding | AK030930 |
| CUST_OEV3_000621 | 0.007898137 | 2.5006676 | down | noncoding | NovelTID_00005291 |
| CUST_OEV3_037946 | 0.030934336 | 2.0612247 | up | noncoding | NONMMUT090518.1 |
| CUST_OEV3_018340 | 0.03560136 | 2.1923683 | up | noncoding | NONMMUT116403.1 |
| CUST_OEV3_071533 | 0.020451704 | 3.0390217 | up | noncoding | AK039933 |
| CUST_OEV3_052196 | 0.029532861 | 2.3851593 | down | noncoding | NONMMUT067962.2 |
| CUST_OEV3_012803 | 0.018806038 | 4.662464 | down | noncoding | NONMMUT123786.1 |
| CUST_OEV3_059523 | 0.030902501 | 2.1662762 | down | noncoding | NONMMUT045814.2 |
| CUST_OEV3_037439 | 0.01541942 | 2.1819363 | down | noncoding | NONMMUT091189.1 |
| CUST_OEV3_020997 | 0.01633068 | 2.5630007 | down | noncoding | NONMMUT112812.1 |
| CUST_OEV3_022527 | 0.0162999 | 2.634088 | down | noncoding | NONMMUT110675.1 |
| CUST_OEV3_038551 | 0.03175855 | 2.788007 | down | noncoding | NONMMUT089689.1 |
| CUST_OEV3_017677 | 0.036416117 | 2.0510778 | down | noncoding | NONMMUT117295.1 |
| CUST_OEV3_001943 | 0.003218 | 7.530026 | up | noncoding | NovelTID_00000995 |
| CUST_OEV3_013438 | 0.004497206 | 4.157274 | down | noncoding | NONMMUT122964.1 |
| A_66_P116683 | 0.032793082 | 2.479789 | up | noncoding | NR_045629 |

| CUST_OEV3_092915 | 0.015402174 | 2.44949 | down | noncoding | ENSMUST00000185270.1 |
| --- | --- | --- | --- | --- | --- |
| CUST_OEV3_035693 | 0.008563023 | 2.0670474 | down | noncoding | NONMMUT093481.1 |
| CUST_OEV3_026891 | 0.013592589 | 2.3464057 | down | noncoding | NONMMUT104737.1 |
| A_51_P246104 | 0.04392013 | 2.178566 | up | noncoding | NR_103800 |
| CUST_OEV3_029726 | 0.023479518 | 2.8558247 | down | noncoding | NONMMUT101079.1 |
| CUST_OEV3_011379 | 0.030173507 | 2.3827724 | down | noncoding | NONMMUT125688.1 |
| CUST_OEV3_026387 | 0.015606253 | 2.6421309 | up | noncoding | NONMMUT105436.1 |
| CUST_OEV3_015117 | 0.033983503 | 7.971099 | up | noncoding | NONMMUT120753.1 |
| CUST_OEV3_024355 | 0.006600028 | 2.4317136 | down | noncoding | NONMMUT108148.1 |
| CUST_OEV3_063554 | 0.002471039 | 12.1316805 | up | noncoding | NONMMUT033879.2 |
| CUST_OEV3_031213 | 0.008863708 | 2.7863472 | up | noncoding | NONMMUT099124.1 |
| CUST_OEV3_041720 | 0.00956054 | 2.0772805 | down | noncoding | NONMMUT085494.1 |
| CUST_OEV3_014488 | 0.028126908 | 2.7203743 | down | noncoding | NONMMUT121566.1 |
| CUST_OEV3_017939 | 0.005195502 | 2.8490536 | down | noncoding | NONMMUT116940.1 |
| CUST_OEV3_025682 | 0.008668308 | 2.8027418 | up | noncoding | NONMMUT106373.1 |
| CUST_OEV3_001741 | 0.017380036 | 2.0790815 | down | noncoding | NovelTID_00001552 |
| CUST_OEV3_013812 | 0.020348376 | 2.3367996 | down | noncoding | NONMMUT122478.1 |
| CUST_OEV3_028006 | 0.028403904 | 3.9218094 | up | noncoding | NONMMUT103354.1 |
| CUST_OEV3_022714 | 0.021586709 | 2.0536108 | down | noncoding | NONMMUT110404.1 |
| CUST_OEV3_012805 | 0.016755817 | 2.0547018 | up | noncoding | NONMMUT123784.1 |
| CUST_OEV3_029495 | 0.022570329 | 2.8768022 | down | noncoding | NONMMUT101370.1 |
| CUST_OEV3_032959 | 0.04388019 | 2.83157 | up | noncoding | NONMMUT096925.1 |
| CUST_OEV3_026128 | 0.041341007 | 2.919514 | down | noncoding | NONMMUT105770.1 |
| CUST_OEV3_024969 | 0.010201941 | 2.4377534 | up | noncoding | NONMMUT107313.1 |
| CUST_OEV3_059715 | 0.01842061 | 3.2694075 | down | noncoding | AK086450 |
| CUST_OEV3_012691 | 0.03557905 | 6.3819695 | up | noncoding | NONMMUT123931.1 |
| CUST_OEV3_058503 | 0.010398464 | 7.9215164 | up | noncoding | XR_401893 |
| CUST_OEV3_051871 | 0.01883436 | 2.3584573 | up | noncoding | AK145167 |
| CUST_OEV3_017301 | 0.02736274 | 3.0021431 | up | noncoding | NONMMUT117738.1 |
| A_51_P152444 | 0.008442464 | 7.5667634 | up | noncoding | NR_027978 |
| CUST_OEV3_031472 | 0.020369442 | 3.49783 | down | noncoding | NONMMUT098822.1 |
| CUST_OEV3_068878 | 0.030473575 | 5.971598 | down | noncoding | NONMMUT018358.2 |
| CUST_OEV3_040702 | 0.04041021 | 2.0232978 | down | noncoding | NONMMUT086863.1 |
| CUST_OEV3_041744 | 0.027834142 | 9.361619 | up | noncoding | NONMMUT085466.1 |
| CUST_OEV3_044378 | 0.029391317 | 5.554378 | up | noncoding | NONMMUT082031.1 |
| CUST_OEV3_035608 | 0.03292748 | 4.3491983 | down | noncoding | NONMMUT093587.1 |
| CUST_OEV3_051081 | 0.01103502 | 2.8745468 | up | noncoding | AK045177 |
| CUST_OEV3_009055 | 0.024085231 | 2.2070415 | down | noncoding | NONMMUT128789.1 |
| CUST_OEV3_057001 | 0.018110897 | 2.4891412 | up | noncoding | AK135102 |
| CUST_OEV3_039688 | 0.0114523 | 2.6795743 | up | noncoding | NONMMUT088192.1 |
| CUST_OEV3_020399 | 0.03799911 | 2.5371127 | up | noncoding | NONMMUT113621.1 |
| CUST_OEV3_030126 | 0.026846012 | 3.542624 | up | noncoding | NONMMUT100489.1 |
| CUST_OEV3_007031 | 0.035625644 | 2.087913 | up | noncoding | NONMMUT131267.1 |
| CUST_OEV3_049069 | 0.013160205 | 2.14731 | down | noncoding | BU611995 |
| CUST_OEV3_062540 | 0.031516068 | 5.8307643 | up | noncoding | AK047343 |
| CUST_OEV3_037177 | 0.03420465 | 2.1591415 | down | noncoding | NONMMUT091522.1 |
| A_66_P107959 | 0.006725211 | 2.3747945 | up | noncoding | NR_045341 |
| CUST_OEV3_057371 | 0.002215714 | 4.2093945 | up | noncoding | AK028006 |
| CUST_OEV3_000498 | 0.032606203 | 2.1632633 | down | noncoding | NovelTID_00005672 |
| CUST_OEV3_059590 | 0.014542846 | 5.2051973 | down | noncoding | AK034713 |
| CUST_OEV3_039233 | 0.041773677 | 2.486228 | up | noncoding | NONMMUT088772.1 |
| CUST_OEV3_016264 | 0.005806509 | 2.4983263 | down | noncoding | NONMMUT119164.1 |
| CUST_OEV3_067228 | 0.017392574 | 6.5482965 | up | noncoding | NONMMUT023195.2 |
| CUST_OEV3_050213 | 0.04751582 | 2.1218495 | up | noncoding | NONMMUT073662.2 |
| CUST_OEV3_005235 | 0.03537825 | 4.1385975 | down | noncoding | NONMMUT133616.1 |
| CUST_OEV3_047843 | 0.022747878 | 2.0343173 | up | noncoding | NONMMUT077574.1 |
| CUST_OEV3_068514 | 0.024830723 | 2.450891 | up | noncoding | AK141794 |
| CUST_OEV3_023912 | 0.007368723 | 2.9503682 | down | noncoding | NONMMUT108784.1 |
| CUST_OEV3_045326 | 0.027983464 | 2.1155164 | down | noncoding | NONMMUT080797.1 |
| CUST_OEV3_019950 | 0.021260507 | 2.657605 | down | noncoding | NR_040592 |
| CUST_OEV3_050776 | 0.016812801 | 3.127094 | up | noncoding | NONMMUT072188.2 |
| CUST_OEV3_001473 | 0.040625483 | 2.9595518 | up | noncoding | NovelTID_00002548 |
| CUST_OEV3_057652 | 0.008866211 | 2.013483 | down | noncoding | AK133143 |

| CUST_OEV3_003360 | 0.010152206 | 2.798665 | up | noncoding | NONMMUT136037.1 |
| --- | --- | --- | --- | --- | --- |
| CUST_OEV3_024218 | 0.032547086 | 2.7061703 | down | noncoding | NONMMUT108350.1 |
| CUST_OEV3_053187 | 0.036554407 | 2.2674086 | down | noncoding | XR_378874 |
| CUST_OEV3_066596 | 0.038543314 | 2.5725884 | down | noncoding | NONMMUT025002.2 |
| CUST_OEV3_000895 | 0.02921156 | 4.215781 | up | noncoding | NovelTID_00004381 |
| CUST_OEV3_035112 | 0.007352651 | 2.688175 | up | noncoding | NONMMUT094193.1 |
| CUST_OEV3_003587 | 0.003205637 | 2.3135939 | down | noncoding | NONMMUT135750.1 |
| CUST_OEV3_068532 | 0.005925852 | 4.6470075 | up | noncoding | AK040941 |
| CUST_OEV3_052182 | 0.009120932 | 2.6305385 | down | noncoding | AK087344 |
| CUST_OEV3_061837 | 0.024480922 | 2.1451747 | up | noncoding | ENSMUST00000181716 |
| CUST_OEV3_070411 | 0.002594595 | 5.8839836 | up | noncoding | AK030072 |
| CUST_OEV3_014881 | 0.001926472 | 2.2707448 | down | noncoding | NONMMUT121048.1 |
| CUST_OEV3_047924 | 0.006381545 | 2.5734942 | up | noncoding | NONMMUT077470.1 |
| CUST_OEV3_020647 | 0.024370369 | 2.2841747 | up | noncoding | NONMMUT113283.1 |
| CUST_OEV3_042463 | 0.003902545 | 4.1424375 | up | noncoding | NONMMUT084521.1 |
| CUST_OEV3_002009 | 0.006110934 | 7.3286357 | up | noncoding | NovelTID_00000541 |
| A_66_P111765 | 0.04110456 | 2.343748 | down | noncoding | NR_130985 |
| A_55_P2508173 | 0.021155966 | 2.1827679 | up | noncoding | NR_027866 |
| CUST_OEV3_066765 | 0.001570695 | 2.2310464 | down | noncoding | NONMMUT024540.2 |
| CUST_OEV3_018007 | 0.042774174 | 3.1759152 | down | noncoding | NONMMUT116854.1 |
| CUST_OEV3_090380 | 0.009835391 | 2.2300084 | down | noncoding | AK081239 |
| CUST_OEV3_038021 | 0.013118744 | 2.038291 | down | noncoding | NONMMUT090425.1 |
| CUST_OEV3_092333 | 0.017532429 | 3.1731837 | up | noncoding | CF104318 |
| CUST_OEV3_019597 | 0.04057781 | 2.6485064 | down | noncoding | NONMMUT114694.1 |
| CUST_OEV3_055785 | 0.016135 | 3.061882 | down | noncoding | NONMMUT056841.2 |
| CUST_OEV3_000248 | 0.012107421 | 2.582021 | up | noncoding | NovelTID_00006426 |
| CUST_OEV3_010896 | 0.021840852 | 2.0192409 | up | noncoding | NONMMUT126286.1 |
| CUST_OEV3_045891 | 0.02251921 | 2.1011982 | down | noncoding | NONMMUT080055.1 |
| CUST_OEV3_000443 | 0.047604512 | 3.6537595 | up | noncoding | NovelTID_00005850 |
| A_66_P102567 | 0.013718624 | 3.4241917 | up | noncoding | NR_045471 |
| CUST_OEV3_014272 | 0.03030615 | 2.0445266 | down | noncoding | NONMMUT121850.1 |
| CUST_OEV3_019160 | 0.034926206 | 6.7432036 | up | noncoding | NONMMUT115301.1 |
| CUST_OEV3_013684 | 0.048747536 | 4.241443 | up | noncoding | NONMMUT122637.1 |
| CUST_OEV3_046345 | 0.02107076 | 2.2938945 | up | noncoding | NONMMUT079460.1 |
| CUST_OEV3_005099 | 0.022933451 | 2.0918498 | down | noncoding | NONMMUT133798.1 |
| CUST_OEV3_066067 | 0.030367095 | 2.04953 | up | noncoding | NONMMUT026590.2 |
| CUST_OEV3_070123 | 0.002293537 | 4.7149577 | up | noncoding | ENSMUST00000152917 |
| CUST_OEV3_048083 | 0.018155921 | 2.743577 | down | noncoding | NONMMUT077247.1 |
| CUST_OEV3_026279 | 0.04028081 | 2.7150402 | down | noncoding | NONMMUT105566.1 |
| CUST_OEV3_011242 | 0.008683119 | 2.1441073 | down | noncoding | NONMMUT125839.1 |
| A_66_P115446 | 0.04184021 | 2.1804943 | up | noncoding | NR_026596 |
| CUST_OEV3_068698 | 0.023145476 | 2.18041 | down | noncoding | AK086330 |
| CUST_OEV3_055467 | 0.010112931 | 2.250451 | up | noncoding | NONMMUT057688.2 |
| CUST_OEV3_048007 | 0.043104183 | 2.406557 | up | noncoding | NONMMUT077355.1 |
| CUST_OEV3_034976 | 0.010216696 | 2.7879655 | down | noncoding | NONMMUT094374.1 |
| CUST_OEV3_054968 | 0.02567567 | 2.637879 | up | noncoding | NONMMUT059121.2 |
| CUST_OEV3_050731 | 0.011606563 | 2.1369326 | up | noncoding | AK157285 |
| CUST_OEV3_059662 | 0.001803403 | 2.094868 | up | noncoding | AK141653 |
| CUST_OEV3_073821 | 0.002916722 | 2.8313415 | down | noncoding | NONMMUT003633.2 |
| CUST_OEV3_054115 | 0.010232817 | 2.435337 | up | noncoding | NONMMUT062360.2 |
| CUST_OEV3_009998 | 0.0277104 | 3.040599 | down | noncoding | NONMMUT127545.1 |
| CUST_OEV3_033470 | 0.043738022 | 2.6959224 | down | noncoding | NONMMUT096270.1 |
| CUST_OEV3_028479 | 0.006620512 | 2.010469 | up | noncoding | NONMMUT102756.1 |
| CUST_OEV3_038397 | 0.029576246 | 2.568732 | down | noncoding | NONMMUT089897.1 |
| CUST_OEV3_052943 | 0.010074865 | 3.339282 | down | noncoding | AK080210 |
| A_51_P495730 | 0.03039784 | 2.033519 | up | noncoding | NR_003644 |
| CUST_OEV3_007893 | 0.04714376 | 2.568934 | up | noncoding | NONMMUT130235.1 |
| CUST_OEV3_029597 | 0.02431903 | 3.575588 | down | noncoding | NONMMUT101246.1 |
| CUST_OEV3_013699 | 0.020650396 | 3.5343945 | up | noncoding | NONMMUT122621.1 |
| CUST_OEV3_050898 | 0.036986277 | 2.1804879 | up | noncoding | NONMMUT071797.2 |
| CUST_OEV3_090016 | 0.03568832 | 2.275406 | up | noncoding | XR_387868 |
| A_55_P1975425 | 0.008436316 | 3.0720987 | up | noncoding | NR_028285 |
| CUST_OEV3_027300 | 0.016378203 | 2.348357 | down | noncoding | NONMMUT104230.1 |

| CUST_OEV3_056643 | 0.006268593 | 2.3219576 | up | noncoding | AK016534 |
| --- | --- | --- | --- | --- | --- |
| CUST_OEV3_072471 | 0.04592074 | 2.0338507 | down | noncoding | NONMMUT007531.2 |
| CUST_OEV3_047134 | 0.028209262 | 2.0621502 | down | noncoding | NONMMUT078479.1 |
| CUST_OEV3_046632 | 0.034489557 | 2.0345325 | down | noncoding | NONMMUT079081.1 |
| CUST_OEV3_001788 | 0.007560823 | 2.2158623 | down | noncoding | NovelTID_00001412 |
| CUST_OEV3_021613 | 0.043737624 | 4.672275 | down | noncoding | NONMMUT111936.1 |
| CUST_OEV3_044945 | 0.034198824 | 2.6162179 | down | noncoding | NONMMUT081328.1 |
| A_55_P2508748 | 0.011973392 | 4.6494837 | up | noncoding | NR_040443 |
| CUST_OEV3_015758 | 0.005348725 | 3.824226 | down | noncoding | NONMMUT119899.1 |
| CUST_OEV3_015617 | 0.025090959 | 2.5313077 | down | noncoding | NONMMUT120112.1 |
| CUST_OEV3_023933 | 0.034570914 | 2.1273925 | up | noncoding | NONMMUT108755.1 |
| CUST_OEV3_055753 | 0.026828982 | 5.045343 | up | noncoding | NONMMUT056927.2 |
| CUST_OEV3_042931 | 0.008694608 | 19.139421 | up | noncoding | NONMMUT083899.1 |
| CUST_OEV3_000647 | 0.047114454 | 3.6212099 | down | noncoding | NovelTID_00005213 |
| CUST_OEV3_025641 | 0.026153596 | 2.4216518 | down | noncoding | NONMMUT106427.1 |
| CUST_OEV3_015003 | 0.0334814 | 8.683268 | down | noncoding | NONMMUT120897.1 |
| CUST_OEV3_014920 | 0.01577348 | 2.3693688 | down | noncoding | NONMMUT120996.1 |
| CUST_OEV3_030769 | 0.011202214 | 4.7906804 | up | noncoding | NONMMUT099701.1 |
| CUST_OEV3_028285 | 0.030113796 | 2.3528745 | up | noncoding | AK140123 |
| CUST_OEV3_020460 | 0.001031627 | 4.912097 | up | noncoding | NONMMUT113537.1 |
| CUST_OEV3_010315 | 0.002626996 | 2.0935714 | down | noncoding | NONMMUT127130.1 |
| CUST_OEV3_092608 | 0.03992724 | 7.8946877 | up | noncoding | XR_400713 |
| A_55_P2246905 | 0.013490294 | 3.4924507 | up | noncoding | NR_040541 |
| CUST_OEV3_067651 | 0.027874375 | 2.063027 | down | noncoding | NONMMUT021962.2 |
| CUST_OEV3_048719 | 0.001743781 | 6.731553 | up | noncoding | NONMMUT076393.1 |
| CUST_OEV3_074970 | 0.02848776 | 2.37988 | up | noncoding | AK142999 |
| CUST_OEV3_016298 | 0.031941447 | 3.7596319 | up | noncoding | NONMMUT119099.1 |
| CUST_OEV3_065885 | 0.01735113 | 2.0778422 | up | noncoding | NONMMUT026980.2 |
| CUST_OEV3_040095 | 0.033832204 | 2.2423139 | down | noncoding | NONMMUT087649.1 |
| A_55_P2059120 | 0.03841727 | 2.209014 | down | noncoding | XR_388996 |
| CUST_OEV3_019150 | 0.001953991 | 18.821568 | up | noncoding | NONMMUT115312.1 |
| CUST_OEV3_039926 | 0.011017039 | 2.140908 | up | noncoding | NONMMUT087858.1 |
| CUST_OEV3_022165 | 0.045910574 | 2.3884425 | up | noncoding | NONMMUT111169.1 |
| CUST_OEV3_022126 | 0.006239584 | 3.172997 | up | noncoding | NONMMUT111219.1 |
| A_55_P2071779 | 0.019803898 | 2.698991 | up | noncoding | NR_027968 |
| CUST_OEV3_050589 | 0.033866707 | 2.7628129 | up | noncoding | NAP017530-001 |
| CUST_OEV3_007249 | 0.001006097 | 2.5177653 | down | noncoding | NONMMUT130975.1 |
| CUST_OEV3_038004 | 0.006472267 | 2.416673 | down | noncoding | NONMMUT090446.1 |
| CUST_OEV3_026260 | 0.01930198 | 2.3222914 | down | noncoding | NONMMUT105589.1 |
| CUST_OEV3_070246 | 0.009853705 | 3.4267685 | up | noncoding | NONMMUT014391.2 |
| CUST_OEV3_042872 | 0.002070479 | 7.4424405 | up | noncoding | NONMMUT083974.1 |
| CUST_OEV3_007410 | 0.015110694 | 3.0051265 | up | noncoding | NONMMUT130790.1 |
| CUST_OEV3_009800 | 0.003738046 | 4.109212 | up | noncoding | NONMMUT127825.1 |
| CUST_OEV3_018119 | 0.011409081 | 2.2886314 | down | noncoding | NR_033140 |
| CUST_OEV3_049051 | 0.046291374 | 2.5863214 | up | noncoding | NONMMUT075945.1 |
| CUST_OEV3_069966 | 0.038879983 | 2.436131 | down | noncoding | NONMMUT015068.2 |
| CUST_OEV3_012513 | 0.04500228 | 2.3010776 | down | noncoding | NONMMUT124167.1 |
| CUST_OEV3_069179 | 0.027757159 | 2.093745 | down | noncoding | XR_382311 |
| CUST_OEV3_072423 | 0.046155848 | 2.0631108 | up | noncoding | AK034590 |
| CUST_OEV3_043810 | 0.045464057 | 2.441884 | up | noncoding | NONMMUT082765.1 |
| CUST_OEV3_045453 | 0.026888352 | 2.67063 | down | noncoding | NONMMUT080635.1 |
| CUST_OEV3_015592 | 0.030769378 | 2.6143143 | up | noncoding | NONMMUT120142.1 |
| CUST_OEV3_019838 | 0.01442316 | 2.4805613 | down | noncoding | NONMMUT114375.1 |
| CUST_OEV3_042342 | 0.019151554 | 2.0052264 | down | noncoding | NONMMUT084667.1 |
| CUST_OEV3_039350 | 0.03682382 | 2.2473142 | down | noncoding | NONMMUT088619.1 |
| A_55_P2720550 | 1.94E-04 | 6.146787 | up | noncoding | NR_110521 |
| CUST_OEV3_030875 | 0.027910814 | 2.1265788 | up | noncoding | NONMMUT099564.1 |
| CUST_OEV3_069796 | 0.017789623 | 2.5387094 | up | noncoding | AK048792 |
| A_51_P286106 | 0.04729294 | 2.32865 | up | noncoding | XR_395125 |
| CUST_OEV3_048219 | 0.046226658 | 2.0463696 | down | noncoding | NONMMUT077066.1 |
| CUST_OEV3_041170 | 0.003997588 | 2.3573492 | up | noncoding | NONMMUT086250.1 |
| CUST_OEV3_010044 | 0.046931624 | 4.056318 | up | noncoding | TC1748245 |
| CUST_OEV3_007657 | 0.0337144 | 2.9058907 | down | noncoding | NONMMUT130511.1 |

| CUST_OEV3_019660 | 0.017829474 | 2.365029 | up | noncoding | AK041575 |
| --- | --- | --- | --- | --- | --- |
| A_66_P136149 | 0.028721226 | 2.4655483 | up | noncoding | XR_381794 |
| CUST_OEV3_033627 | 0.044220027 | 2.6782017 | down | noncoding | NONMMUT096071.1 |
| CUST_OEV3_057857 | 0.019665074 | 2.3049402 | up | noncoding | NR_040670 |
| A_55_P1957997 | 0.02937253 | 2.152143 | up | noncoding | XR_396976 |
| CUST_OEV3_048714 | 0.037630253 | 2.3865304 | up | noncoding | NONMMUT076399.1 |
| CUST_OEV3_042860 | 0.042016976 | 2.0607405 | down | noncoding | NONMMUT083998.1 |
| CUST_OEV3_066921 | 0.007575498 | 2.0210402 | up | noncoding | XR_384214 |
| CUST_OEV3_020316 | 0.040554486 | 2.3556333 | down | noncoding | NONMMUT113737.1 |
| CUST_OEV3_039872 | 0.025279416 | 2.3006911 | down | noncoding | NONMMUT087934.1 |
| CUST_OEV3_069383 | 0.038676925 | 4.2035675 | down | noncoding | AK037307 |
| CUST_OEV3_031389 | 0.003583706 | 2.5095303 | down | noncoding | NONMMUT098912.1 |
| CUST_OEV3_074589 | 0.024262652 | 2.8275335 | down | noncoding | AK142958 |
| CUST_OEV3_044260 | 0.006724234 | 10.045177 | up | noncoding | NONMMUT082183.1 |
| CUST_OEV3_074547 | 0.001727303 | 3.8566408 | up | noncoding | AK139661 |
| CUST_OEV3_056887 | 0.008739022 | 5.6229715 | up | noncoding | AK145714 |
| CUST_OEV3_093323 | 6.19E-04 | 5.5076466 | up | noncoding | ENSMUST00000157556.1 |
| CUST_OEV3_066132 | 3.62E-04 | 3.8521411 | up | noncoding | AK079782 |
| CUST_OEV3_053967 | 0.038575545 | 2.6544025 | down | noncoding | AK008330 |
| CUST_OEV3_072296 | 0.038331274 | 3.3619163 | down | noncoding | TC1660049 |
| CUST_OEV3_024285 | 0.009555478 | 2.060417 | down | noncoding | NONMMUT108231.1 |
| CUST_OEV3_044960 | 0.037856746 | 12.033582 | up | noncoding | NONMMUT081313.1 |
| CUST_OEV3_065208 | 0.006861895 | 2.4517498 | down | noncoding | NONMMUT028958.2 |
| A_55_P2849821 | 0.015659543 | 2.274544 | up | noncoding | XR_880067 |
| CUST_OEV3_013584 | 0.03031711 | 2.0837314 | down | noncoding | NONMMUT122770.1 |
| CUST_OEV3_072707 | 0.009901243 | 2.6582832 | up | noncoding | AK142771 |
| A_52_P91274 | 0.007925902 | 2.6147733 | down | noncoding | NR_045422 |
| CUST_OEV3_025155 | 0.016551746 | 3.5034072 | up | noncoding | NONMMUT107070.1 |
| CUST_OEV3_031105 | 0.04575342 | 2.9032953 | up | noncoding | NONMMUT099266.1 |
| CUST_OEV3_069766 | 0.047804322 | 3.1631515 | up | noncoding | NONMMUT015593.2 |
| A_55_P2205650 | 0.031432033 | 2.7381914 | up | noncoding | NR_037964 |
| A_66_P105712 | 0.020326352 | 2.3200312 | up | noncoding | XR_390362 |
| CUST_OEV3_038460 | 0.00961719 | 2.2288082 | down | noncoding | NONMMUT089815.1 |
| CUST_OEV3_059266 | 0.03987603 | 2.3613575 | up | noncoding | NAP028938-1 |
| CUST_OEV3_074456 | 0.04863521 | 3.3756053 | down | noncoding | AK142312 |
| CUST_OEV3_048674 | 0.031892486 | 2.5653622 | up | noncoding | NONMMUT076460.1 |
| A_66_P103670 | 0.006364245 | 2.2239022 | up | noncoding | XR_865484 |
| CUST_OEV3_074108 | 0.011158852 | 5.71155 | up | noncoding | XR_398864 |
| CUST_OEV3_062446 | 0.016977523 | 5.58082 | down | noncoding | NAP060815-1 |
| CUST_OEV3_003228 | 0.046101537 | 2.8421032 | down | noncoding | NONMMUT136235.1 |
| CUST_OEV3_055320 | 0.003112689 | 2.1389213 | up | noncoding | AK031178 |
| CUST_OEV3_028117 | 4.75E-04 | 3.3102674 | up | noncoding | NONMMUT103212.1 |
| CUST_OEV3_022974 | 0.034756977 | 6.659116 | up | noncoding | NONMMUT110013.1 |
| CUST_OEV3_037943 | 0.028072713 | 2.5882378 | down | noncoding | NONMMUT090521.1 |
| CUST_OEV3_007061 | 0.009153477 | 2.628195 | up | noncoding | NONMMUT131233.1 |
| CUST_OEV3_072183 | 0.045190033 | 2.0866253 | down | noncoding | AK045003 |
| CUST_OEV3_045533 | 0.018701702 | 2.6510787 | down | noncoding | AK047977 |
| CUST_OEV3_015276 | 0.036641546 | 2.2159274 | up | noncoding | NONMMUT120543.1 |
| CUST_OEV3_043815 | 0.045445316 | 2.02234 | down | noncoding | NONMMUT082758.1 |
| CUST_OEV3_068097 | 0.03217534 | 3.4869103 | down | noncoding | BC028264 |
| CUST_OEV3_010116 | 5.10E-04 | 2.1022513 | up | noncoding | NONMMUT127377.1 |
| A_66_P120860 | 0.02225181 | 2.4099631 | up | noncoding | NR_040360 |
| CUST_OEV3_069307 | 0.003234771 | 4.0541954 | down | noncoding | NR_105041 |
| CUST_OEV3_029662 | 0.011737631 | 10.013858 | up | noncoding | NONMMUT101154.1 |
| CUST_OEV3_071604 | 0.049686447 | 2.7595758 | up | noncoding | AK153668 |
| CUST_OEV3_072399 | 0.019290298 | 2.0549815 | up | noncoding | AK015055 |
| CUST_OEV3_060939 | 0.016163664 | 2.2683759 | up | noncoding | XR_375245 |
| A_55_P2350067 | 0.015542476 | 3.8021288 | up | noncoding | NR_045765 |
| CUST_OEV3_031064 | 0.005838419 | 5.460647 | up | noncoding | NONMMUT099316.1 |
| CUST_OEV3_015886 | 0.02746406 | 3.5002604 | down | noncoding | NONMMUT119703.1 |
| CUST_OEV3_092427 | 0.04487081 | 3.0389278 | down | noncoding | ENSMUST00000198572.4 |
| CUST_OEV3_061228 | 0.00758072 | 4.213929 | up | noncoding | NAP014532-001 |
| CUST_OEV3_048422 | 0.0364214 | 2.1468146 | down | noncoding | NONMMUT076811.1 |

| CUST_OEV3_007472 | 0.006070009 | 2.6276639 | up | noncoding | NONMMUT130724.1 |
| --- | --- | --- | --- | --- | --- |
| CUST_OEV3_047958 | 0.017715134 | 2.172793 | up | noncoding | NONMMUT077418.1 |
| CUST_OEV3_068322 | 0.0184716 | 2.7340028 | up | noncoding | NONMMUT020049.2 |
| CUST_OEV3_057879 | 0.014671306 | 3.1254053 | up | noncoding | NONMMUT050768.2 |
| CUST_OEV3_037411 | 0.04612251 | 2.4007227 | down | noncoding | NONMMUT091226.1 |
| CUST_OEV3_045664 | 0.04187263 | 3.4083815 | down | noncoding | NONMMUT080339.1 |
| CUST_OEV3_000141 | 0.011079386 | 3.0524385 | up | noncoding | NovelTID_00006715 |
| CUST_OEV3_023480 | 0.004006851 | 4.0381007 | up | noncoding | NONMMUT109358.1 |
| CUST_OEV3_045867 | 0.04924952 | 5.9737473 | up | noncoding | NONMMUT080090.1 |
| CUST_OEV3_004277 | 0.013213978 | 2.3879108 | up | noncoding | NONMMUT134854.1 |
| CUST_OEV3_028581 | 0.022072623 | 2.1519217 | down | noncoding | NONMMUT102597.1 |
| A_55_P2509653 | 0.047742233 | 2.6808798 | up | noncoding | NR_027900 |
| CUST_OEV3_062834 | 0.02614904 | 2.1003206 | up | noncoding | NONMMUT035773.2 |
| A_55_P2094524 | 1.89E-04 | 6.9102035 | up | noncoding | NR_028590 |
| CUST_OEV3_092234 | 0.005315441 | 6.3934336 | up | noncoding | ENSMUST00000203666.1 |
| CUST_OEV3_040356 | 0.010923541 | 2.6719463 | up | noncoding | NONMMUT087309.1 |
| CUST_OEV3_047880 | 0.014811929 | 2.1317499 | down | noncoding | NONMMUT077527.1 |
| CUST_OEV3_059255 | 0.01468607 | 2.3574169 | up | noncoding | S89400 |
| CUST_OEV3_052961 | 0.023554033 | 2.1262681 | up | noncoding | NAP063013-1 |
| CUST_OEV3_058303 | 0.03549835 | 2.5077543 | up | noncoding | AK085229 |
| CUST_OEV3_020612 | 0.014092179 | 3.4603646 | up | noncoding | NONMMUT113332.1 |
| CUST_OEV3_025795 | 0.03464501 | 2.366234 | up | noncoding | XR_398406 |
| A_55_P2508843 | 0.015566509 | 2.0566127 | up | noncoding | NR_040680 |
| CUST_OEV3_026072 | 0.04985943 | 3.9762878 | up | noncoding | NONMMUT105856.1 |
| CUST_OEV3_000314 | 0.029339338 | 2.2907557 | down | noncoding | NovelTID_00006201 |
| CUST_OEV3_028477 | 0.012954739 | 2.5768895 | up | noncoding | NONMMUT102759.1 |
| CUST_OEV3_072313 | 0.011101179 | 2.8695774 | up | noncoding | NONMMUT008193.2 |
| CUST_OEV3_001400 | 0.002788463 | 3.855528 | up | noncoding | NovelTID_00002740 |
| CUST_OEV3_028466 | 0.01680773 | 2.2669892 | up | noncoding | NONMMUT102775.1 |
| CUST_OEV3_016334 | 0.003682262 | 11.967947 | up | noncoding | XR_389476 |
| A_55_P2786892 | 0.042886794 | 2.4755704 | up | noncoding | XR_380624 |
| CUST_OEV3_012555 | 0.035904966 | 2.1885688 | down | noncoding | NONMMUT124110.1 |
| CUST_OEV3_029310 | 0.030518977 | 2.095769 | up | noncoding | NONMMUT101607.1 |
| CUST_OEV3_018438 | 0.028826516 | 2.0814636 | up | noncoding | NONMMUT116265.1 |
| A_66_P133419 | 0.011752758 | 5.3826623 | up | noncoding | NR_045883 |
| CUST_OEV3_020377 | 0.03682623 | 2.389371 | up | noncoding | NONMMUT113652.1 |
| CUST_OEV3_048125 | 0.001177015 | 4.1104116 | up | noncoding | NONMMUT077190.1 |
| CUST_OEV3_054851 | 0.044179037 | 4.1011715 | down | noncoding | AK047646 |
| A_55_P2768796 | 0.039518826 | 2.2068553 | up | noncoding | XR_384211 |
| A_66_P109073 | 0.043040913 | 2.0731857 | up | noncoding | XR_863488 |
| CUST_OEV3_073154 | 0.030760922 | 6.2831035 | up | noncoding | XR_380060 |
| CUST_OEV3_032844 | 0.03195919 | 2.1741564 | down | noncoding | NONMMUT097089.1 |
| CUST_OEV3_001360 | 0.009450505 | 2.636046 | down | noncoding | NovelTID_00002865 |
| CUST_OEV3_015166 | 0.039864235 | 2.1157968 | down | noncoding | NONMMUT120687.1 |
| CUST_OEV3_064431 | 0.016426818 | 3.1475341 | down | noncoding | AK045230 |
| CUST_OEV3_025015 | 0.011912085 | 2.153593 | down | noncoding | NONMMUT107252.1 |
| CUST_OEV3_035377 | 0.037787434 | 2.1606002 | down | noncoding | NONMMUT093869.1 |
| CUST_OEV3_061387 | 0.017982962 | 3.8351421 | up | noncoding | NONMMUT039866.2 |
| CUST_OEV3_047722 | 0.04870083 | 2.9584851 | up | noncoding | AV585791 |
| A_66_P100114 | 0.005233323 | 4.6404767 | up | noncoding | NR_040552 |
| CUST_OEV3_017950 | 0.022404185 | 2.369824 | down | noncoding | NONMMUT116929.1 |
| CUST_OEV3_074045 | 0.021657676 | 2.452015 | up | noncoding | BC116196 |
| CUST_OEV3_041925 | 0.020961298 | 2.2798924 | down | noncoding | NONMMUT085202.1 |
| CUST_OEV3_005633 | 0.004381537 | 3.0813124 | down | noncoding | NONMMUT133096.1 |
| A_55_P2318955 | 0.011756376 | 3.084854 | up | noncoding | XR_886123 |
| CUST_OEV3_032343 | 0.021614704 | 5.361322 | up | noncoding | NONMMUT097731.1 |
| CUST_OEV3_054515 | 0.004110713 | 2.7377 | down | noncoding | NONMMUT060746.2 |
| CUST_OEV3_007982 | 0.020773849 | 10.352561 | up | noncoding | NONMMUT130134.1 |
| CUST_OEV3_054821 | 0.03204231 | 4.2618246 | up | noncoding | AK138546 |
| CUST_OEV3_041928 | 0.01049969 | 9.773382 | down | noncoding | NONMMUT085198.1 |
| CUST_OEV3_058161 | 0.015309905 | 15.903453 | up | noncoding | XR_390187 |
| CUST_OEV3_062812 | 0.002298168 | 2.5840402 | up | noncoding | NR_038162 |
| CUST_OEV3_022144 | 0.025411636 | 5.585475 | up | noncoding | NONMMUT111198.1 |

| CUST_OEV3_025935 | 0.003044121 | 6.2008843 | up | noncoding | NONMMUT106044.1 |
| --- | --- | --- | --- | --- | --- |
| CUST_OEV3_050402 | 0.028830253 | 2.0984318 | up | noncoding | NONMMUT073170.2 |
| CUST_OEV3_073104 | 0.012327877 | 2.5323846 | down | noncoding | NONMMUT005693.2 |
| CUST_OEV3_023044 | 1.95E-04 | 9.00508 | up | noncoding | NONMMUT109926.1 |
| CUST_OEV3_061236 | 0.028080313 | 2.4098332 | down | noncoding | AK045170 |
| CUST_OEV3_009154 | 0.008338608 | 3.2323813 | up | noncoding | NONMMUT128649.1 |
| CUST_OEV3_002818 | 0.029626925 | 2.2148604 | up | noncoding | NONMMUT136785.1 |
| CUST_OEV3_008941 | 0.04954507 | 2.2864208 | down | noncoding | NONMMUT128960.1 |
| CUST_OEV3_039727 | 0.0172323 | 4.3434343 | up | noncoding | NONMMUT088143.1 |
| CUST_OEV3_029633 | 0.013549927 | 2.2963731 | up | noncoding | NONMMUT101197.1 |
| CUST_OEV3_030621 | 0.0330183 | 3.0437381 | up | noncoding | NONMMUT099900.1 |
| A_65_P11034 | 0.005274423 | 3.0200582 | up | noncoding | NR_110348 |
| CUST_OEV3_010232 | 0.008079317 | 2.3334084 | down | noncoding | NONMMUT127232.1 |
| CUST_OEV3_092201 | 0.04615078 | 2.0642533 | up | noncoding | XR_377933 |
| CUST_OEV3_054948 | 0.005929574 | 3.940056 | up | noncoding | AK048124 |
| CUST_OEV3_000226 | 0.031430773 | 2.9310262 | up | noncoding | BE647616 |
| CUST_OEV3_032885 | 0.007329157 | 2.6189902 | down | noncoding | NONMMUT097033.1 |
| CUST_OEV3_033534 | 0.030328512 | 2.121123 | up | noncoding | NONMMUT096193.1 |
| CUST_OEV3_003846 | 0.047110047 | 3.4703143 | up | noncoding | NONMMUT135427.1 |
| CUST_OEV3_050719 | 0.023679435 | 2.041854 | up | noncoding | AK080319 |
| CUST_OEV3_016841 | 0.010198971 | 3.5222697 | up | noncoding | NONMMUT118355.1 |
| CUST_OEV3_031894 | 0.007505284 | 3.3709679 | up | noncoding | NONMMUT098317.1 |
| CUST_OEV3_012557 | 0.0228964 | 2.6212058 | down | noncoding | NONMMUT124108.1 |
| A_55_P2830254 | 0.035307422 | 2.2148986 | down | noncoding | NR_045781 |
| CUST_OEV3_026670 | 0.011987846 | 2.4354804 | up | noncoding | NONMMUT105044.1 |
| CUST_OEV3_058504 | 0.019597592 | 2.7733145 | up | noncoding | NONMMUT048532.2 |
| CUST_OEV3_027863 | 0.005089046 | 3.0124087 | down | noncoding | NONMMUT103529.1 |
| CUST_OEV3_065303 | 0.006524963 | 3.4600813 | down | noncoding | AK039165 |
| CUST_OEV3_005163 | 0.023739194 | 2.0406084 | down | noncoding | NONMMUT133706.1 |
| CUST_OEV3_091432 | 2.57E-04 | 2.0350544 | up | noncoding | AK043632 |
| CUST_OEV3_033810 | 0.02742909 | 2.0452404 | up | noncoding | NONMMUT095832.1 |
| CUST_OEV3_029850 | 0.004098519 | 2.0437996 | up | noncoding | NONMMUT100893.1 |
| CUST_OEV3_003472 | 0.015452448 | 3.4575586 | up | noncoding | NONMMUT135903.1 |
| CUST_OEV3_056762 | 0.010822689 | 2.144232 | up | noncoding | NONMMUT054050.2 |
| CUST_OEV3_066659 | 0.027636329 | 5.3794537 | up | noncoding | AK029390 |
| CUST_OEV3_012281 | 0.039999004 | 4.311083 | up | noncoding | NONMMUT124459.1 |
| CUST_OEV3_000247 | 0.032793757 | 5.4580007 | up | noncoding | NovelTID_00006427 |
| CUST_OEV3_056051 | 0.03247163 | 2.4381592 | up | noncoding | NONMMUT056144.2 |
| CUST_OEV3_009030 | 0.01783421 | 2.8441565 | up | noncoding | NONMMUT128823.1 |
| CUST_OEV3_045977 | 0.007290273 | 2.5022285 | down | noncoding | NONMMUT079947.1 |
| CUST_OEV3_048300 | 0.016077243 | 2.556444 | down | noncoding | NONMMUT076961.1 |
| CUST_OEV3_040784 | 0.01250514 | 2.1160634 | up | noncoding | NONMMUT086751.1 |
| CUST_OEV3_006274 | 0.001242192 | 2.0652 | up | noncoding | AK087878 |
| CUST_OEV3_011500 | 0.008172363 | 2.6201954 | up | noncoding | NONMMUT125553.1 |
| CUST_OEV3_003654 | 0.048740137 | 2.134096 | down | noncoding | NONMMUT135649.1 |
| A_55_P2578737 | 0.038043074 | 2.3956418 | up | noncoding | XR_389078 |
| CUST_OEV3_044925 | 0.043532234 | 3.9303546 | up | noncoding | NONMMUT081349.1 |
| CUST_OEV3_017635 | 0.026580386 | 2.39694 | up | noncoding | NONMMUT117349.1 |
| CUST_OEV3_063239 | 0.023682367 | 2.55122 | up | noncoding | NONMMUT034640.2 |
| CUST_OEV3_027260 | 5.76E-04 | 2.5636768 | up | noncoding | NONMMUT104279.1 |
| CUST_OEV3_069297 | 0.042732134 | 3.1001818 | up | noncoding | NONMMUT016995.2 |
| CUST_OEV3_059158 | 0.01706453 | 2.78052 | up | noncoding | NONMMUT046776.2 |
| CUST_OEV3_069454 | 0.015947264 | 2.4175084 | up | noncoding | BF714904 |
| CUST_OEV3_017365 | 0.012725076 | 2.15045 | up | noncoding | NONMMUT117668.1 |
| CUST_OEV3_002625 | 0.019696899 | 2.0378962 | down | noncoding | NONMMUT137067.1 |
| CUST_OEV3_072620 | 0.008560684 | 9.549438 | up | noncoding | AK076627 |
| CUST_OEV3_037540 | 0.03258498 | 2.1198153 | down | noncoding | NONMMUT091055.1 |
| CUST_OEV3_055066 | 0.016253179 | 2.166393 | up | noncoding | AK041190 |
| CUST_OEV3_053030 | 0.018646125 | 2.2436457 | down | noncoding | AK138239 |
| CUST_OEV3_067832 | 0.044173505 | 3.3308127 | up | noncoding | XR_395981 |
| CUST_OEV3_048010 | 0.025635963 | 2.2785478 | up | noncoding | NONMMUT077352.1 |
| CUST_OEV3_071953 | 0.02768596 | 4.0771194 | up | noncoding | XR_381138 |
| CUST_OEV3_040815 | 0.006664848 | 6.4156475 | up | noncoding | NONMMUT086718.1 |

| CUST_OEV3_030734 | 0.030924575 | 7.390796 | up | noncoding | NONMMUT099752.1 |
| --- | --- | --- | --- | --- | --- |
| CUST_OEV3_045795 | 0.022815954 | 2.196038 | up | noncoding | NONMMUT080175.1 |
| CUST_OEV3_034751 | 0.028490584 | 2.6896238 | up | noncoding | XR_396018 |
| CUST_OEV3_012601 | 0.024569087 | 2.0161753 | up | noncoding | NONMMUT124050.1 |
| CUST_OEV3_007131 | 0.008755132 | 2.9592113 | up | noncoding | ENSMUST00000178529 |
| CUST_OEV3_021284 | 0.011530357 | 2.2874486 | up | noncoding | NONMMUT112395.1 |
| A_51_P315202 | 0.041450504 | 7.9451838 | down | noncoding | XR_863618 |
| CUST_OEV3_052322 | 0.0104008 | 2.2951984 | up | noncoding | XR_388151 |
| CUST_OEV3_051828 | 0.048326224 | 2.5382268 | up | noncoding | AK028456 |
| CUST_OEV3_068180 | 0.035939373 | 2.3884552 | down | noncoding | AK163819 |
| CUST_OEV3_050689 | 0.016580202 | 2.8303685 | up | noncoding | NAP027194-1 |
| CUST_OEV3_044934 | 0.022332732 | 2.396467 | up | noncoding | NONMMUT081339.1 |
| CUST_OEV3_054621 | 0.047854923 | 2.2226162 | down | noncoding | NAP069167-1 |
| CUST_OEV3_008605 | 0.034524124 | 3.686282 | up | noncoding | NONMMUT129382.1 |
| CUST_OEV3_017238 | 0.04389723 | 2.274929 | up | noncoding | NONMMUT117817.1 |
| CUST_OEV3_049487 | 0.002906602 | 2.2735665 | down | noncoding | NONMMUT075385.1 |
| CUST_OEV3_007040 | 0.027321901 | 4.342086 | up | noncoding | NONMMUT131255.1 |
| CUST_OEV3_024407 | 0.030497346 | 2.262205 | up | noncoding | NONMMUT108064.1 |
| CUST_OEV3_052787 | 0.038070638 | 2.8157883 | down | noncoding | AK050936 |
| CUST_OEV3_054128 | 0.04208084 | 2.907303 | down | noncoding | TC1689630 |
| CUST_OEV3_067994 | 0.024658771 | 3.1453586 | up | noncoding | NONMMUT021072.2 |
| CUST_OEV3_026218 | 0.020045588 | 4.8417096 | up | noncoding | NONMMUT105652.1 |
| CUST_OEV3_001244 | 0.022282785 | 2.5284755 | down | noncoding | NovelTID_00003330 |
| CUST_OEV3_044593 | 0.007815971 | 2.0521488 | up | noncoding | NONMMUT081745.1 |
| CUST_OEV3_072051 | 0.045204528 | 2.3619194 | up | noncoding | AK052949 |
| CUST_OEV3_043300 | 0.013099363 | 2.087428 | down | noncoding | NONMMUT083404.1 |
| CUST_OEV3_092462 | 0.02717624 | 2.4962409 | up | noncoding | BY721406 |
| A_55_P2509814 | 0.025412185 | 2.616766 | up | noncoding | NR_040651 |
| CUST_OEV3_045221 | 0.002550175 | 2.260333 | down | noncoding | NONMMUT080933.1 |
| CUST_OEV3_028735 | 0.039421566 | 2.5931048 | down | noncoding | NONMMUT102376.1 |
| CUST_OEV3_004385 | 0.004255504 | 2.0057712 | up | noncoding | NONMMUT134698.1 |
| CUST_OEV3_045589 | 0.001599263 | 2.1388066 | up | noncoding | XR_380584 |
| CUST_OEV3_020402 | 0.003722525 | 3.1207843 | down | noncoding | NONMMUT113616.1 |
| CUST_OEV3_092936 | 0.04933619 | 3.5703123 | up | noncoding | ENSMUST00000184714.1 |
| CUST_OEV3_014221 | 0.004932439 | 2.0096505 | up | noncoding | NONMMUT121908.1 |
| CUST_OEV3_008085 | 0.007282976 | 3.283977 | down | noncoding | NONMMUT130006.1 |
| CUST_OEV3_045725 | 0.032682966 | 2.588086 | down | noncoding | NONMMUT080261.1 |
| CUST_OEV3_090636 | 0.012288712 | 2.3379662 | up | noncoding | AK046352 |
| CUST_OEV3_070815 | 0.006872971 | 2.074922 | up | noncoding | NONMMUT012578.2 |
| CUST_OEV3_014723 | 0.025947144 | 2.601523 | down | noncoding | NONMMUT121247.1 |
| CUST_OEV3_040173 | 0.035307705 | 2.3756456 | down | noncoding | NONMMUT087549.1 |
| CUST_OEV3_016079 | 0.023058372 | 2.3296933 | up | noncoding | TC1643920 |
| CUST_OEV3_047871 | 0.022027357 | 2.3249583 | down | noncoding | CO810138 |
| A_66_P117241 | 0.02186562 | 4.7625594 | up | noncoding | NR_102303 |
| CUST_OEV3_034923 | 0.004750752 | 8.703452 | up | noncoding | NONMMUT094450.1 |
| CUST_OEV3_023568 | 0.00390269 | 4.0322585 | up | noncoding | NONMMUT109252.1 |
| CUST_OEV3_026771 | 0.03199138 | 2.9319181 | down | noncoding | NONMMUT104909.1 |
| A_55_P2509543 | 0.001506032 | 2.3707306 | up | noncoding | NR_028591 |
| CUST_OEV3_000256 | 0.009394063 | 2.3894825 | up | noncoding | NovelTID_00006402 |
| CUST_OEV3_019732 | 0.011361888 | 2.3805125 | up | noncoding | NONMMUT114499.1 |
| A_55_P2508728 | 0.028852634 | 3.718095 | up | noncoding | NR_040412 |
| CUST_OEV3_059780 | 0.007787847 | 2.337101 | up | noncoding | NONMMUT045093.2 |
| CUST_OEV3_009799 | 0.016285487 | 2.4465072 | down | noncoding | NONMMUT127827.1 |
| CUST_OEV3_020912 | 0.016515002 | 2.3673863 | down | noncoding | NONMMUT112916.1 |
| CUST_OEV3_049580 | 8.11E-04 | 2.639109 | down | noncoding | NONMMUT075270.1 |
| CUST_OEV3_069002 | 0.006536634 | 5.698537 | up | noncoding | NR_028430 |
| CUST_OEV3_047876 | 0.02759978 | 3.255307 | down | noncoding | NONMMUT077531.1 |
| CUST_OEV3_040128 | 0.010230817 | 2.2583866 | up | noncoding | NONMMUT087607.1 |
| CUST_OEV3_032505 | 0.03823253 | 2.2171109 | up | noncoding | NONMMUT097511.1 |
| CUST_OEV3_065960 | 0.033505633 | 2.02533 | up | noncoding | NONMMUT026793.2 |
| CUST_OEV3_051573 | 0.028205564 | 2.6551783 | down | noncoding | AK085354 |
| CUST_OEV3_014872 | 0.03636506 | 2.714878 | down | noncoding | NONMMUT121061.1 |
| CUST_OEV3_064401 | 0.029459346 | 2.662576 | down | noncoding | AK028973 |

| CUST_OEV3_005801 | 0.008564468 | 2.273703 | up | noncoding | AK021347 |
| --- | --- | --- | --- | --- | --- |
| CUST_OEV3_063966 | 0.03222959 | 3.9744077 | up | noncoding | AK155291 |
| CUST_OEV3_025919 | 0.016291115 | 6.823246 | up | noncoding | NONMMUT106065.1 |
| CUST_OEV3_031299 | 0.023835754 | 2.2948928 | down | noncoding | NONMMUT099010.1 |
| CUST_OEV3_001308 | 0.025725033 | 3.1759737 | up | noncoding | NovelTID_00003013 |
| CUST_OEV3_022557 | 0.04566689 | 2.6585774 | down | noncoding | NONMMUT110629.1 |
| CUST_OEV3_073304 | 0.004842782 | 2.7387538 | down | noncoding | AK030486 |
| CUST_OEV3_032757 | 0.046970103 | 2.766522 | down | noncoding | NONMMUT097198.1 |
| CUST_OEV3_027138 | 0.015214077 | 5.1990147 | up | noncoding | NONMMUT104438.1 |
| CUST_OEV3_063509 | 0.019125288 | 2.195752 | up | noncoding | NONMMUT033990.2 |
| CUST_OEV3_013748 | 0.040103298 | 2.0054185 | down | noncoding | NONMMUT122565.1 |
| CUST_OEV3_020088 | 0.016612113 | 2.5086098 | down | noncoding | NONMMUT114039.1 |
| CUST_OEV3_054048 | 0.04086309 | 3.0825474 | up | noncoding | NONMMUT062575.2 |
| CUST_OEV3_013199 | 0.024128426 | 2.2368617 | down | noncoding | NONMMUT123265.1 |
| CUST_OEV3_002335 | 0.027382195 | 2.2864683 | down | noncoding | NONMMUT137468.1 |
| CUST_OEV3_005680 | 0.044837333 | 2.3464255 | up | noncoding | NONMMUT133038.1 |
| CUST_OEV3_020199 | 0.010210255 | 2.7159116 | up | noncoding | NONMMUT113906.1 |
| A_66_P102538 | 0.027705504 | 2.1075785 | up | noncoding | NR_110487 |
| CUST_OEV3_055195 | 0.012130393 | 3.7046213 | up | noncoding | AK134844 |
| CUST_OEV3_054706 | 0.034435835 | 3.387667 | up | noncoding | NONMMUT059942.2 |
| CUST_OEV3_048497 | 0.00881544 | 2.2057288 | down | noncoding | NONMMUT076704.1 |
| CUST_OEV3_035774 | 0.003236838 | 4.640144 | up | noncoding | NONMMUT093377.1 |
| CUST_OEV3_039992 | 0.042894468 | 2.8822956 | up | noncoding | NONMMUT087778.1 |
| A_66_P101942 | 0.04326106 | 3.2969115 | down | noncoding | XR_168557 |
| CUST_OEV3_089948 | 0.03286837 | 2.0933666 | up | noncoding | AK053200 |
| CUST_OEV3_093806 | 0.030031629 | 2.017149 | up | noncoding | NR_027290 |
| CUST_OEV3_028913 | 0.037669152 | 2.7567563 | down | noncoding | NONMMUT102144.1 |
| CUST_OEV3_052857 | 0.047820378 | 3.239874 | up | noncoding | AK142664 |
| A_55_P2574200 | 0.021361914 | 2.0459814 | up | noncoding | XR_391642 |
| CUST_OEV3_022798 | 0.04161516 | 2.2491877 | up | noncoding | NONMMUT110259.1 |
| CUST_OEV3_072929 | 0.012221074 | 2.1120868 | up | noncoding | AK084334 |
| CUST_OEV3_039984 | 0.02108637 | 2.7211595 | down | noncoding | NONMMUT087788.1 |
| CUST_OEV3_042588 | 0.035087377 | 5.123365 | up | noncoding | NONMMUT084356.1 |
| CUST_OEV3_035444 | 0.044005826 | 2.638763 | down | noncoding | NONMMUT093785.1 |
| CUST_OEV3_092932 | 0.020403767 | 6.9289804 | up | noncoding | ENSMUST00000184835.1 |
| CUST_OEV3_004424 | 0.020220568 | 2.0428905 | up | noncoding | NONMMUT134642.1 |
| CUST_OEV3_032542 | 1.03E-04 | 4.2963266 | up | noncoding | NONMMUT097464.1 |
| CUST_OEV3_046332 | 0.012241643 | 3.7939868 | down | noncoding | NONMMUT079477.1 |
| CUST_OEV3_024165 | 0.02402278 | 2.5166786 | down | noncoding | NONMMUT108418.1 |
| CUST_OEV3_068968 | 0.003826095 | 17.366116 | up | noncoding | NONMMUT017995.2 |
| CUST_OEV3_001819 | 0.016818013 | 2.3194623 | down | noncoding | NovelTID_00001323 |
| CUST_OEV3_047182 | 0.037350684 | 2.1456528 | up | noncoding | NONMMUT078411.1 |
| CUST_OEV3_070255 | 0.034555465 | 2.3488538 | up | noncoding | AK164851 |
| CUST_OEV3_018472 | 0.002662814 | 4.092419 | up | noncoding | NONMMUT116221.1 |
| CUST_OEV3_038880 | 0.03709229 | 2.134341 | down | noncoding | NONMMUT089245.1 |
| CUST_OEV3_043298 | 0.006741518 | 2.3922403 | down | noncoding | NONMMUT083406.1 |
| CUST_OEV3_030053 | 0.026466621 | 2.5327702 | down | noncoding | NONMMUT100592.1 |
| CUST_OEV3_060317 | 0.002233454 | 2.0534866 | up | noncoding | AK047052 |
| CUST_OEV3_007075 | 0.010475551 | 3.249067 | up | noncoding | NONMMUT131216.1 |
| CUST_OEV3_020625 | 0.010817555 | 7.3613486 | up | noncoding | NONMMUT113315.1 |
| CUST_OEV3_023903 | 0.04264726 | 2.2281961 | down | noncoding | NONMMUT108797.1 |
| CUST_OEV3_069606 | 0.007075877 | 5.2665467 | up | noncoding | NONMMUT016154.2 |
| CUST_OEV3_070115 | 0.022241227 | 3.6188533 | up | noncoding | AK083182 |
| CUST_OEV3_059000 | 0.045064032 | 2.374779 | down | noncoding | AK030927 |
| CUST_OEV3_066818 | 0.002472583 | 2.6760864 | down | noncoding | AK021252 |
| CUST_OEV3_016270 | 0.044281002 | 2.821465 | down | noncoding | NONMMUT119146.1 |
| CUST_OEV3_052383 | 0.00886805 | 11.233333 | up | noncoding | NONMMUT067383.2 |
| CUST_OEV3_034318 | 0.028285976 | 2.3207943 | up | noncoding | NONMMUT095213.1 |
| CUST_OEV3_052777 | 0.014555006 | 2.1473114 | up | noncoding | NONMMUT066303.2 |
| CUST_OEV3_008615 | 0.008409541 | 6.327637 | up | noncoding | NONMMUT129372.1 |
| CUST_OEV3_013688 | 0.03116413 | 2.3061292 | down | noncoding | NONMMUT122633.1 |
| CUST_OEV3_032198 | 0.040158488 | 2.3557944 | up | noncoding | NONMMUT097905.1 |
| CUST_OEV3_040922 | 0.00544089 | 2.2991185 | up | noncoding | NONMMUT086590.1 |

| CUST_OEV3_073759 | 0.011259659 | 2.0013027 | up | noncoding | AK020987 |
| --- | --- | --- | --- | --- | --- |
| CUST_OEV3_090450 | 0.02787816 | 3.6585407 | up | noncoding | NAP051793-1 |
| CUST_OEV3_033863 | 0.004670056 | 2.4783583 | up | noncoding | NONMMUT095766.1 |
| CUST_OEV3_015641 | 7.13E-05 | 2.1429582 | up | noncoding | NONMMUT120079.1 |
| CUST_OEV3_064922 | 0.045096952 | 2.017952 | up | noncoding | AK084424 |
| CUST_OEV3_090289 | 0.035367515 | 3.322498 | down | noncoding | AK030457 |
| CUST_OEV3_052833 | 0.02988737 | 2.216098 | down | noncoding | AK038373 |
| CUST_OEV3_012356 | 0.014801955 | 2.1689098 | up | noncoding | NONMMUT124360.1 |
| CUST_OEV3_030704 | 0.012611447 | 2.2124772 | down | noncoding | NONMMUT099797.1 |
| CUST_OEV3_074827 | 0.015823811 | 2.3872838 | up | noncoding | AK046227 |
| CUST_OEV3_071758 | 0.015272479 | 2.5727644 | down | noncoding | AK043190 |
| CUST_OEV3_053673 | 0.011085967 | 2.0662928 | up | noncoding | AK084155 |
| CUST_OEV3_026192 | 0.006412833 | 2.274923 | down | noncoding | NONMMUT105685.1 |
| CUST_OEV3_035276 | 0.02162658 | 4.586066 | up | noncoding | NONMMUT093991.1 |
| CUST_OEV3_040297 | 0.029650994 | 2.3997374 | up | noncoding | NONMMUT087376.1 |
| CUST_OEV3_015797 | 0.005733025 | 2.8674057 | up | noncoding | NONMMUT119835.1 |
| A_66_P123842 | 0.007524711 | 3.9630754 | up | noncoding | XR_888397 |
| CUST_OEV3_003350 | 0.035230603 | 3.0391667 | down | noncoding | NONMMUT136053.1 |
| CUST_OEV3_033722 | 0.012036414 | 2.4320304 | up | noncoding | NONMMUT095946.1 |
| CUST_OEV3_017083 | 0.019360898 | 2.0872047 | down | noncoding | NONMMUT118035.1 |
| CUST_OEV3_015472 | 0.017424941 | 2.3489575 | down | noncoding | NONMMUT120300.1 |
| CUST_OEV3_002095 | 0.04798926 | 2.778904 | down | noncoding | NovelTID_00000298 |
| CUST_OEV3_074138 | 0.007776456 | 4.245728 | up | noncoding | AK083759 |
| CUST_OEV3_012035 | 0.020629311 | 2.280925 | up | noncoding | NONMMUT124809.1 |
| A_55_P2181429 | 0.004331195 | 14.813858 | up | noncoding | XR_866213 |
| CUST_OEV3_056908 | 0.011582098 | 2.2523196 | down | noncoding | XR_389479 |
| CUST_OEV3_053545 | 0.010248173 | 2.795507 | up | noncoding | AK036557 |
| CUST_OEV3_051694 | 0.035599515 | 2.7410924 | up | noncoding | NONMMUT069297.2 |
| CUST_OEV3_063952 | 0.017043974 | 3.5669353 | up | noncoding | NONMMUT032602.2 |
| CUST_OEV3_024429 | 0.011409885 | 2.4175124 | up | noncoding | NONMMUT108037.1 |
| CUST_OEV3_037617 | 0.012466084 | 2.1705606 | up | noncoding | NONMMUT090952.1 |
| CUST_OEV3_092760 | 0.048622537 | 4.0589075 | up | noncoding | ENSMUST00000189218.1 |
| CUST_OEV3_000543 | 0.025478577 | 3.0020385 | down | noncoding | NovelTID_00005532 |
| CUST_OEV3_063520 | 0.046399485 | 2.8858368 | up | noncoding | AK014465 |
| CUST_OEV3_062197 | 0.003557276 | 2.0631278 | up | noncoding | AK157806 |
| CUST_OEV3_016058 | 0.031008953 | 3.0792832 | up | noncoding | XR_402971 |
| CUST_OEV3_005534 | 0.014814789 | 2.2808964 | up | noncoding | NONMMUT133215.1 |
| CUST_OEV3_091862 | 1.89E-05 | 8.022038 | down | noncoding | NR_015609 |
| CUST_OEV3_004348 | 5.76E-04 | 7.423259 | up | noncoding | NONMMUT134748.1 |
| CUST_OEV3_058332 | 0.030877974 | 3.300061 | up | noncoding | XR_376612 |
| CUST_OEV3_054977 | 0.038511947 | 2.8826482 | down | noncoding | NONMMUT059091.2 |
| CUST_OEV3_037770 | 0.02879885 | 2.104249 | down | noncoding | NONMMUT090755.1 |
| CUST_OEV3_045799 | 0.032949623 | 2.6990528 | up | noncoding | NONMMUT080170.1 |
| CUST_OEV3_013099 | 0.005155574 | 3.1186666 | up | noncoding | NONMMUT123402.1 |
| CUST_OEV3_041917 | 0.028784432 | 6.033546 | up | noncoding | NONMMUT085212.1 |
| CUST_OEV3_074301 | 0.011103051 | 2.1532366 | up | noncoding | XR_387272 |
| CUST_OEV3_059516 | 0.033653982 | 2.0117679 | up | noncoding | NONMMUT045831.2 |
| CUST_OEV3_003578 | 0.035912227 | 2.9554384 | down | noncoding | NONMMUT135763.1 |
| CUST_OEV3_025765 | 0.01200556 | 5.797477 | up | noncoding | NONMMUT106258.1 |
| CUST_OEV3_052794 | 0.040212657 | 2.028055 | down | noncoding | NONMMUT066261.2 |
| CUST_OEV3_065747 | 0.043789964 | 2.3712823 | up | noncoding | XR_384970 |
| CUST_OEV3_010520 | 0.03959517 | 2.0162718 | up | noncoding | NONMMUT126823.1 |
| CUST_OEV3_016075 | 0.003429249 | 2.5949543 | up | noncoding | NONMMUT119419.1 |
| CUST_OEV3_001623 | 0.011604879 | 2.007074 | down | noncoding | NovelTID_00001942 |
| CUST_OEV3_073110 | 0.017113172 | 3.9684207 | down | noncoding | AK045764 |
| CUST_OEV3_024351 | 0.049286358 | 2.6016252 | up | noncoding | NONMMUT108152.1 |
| CUST_OEV3_004598 | 0.020055633 | 2.7654486 | up | noncoding | NONMMUT134421.1 |
| CUST_OEV3_058774 | 0.008531631 | 2.5142925 | up | noncoding | XR_390680 |
| CUST_OEV3_067494 | 0.038797338 | 3.8255982 | down | noncoding | XR_383799 |
| CUST_OEV3_065184 | 0.003320108 | 5.0090327 | down | noncoding | XR_385525 |
| CUST_OEV3_068223 | 0.045334384 | 2.943726 | up | noncoding | NONMMUT020328.2 |
| CUST_OEV3_008366 | 0.003444748 | 14.941828 | up | noncoding | NONMMUT129646.1 |
| CUST_OEV3_026590 | 0.007850376 | 2.035192 | up | noncoding | NONMMUT105166.1 |

| CUST_OEV3_018191 | 0.044468805 | 2.4414861 | up | noncoding | NONMMUT116609.1 |
| --- | --- | --- | --- | --- | --- |
| CUST_OEV3_064249 | 0.033278536 | 2.3419337 | up | noncoding | AK048751 |
| CUST_OEV3_025122 | 0.046194397 | 3.2696302 | down | noncoding | NONMMUT107122.1 |
| CUST_OEV3_054303 | 0.010432457 | 2.9046597 | up | noncoding | NONMMUT061703.2 |
| A_55_P2540424 | 0.043203987 | 2.275672 | up | noncoding | XR_401442 |
| CUST_OEV3_034303 | 0.040754292 | 2.6462178 | up | noncoding | NONMMUT095231.1 |
| CUST_OEV3_023582 | 0.028761486 | 2.2439928 | up | noncoding | NONMMUT109238.1 |
| CUST_OEV3_069645 | 0.009949229 | 2.9123306 | up | noncoding | NONMMUT016038.2 |
| CUST_OEV3_027234 | 0.044986244 | 2.332648 | down | noncoding | NONMMUT104317.1 |
| CUST_OEV3_091845 | 0.037249032 | 2.2285671 | down | noncoding | ENSMUST00000209515.1 |
| CUST_OEV3_042393 | 0.023712238 | 2.600102 | up | noncoding | NONMMUT084605.1 |
| CUST_OEV3_034705 | 0.04582348 | 2.2521658 | down | noncoding | NONMMUT094730.1 |
| CUST_OEV3_070910 | 0.02229389 | 4.547442 | up | noncoding | AK161768 |
| CUST_OEV3_072753 | 0.040469307 | 8.284565 | up | noncoding | NONMMUT006709.2 |
| CUST_OEV3_020896 | 0.046240132 | 2.441551 | down | noncoding | NONMMUT112935.1 |
| A_55_P2282331 | 0.024106322 | 2.3098106 | up | noncoding | NR_131015 |
| CUST_OEV3_021800 | 0.040712353 | 2.1667573 | down | noncoding | NONMMUT111672.1 |
| A_55_P2073288 | 0.019468466 | 3.2338293 | up | noncoding | NR_033467 |
| CUST_OEV3_049647 | 0.030721582 | 2.3590074 | up | noncoding | NONMMUT075178.1 |
| CUST_OEV3_050954 | 0.004913535 | 6.2619195 | down | noncoding | AK046563 |
| CUST_OEV3_021956 | 0.009470063 | 2.48523 | up | noncoding | NONMMUT111436.1 |
| CUST_OEV3_052814 | 0.033084463 | 2.2228894 | up | noncoding | AK043926 |
| CUST_OEV3_061264 | 0.045332827 | 2.8231614 | up | noncoding | NONMMUT040196.2 |
| CUST_OEV3_008727 | 0.00415284 | 4.889291 | up | noncoding | NONMMUT129223.1 |
| CUST_OEV3_003150 | 0.010303211 | 2.5056217 | up | noncoding | NONMMUT136356.1 |
| CUST_OEV3_072649 | 0.021116579 | 2.5992014 | up | noncoding | NONMMUT007006.2 |
| CUST_OEV3_032031 | 0.046141844 | 2.3481436 | up | noncoding | NONMMUT098138.1 |
| CUST_OEV3_001815 | 0.014869043 | 5.0780096 | up | noncoding | NovelTID_00001333 |
| CUST_OEV3_052152 | 0.005087136 | 2.6286297 | up | noncoding | NONMMUT068122.2 |
| CUST_OEV3_090187 | 0.029503483 | 2.26292 | up | noncoding | AK157299 |
| CUST_OEV3_065160 | 0.025698394 | 2.5632255 | down | noncoding | XR_397413 |
| CUST_OEV3_005694 | 0.012969138 | 2.1403077 | down | noncoding | XR_379683 |
| CUST_OEV3_003403 | 0.006097118 | 2.0231009 | up | noncoding | NONMMUT135985.1 |
| CUST_OEV3_029380 | 0.04963226 | 2.2637284 | down | noncoding | XR_397702 |
| CUST_OEV3_029584 | 0.023780799 | 3.1149187 | up | noncoding | NONMMUT101260.1 |
| CUST_OEV3_069144 | 0.040068004 | 2.327235 | up | noncoding | XR_377601 |
| CUST_OEV3_069371 | 0.007284154 | 4.1473007 | up | noncoding | AK158512 |
| CUST_OEV3_072558 | 0.01047268 | 3.0285306 | up | noncoding | AK157498 |
| CUST_OEV3_013779 | 0.024715142 | 2.305999 | up | noncoding | NONMMUT122528.1 |
| CUST_OEV3_013071 | 0.049217142 | 2.0362103 | up | noncoding | NONMMUT123446.1 |
| CUST_OEV3_092355 | 0.03909682 | 2.056577 | down | noncoding | AK136680 |
| CUST_OEV3_032414 | 0.011780866 | 3.5128746 | up | noncoding | NONMMUT097628.1 |
| CUST_OEV3_015926 | 0.04898921 | 9.701025 | up | noncoding | AK043560 |
| CUST_OEV3_047549 | 0.045218915 | 2.9538174 | up | noncoding | NONMMUT077961.1 |
| CUST_OEV3_041145 | 0.012740235 | 2.9564927 | up | noncoding | XR_381259 |
| CUST_OEV3_066323 | 0.003446324 | 2.3387675 | up | noncoding | NONMMUT025786.2 |
| CUST_OEV3_059218 | 0.02321175 | 2.1281016 | up | noncoding | NONMMUT046624.2 |
| CUST_OEV3_023222 | 0.031867195 | 2.5239358 | down | noncoding | NONMMUT109710.1 |
| CUST_OEV3_057409 | 0.031411324 | 2.349117 | down | noncoding | NONMMUT052094.2 |
| CUST_OEV3_074870 | 0.007417715 | 2.277222 | up | noncoding | AK047560 |
| CUST_OEV3_025398 | 0.004210499 | 16.988857 | up | noncoding | NONMMUT106736.1 |
| A_66_P137451 | 7.61E-04 | 2.0915124 | down | noncoding | XR_376559 |
| CUST_OEV3_052172 | 0.032998454 | 4.5735536 | up | noncoding | NONMMUT068005.2 |
| CUST_OEV3_089848 | 0.03130326 | 3.169682 | down | noncoding | AK047110 |
| CUST_OEV3_008590 | 0.003948541 | 2.2687747 | up | noncoding | NONMMUT129397.1 |
| CUST_OEV3_025937 | 0.032788876 | 2.0329878 | down | noncoding | NONMMUT106042.1 |
| CUST_OEV3_053808 | 0.010815014 | 3.5815446 | up | noncoding | NONMMUT063235.2 |
| CUST_OEV3_029290 | 0.04076789 | 2.0345607 | down | noncoding | NONMMUT101634.1 |
| CUST_OEV3_027240 | 0.001174705 | 2.5053508 | up | noncoding | NONMMUT104309.1 |
| CUST_OEV3_015381 | 0.002380487 | 2.0131276 | up | noncoding | NONMMUT120415.1 |
| CUST_OEV3_035477 | 0.030876614 | 2.1716342 | down | noncoding | NONMMUT093747.1 |
| CUST_OEV3_031106 | 0.028733566 | 2.3658705 | down | noncoding | AV307790 |
| CUST_OEV3_009062 | 0.01544317 | 2.795361 | down | noncoding | NONMMUT128781.1 |

| CUST_OEV3_047921 | 0.027285086 | 2.0549436 | up | noncoding | NONMMUT077473.1 |
| --- | --- | --- | --- | --- | --- |
| CUST_OEV3_068269 | 0.03553484 | 5.275822 | up | noncoding | NONMMUT020180.2 |
| CUST_OEV3_058932 | 0.02760934 | 2.015387 | down | noncoding | NONMMUT047480.2 |
| CUST_OEV3_030868 | 0.008253799 | 2.5719652 | up | noncoding | NONMMUT099571.1 |
| CUST_OEV3_060099 | 0.03638682 | 2.167913 | up | noncoding | NONMMUT043959.2 |
| CUST_OEV3_000745 | 0.020479186 | 2.223724 | up | noncoding | NovelTID_00004941 |
| CUST_OEV3_054412 | 0.013419474 | 2.1268165 | up | noncoding | AK041357 |
| CUST_OEV3_037603 | 0.027714903 | 2.0339072 | down | noncoding | NONMMUT090967.1 |
| CUST_OEV3_046832 | 0.005984215 | 2.3891025 | up | noncoding | NONMMUT078808.1 |
| CUST_OEV3_024388 | 0.010903498 | 2.6426654 | up | noncoding | NONMMUT108095.1 |
| CUST_OEV3_048429 | 0.032774925 | 2.5506313 | down | noncoding | NONMMUT076803.1 |
| CUST_OEV3_091746 | 1.42E-04 | 5.9856825 | up | noncoding | ENSMUST00000210553.1 |
| CUST_OEV3_021535 | 0.048251487 | 3.4380362 | up | noncoding | NONMMUT112040.1 |
| CUST_OEV3_091146 | 0.001348831 | 13.683378 | up | noncoding | AK156997 |
| CUST_OEV3_015458 | 0.027096612 | 3.1883154 | up | noncoding | NONMMUT120316.1 |
| CUST_OEV3_002941 | 0.002889112 | 12.61382 | up | noncoding | NONMMUT136625.1 |
| CUST_OEV3_041496 | 0.040465865 | 4.1081104 | up | noncoding | NONMMUT085814.1 |
| A_66_P128000 | 0.018134238 | 2.1220305 | up | noncoding | NR_131758 |
| CUST_OEV3_024856 | 0.031469557 | 3.0710206 | down | noncoding | NONMMUT107464.1 |
| CUST_OEV3_015879 | 0.027692698 | 2.2238295 | down | noncoding | NONMMUT119710.1 |
| CUST_OEV3_023602 | 0.009270468 | 2.0982175 | up | noncoding | NONMMUT109211.1 |
| CUST_OEV3_009013 | 0.016001131 | 4.810177 | down | noncoding | NONMMUT128855.1 |
| CUST_OEV3_020453 | 0.003671369 | 2.5245683 | up | noncoding | NONMMUT113546.1 |
| CUST_OEV3_041105 | 0.019134142 | 2.004292 | down | noncoding | NONMMUT086322.1 |
| CUST_OEV3_065058 | 0.017840456 | 2.1938398 | up | noncoding | NONMMUT029556.2 |
| CUST_OEV3_038515 | 0.019382415 | 2.6955936 | up | noncoding | NONMMUT089736.1 |
| CUST_OEV3_044496 | 0.009057083 | 3.1493447 | down | noncoding | NONMMUT081882.1 |
| CUST_OEV3_070136 | 0.0275308 | 5.993762 | down | noncoding | AK051153 |
| A_55_P2555095 | 0.018119676 | 2.494742 | down | noncoding | XR_394588 |
| CUST_OEV3_055549 | 0.004490025 | 3.8978329 | up | noncoding | NONMMUT057439.2 |
| CUST_OEV3_069143 | 0.044255655 | 2.1066258 | down | noncoding | AK017047 |
| CUST_OEV3_003030 | 0.0025615 | 24.866907 | up | noncoding | NONMMUT136502.1 |
| CUST_OEV3_011802 | 0.010769198 | 2.1692357 | up | noncoding | NONMMUT125141.1 |
| CUST_OEV3_020869 | 0.016088622 | 2.2771986 | down | noncoding | NONMMUT112964.1 |
| CUST_OEV3_092576 | 0.007354906 | 2.0860047 | up | noncoding | BY742167 |
| CUST_OEV3_068803 | 0.005355455 | 3.3886232 | up | noncoding | AK031372 |
| A_55_P2018681 | 6.59E-04 | 2.3138735 | up | noncoding | XR_886092 |
| CUST_OEV3_072247 | 0.001366487 | 2.2458255 | down | noncoding | AK041473 |
| CUST_OEV3_010231 | 0.018981377 | 2.0892277 | down | noncoding | NONMMUT127233.1 |
| CUST_OEV3_090646 | 0.004830141 | 2.7096438 | up | noncoding | AK139775 |
| CUST_OEV3_014139 | 0.003174804 | 5.022693 | up | noncoding | NONMMUT122035.1 |
| CUST_OEV3_063552 | 0.013796886 | 5.097624 | up | noncoding | AK089816 |
| CUST_OEV3_043989 | 0.018128527 | 2.0359814 | down | noncoding | NONMMUT082549.1 |
| CUST_OEV3_050277 | 0.03068859 | 2.0721524 | down | noncoding | AK054371 |
| CUST_OEV3_070410 | 0.046151556 | 2.4697855 | down | noncoding | AK047781 |
| CUST_OEV3_030831 | 0.006594135 | 7.9001327 | up | noncoding | NONMMUT099623.1 |
| CUST_OEV3_065182 | 0.020396441 | 2.28424 | down | noncoding | AK037071 |
| CUST_OEV3_037486 | 0.03500459 | 2.1743832 | up | noncoding | NONMMUT091131.1 |
| CUST_OEV3_061104 | 0.036731526 | 2.2106392 | down | noncoding | NONMMUT040651.2 |
| CUST_OEV3_049406 | 0.003941003 | 3.5934825 | up | noncoding | NONMMUT075505.1 |
| CUST_OEV3_035088 | 0.013453655 | 3.8645072 | up | noncoding | NONMMUT094223.1 |
| CUST_OEV3_004579 | 0.046212405 | 2.6200426 | up | noncoding | NONMMUT134448.1 |
| CUST_OEV3_017532 | 0.041542906 | 2.6316829 | down | noncoding | NONMMUT117472.1 |
| CUST_OEV3_029077 | 0.024607444 | 7.8616624 | up | noncoding | NONMMUT101921.1 |
| CUST_OEV3_023388 | 0.013215005 | 2.0172718 | down | noncoding | NONMMUT109470.1 |
| CUST_OEV3_056955 | 0.020599958 | 2.8529963 | up | noncoding | NONMMUT053377.2 |
| A_52_P590995 | 0.04250986 | 2.5777214 | down | noncoding | NR_036452 |
| CUST_OEV3_026631 | 0.036506563 | 2.2775683 | up | noncoding | AK142309 |
| CUST_OEV3_091321 | 0.014442287 | 2.1732073 | up | noncoding | AK140080 |
| CUST_OEV3_012154 | 0.043581102 | 2.334533 | down | noncoding | NONMMUT124623.1 |
| CUST_OEV3_030879 | 0.022652451 | 2.5992968 | up | noncoding | AK077177 |
| CUST_OEV3_033761 | 0.03294504 | 2.2406406 | up | noncoding | NONMMUT095890.1 |
| CUST_OEV3_018910 | 4.60E-04 | 2.1342266 | up | noncoding | NONMMUT115626.1 |

| CUST_OEV3_022964 | 0.04995722 | 6.448987 | up | noncoding | NAP104620-1 |
| --- | --- | --- | --- | --- | --- |
| CUST_OEV3_035908 | 0.017695095 | 2.225027 | up | noncoding | NONMMUT093211.1 |
| CUST_OEV3_030948 | 0.047327004 | 2.0232575 | up | noncoding | NONMMUT099463.1 |
| CUST_OEV3_040361 | 0.005260977 | 2.388026 | up | noncoding | NONMMUT087303.1 |
| CUST_OEV3_008804 | 0.006744944 | 2.034496 | down | noncoding | NONMMUT129140.1 |
| CUST_OEV3_046836 | 0.001042242 | 2.1906126 | up | noncoding | NONMMUT078802.1 |
| CUST_OEV3_091420 | 0.035816636 | 2.9274275 | up | noncoding | KnowTID_00000902 |
| CUST_OEV3_035867 | 0.038851142 | 2.477457 | up | noncoding | NONMMUT093258.1 |
| CUST_OEV3_074298 | 0.006807564 | 3.0715768 | up | noncoding | NONMMUT002042.2 |
| CUST_OEV3_002453 | 0.005430057 | 2.2968862 | up | noncoding | NONMMUT137307.1 |
| CUST_OEV3_011582 | 0.034647603 | 2.6687117 | down | noncoding | NONMMUT125430.1 |
| CUST_OEV3_017537 | 0.006087105 | 2.95445 | up | noncoding | NONMMUT117465.1 |
| CUST_OEV3_074801 | 0.049721945 | 2.2983344 | up | noncoding | NONMMUT000515.2 |
| CUST_OEV3_032902 | 0.02060132 | 2.4042647 | up | noncoding | NONMMUT097003.1 |
| CUST_OEV3_066544 | 0.033007767 | 2.0136225 | down | noncoding | NR_047528 |
| CUST_OEV3_019186 | 0.041010078 | 2.1541336 | down | noncoding | NONMMUT115265.1 |
| CUST_OEV3_052535 | 0.002700106 | 3.2482598 | up | noncoding | AK148814 |
| CUST_OEV3_045392 | 0.003835487 | 2.1508934 | up | noncoding | NONMMUT080715.1 |
| CUST_OEV3_042623 | 2.10E-04 | 4.0809307 | up | noncoding | NONMMUT084310.1 |
| CUST_OEV3_035065 | 0.047212176 | 2.9807866 | down | noncoding | NONMMUT094253.1 |
| CUST_OEV3_054883 | 0.046070706 | 3.174595 | up | noncoding | NONMMUT059391.2 |
| CUST_OEV3_061733 | 0.018416697 | 2.0416162 | down | noncoding | AK150031 |
| CUST_OEV3_014245 | 0.006873109 | 2.5264857 | up | noncoding | NONMMUT121883.1 |
| CUST_OEV3_052919 | 0.037425566 | 4.520188 | up | noncoding | NONMMUT065895.2 |
| CUST_OEV3_060971 | 0.023923598 | 2.1953514 | up | noncoding | NONMMUT041088.2 |
| CUST_OEV3_070291 | 0.02569686 | 2.0554197 | down | noncoding | AK087341 |
| CUST_OEV3_071091 | 0.03514499 | 4.8165135 | up | noncoding | NONMMUT011688.2 |
| CUST_OEV3_040218 | 0.014746126 | 2.4175873 | up | noncoding | NONMMUT087482.1 |
| CUST_OEV3_034553 | 0.02085828 | 2.173007 | up | noncoding | NONMMUT094901.1 |
| A_66_P134336 | 0.049813867 | 3.0492485 | down | noncoding | NR_045049 |
| CUST_OEV3_034477 | 3.23E-04 | 7.623864 | up | noncoding | NONMMUT094997.1 |
| CUST_OEV3_000283 | 0.008738821 | 5.0415397 | up | noncoding | NovelTID_00006330 |
| CUST_OEV3_057534 | 0.049633022 | 2.380466 | up | noncoding | ENSMUST00000131628 |
| CUST_OEV3_016725 | 0.013554156 | 2.584983 | down | noncoding | NONMMUT118508.1 |
| CUST_OEV3_025830 | 0.026392845 | 2.2649045 | up | noncoding | NONMMUT106181.1 |
| CUST_OEV3_031212 | 0.006607301 | 2.40511 | up | noncoding | NONMMUT099125.1 |
| CUST_OEV3_069467 | 0.04939498 | 2.4571576 | down | noncoding | AK047083 |
| CUST_OEV3_074370 | 0.036962077 | 2.2171817 | down | noncoding | AK156286 |
| CUST_OEV3_011969 | 0.005430432 | 2.633073 | up | noncoding | NONMMUT124907.1 |
| CUST_OEV3_067293 | 0.02100659 | 5.1259236 | up | noncoding | AK140285 |
| CUST_OEV3_048254 | 0.03872077 | 2.0222719 | down | noncoding | NONMMUT077022.1 |
| A_55_P2080446 | 0.012702849 | 3.5034513 | up | noncoding | NR_102349 |
| CUST_OEV3_057289 | 0.011777165 | 2.114359 | up | noncoding | BG792746 |
| CUST_OEV3_020579 | 0.001335106 | 25.312557 | up | noncoding | NONMMUT113376.1 |
| CUST_OEV3_031776 | 0.01349777 | 2.3145359 | up | noncoding | NONMMUT098448.1 |
| CUST_OEV3_090267 | 0.017810201 | 5.7128386 | down | noncoding | AK075639 |
| CUST_OEV3_021668 | 0.024139138 | 2.3452148 | up | noncoding | NONMMUT111855.1 |
| CUST_OEV3_036292 | 2.85E-05 | 5.0180984 | up | noncoding | NONMMUT092717.1 |
| CUST_OEV3_021950 | 0.015466288 | 2.22355 | up | noncoding | NONMMUT111444.1 |
| CUST_OEV3_061517 | 0.01536707 | 3.8091526 | up | noncoding | AK046956 |
| CUST_OEV3_042654 | 0.041969325 | 4.5469866 | up | noncoding | NONMMUT084260.1 |
| CUST_OEV3_020548 | 0.024971334 | 2.1812866 | up | noncoding | NONMMUT113410.1 |
| CUST_OEV3_003063 | 0.02548234 | 3.1308455 | up | noncoding | NONMMUT136465.1 |
| CUST_OEV3_036676 | 0.017706988 | 2.487757 | down | noncoding | NONMMUT092214.1 |
| CUST_OEV3_031133 | 0.032410525 | 3.3435016 | up | noncoding | NONMMUT099223.1 |
| CUST_OEV3_006148 | 0.014631472 | 8.4763975 | up | noncoding | AK156661 |
| CUST_OEV3_042138 | 0.017397009 | 2.2025292 | up | noncoding | NONMMUT084942.1 |
| CUST_OEV3_042876 | 0.018503798 | 2.4103343 | up | noncoding | NONMMUT083970.1 |
| CUST_OEV3_067106 | 0.028813627 | 2.1837595 | down | noncoding | NONMMUT023486.2 |
| CUST_OEV3_022374 | 0.001067557 | 2.4996455 | up | noncoding | NONMMUT110864.1 |
| CUST_OEV3_074704 | 0.037291255 | 2.1780925 | up | noncoding | AK141444 |
| CUST_OEV3_013722 | 0.032549985 | 3.6713886 | up | noncoding | NONMMUT122594.1 |
| CUST_OEV3_028290 | 0.04812655 | 2.8195004 | up | noncoding | NONMMUT103008.1 |

| CUST_OEV3_032385 | 0.005553716 | 2.163068 | up | noncoding | AK037373 |
| --- | --- | --- | --- | --- | --- |
| CUST_OEV3_067515 | 0.03597442 | 11.96425 | up | noncoding | AK049069 |
| CUST_OEV3_063558 | 0.011624082 | 3.2970824 | up | noncoding | AK145157 |
| CUST_OEV3_057561 | 0.012514029 | 2.1887012 | up | noncoding | AK033398 |
| CUST_OEV3_091941 | 0.021811226 | 2.0017579 | down | noncoding | BY716422 |
| CUST_OEV3_047093 | 0.03818124 | 3.2836542 | up | noncoding | NONMMUT078524.1 |
| CUST_OEV3_010224 | 0.002768559 | 3.3354373 | down | noncoding | NONMMUT127240.1 |
| CUST_OEV3_047705 | 0.001264175 | 5.9899387 | up | noncoding | NONMMUT077748.1 |
| CUST_OEV3_037401 | 0.037343565 | 2.0269506 | down | noncoding | NONMMUT091236.1 |
| CUST_OEV3_067271 | 0.012833514 | 5.146274 | up | noncoding | AK016812 |
| CUST_OEV3_037456 | 0.024474243 | 3.3549752 | up | noncoding | NONMMUT091168.1 |
| CUST_OEV3_092622 | 0.022988502 | 2.314461 | up | noncoding | AK142999 |
| CUST_OEV3_055658 | 0.020877203 | 7.0119348 | up | noncoding | AK142237 |
| CUST_OEV3_045926 | 0.010536709 | 3.1678367 | up | noncoding | NONMMUT080006.1 |
| CUST_OEV3_040096 | 0.02257634 | 2.0224898 | down | noncoding | NONMMUT087648.1 |
| CUST_OEV3_026502 | 1.92E-04 | 2.766776 | up | noncoding | NONMMUT105283.1 |
| CUST_OEV3_059052 | 0.013732092 | 5.3360686 | up | noncoding | TC1680360 |
| A_52_P113830 | 0.014627642 | 3.305055 | up | noncoding | NR_038168 |
| CUST_OEV3_021432 | 0.009726103 | 2.7606094 | up | noncoding | NONMMUT112179.1 |
| CUST_OEV3_062639 | 0.024705956 | 2.0098922 | up | noncoding | BC023886 |
| CUST_OEV3_060589 | 0.024575127 | 2.1492484 | down | noncoding | AK044139 |
| CUST_OEV3_008792 | 0.037732482 | 2.293533 | down | noncoding | NONMMUT129155.1 |
| CUST_OEV3_072045 | 0.044331614 | 6.829676 | up | noncoding | NONMMUT008897.2 |
| CUST_OEV3_020514 | 3.37E-04 | 4.5945954 | up | noncoding | NONMMUT113459.1 |
| CUST_OEV3_054493 | 0.02198395 | 2.5865617 | up | noncoding | NONMMUT060812.2 |
| CUST_OEV3_047364 | 0.011782911 | 2.1218202 | down | noncoding | NONMMUT078187.1 |
| CUST_OEV3_052035 | 0.004366971 | 2.4272957 | up | noncoding | NONMMUT068428.2 |
| CUST_OEV3_012716 | 0.003413487 | 2.5262268 | down | noncoding | NONMMUT123901.1 |
| CUST_OEV3_057243 | 0.011559931 | 3.088882 | up | noncoding | AK017927 |
| CUST_OEV3_054576 | 0.003707021 | 6.613187 | up | noncoding | NONMMUT060442.2 |
| CUST_OEV3_068521 | 0.034233775 | 2.4871576 | up | noncoding | AK157155 |
| CUST_OEV3_061490 | 0.044802293 | 2.3731422 | down | noncoding | AK006813 |
| CUST_OEV3_057027 | 0.037415653 | 3.0428636 | down | noncoding | NONMMUT053156.2 |
| CUST_OEV3_030036 | 0.005242735 | 2.0929217 | up | noncoding | XR_385230 |
| CUST_OEV3_059132 | 0.0425296 | 2.1699445 | up | noncoding | AK044733 |
| CUST_OEV3_025722 | 0.005528973 | 3.355634 | up | noncoding | NONMMUT106316.1 |
| A_66_P129387 | 0.012861565 | 6.1349955 | up | noncoding | NR_110509 |
| CUST_OEV3_064788 | 0.012510297 | 2.0839224 | up | noncoding | NONMMUT030312.2 |
| CUST_OEV3_012336 | 0.025103658 | 2.3663685 | up | noncoding | NONMMUT124386.1 |
| CUST_OEV3_061608 | 0.012618801 | 2.3474157 | up | noncoding | NONMMUT039224.2 |
| CUST_OEV3_001023 | 0.03836967 | 2.7762747 | down | noncoding | NovelTID_00004027 |
| CUST_OEV3_050594 | 0.036465365 | 4.69082 | up | noncoding | AK089595 |
| CUST_OEV3_061501 | 0.020406544 | 2.180576 | up | noncoding | AK080236 |
| CUST_OEV3_068155 | 0.011659593 | 2.7078488 | up | noncoding | NAP061352-1 |
| CUST_OEV3_034246 | 0.03639746 | 2.1237762 | down | noncoding | NONMMUT095300.1 |
| CUST_OEV3_010464 | 0.008933266 | 6.3932815 | up | noncoding | NONMMUT126890.1 |
| CUST_OEV3_090370 | 0.011641478 | 2.994282 | up | noncoding | AK035544 |
| CUST_OEV3_066274 | 0.0482962 | 2.2523122 | up | noncoding | AK014756 |
| CUST_OEV3_071110 | 0.045872346 | 3.6950214 | up | noncoding | NONMMUT011635.2 |
| CUST_OEV3_046311 | 0.045244 | 2.274457 | down | noncoding | NONMMUT079504.1 |
| CUST_OEV3_056953 | 0.015540342 | 2.121555 | down | noncoding | AK013703 |
| A_55_P2572547 | 0.002909394 | 2.7420213 | up | noncoding | XR_377301 |
| CUST_OEV3_005447 | 0.04822296 | 2.0709934 | up | noncoding | NONMMUT133331.1 |
| CUST_OEV3_034722 | 0.01300357 | 2.3398974 | up | noncoding | NONMMUT094712.1 |
| A_51_P422223 | 0.04506549 | 2.1284957 | up | noncoding | XR_888044 |
| CUST_OEV3_064727 | 0.006409845 | 2.3387482 | up | noncoding | AK033207 |
| CUST_OEV3_021580 | 0.014444583 | 2.778252 | up | noncoding | AK019678 |
| CUST_OEV3_020135 | 0.046072762 | 2.1752684 | up | noncoding | NONMMUT113988.1 |
| CUST_OEV3_066414 | 0.04245047 | 2.8600476 | up | noncoding | NONMMUT025485.2 |
| CUST_OEV3_027920 | 0.048342343 | 2.8445208 | up | noncoding | NONMMUT103457.1 |
| CUST_OEV3_036287 | 0.03742103 | 2.0308542 | down | noncoding | NONMMUT092725.1 |
| CUST_OEV3_021689 | 0.031936325 | 3.1606083 | up | noncoding | NONMMUT111826.1 |
| CUST_OEV3_006306 | 0.015714778 | 2.3160725 | up | noncoding | NONMMUT132227.1 |

| CUST_OEV3_027506 | 0.01346927 | 2.0887947 | down | noncoding | NONMMUT103979.1 |
| --- | --- | --- | --- | --- | --- |
| CUST_OEV3_064399 | 0.010595403 | 2.4123898 | up | noncoding | AK037547 |
| CUST_OEV3_054902 | 8.75E-04 | 3.3104508 | down | noncoding | NONMMUT059337.2 |
| CUST_OEV3_074274 | 0.01908938 | 2.334831 | up | noncoding | NAP068607-1 |
| CUST_OEV3_032472 | 0.028667541 | 3.1825795 | up | noncoding | NONMMUT097550.1 |
| CUST_OEV3_034895 | 0.012158982 | 4.936956 | up | noncoding | NONMMUT094485.1 |
| CUST_OEV3_041360 | 0.01392756 | 3.4631438 | down | noncoding | NONMMUT086004.1 |
| CUST_OEV3_055603 | 0.034086786 | 5.1539865 | up | noncoding | NONMMUT057296.2 |
| CUST_OEV3_052722 | 0.006949421 | 2.3300698 | down | noncoding | NONMMUT066456.2 |
| CUST_OEV3_089932 | 0.039383687 | 3.104314 | down | noncoding | AK138612 |
| CUST_OEV3_033209 | 0.0341395 | 2.8054304 | down | noncoding | NONMMUT096600.1 |
| CUST_OEV3_015968 | 0.006842911 | 2.4313493 | down | noncoding | BE851482 |
| A_51_P488960 | 8.01E-04 | 29.444168 | up | noncoding | XR_870399 |
| CUST_OEV3_026135 | 0.004984288 | 2.635377 | down | noncoding | NONMMUT105761.1 |
| CUST_OEV3_042536 | 0.040068146 | 3.9882514 | up | noncoding | NONMMUT084428.1 |
| CUST_OEV3_090343 | 0.020512938 | 3.849835 | up | noncoding | AK138758 |
| CUST_OEV3_017483 | 0.024307104 | 2.0992057 | up | noncoding | NONMMUT117529.1 |
| CUST_OEV3_050153 | 4.80E-04 | 2.7570086 | up | noncoding | NONMMUT073817.2 |
| CUST_OEV3_062709 | 0.013850227 | 2.4906204 | down | noncoding | NONMMUT036049.2 |
| CUST_OEV3_062845 | 4.46E-04 | 2.0232532 | up | noncoding | AK041898 |
| CUST_OEV3_025472 | 0.010979874 | 3.1388474 | up | noncoding | NONMMUT106639.1 |
| CUST_OEV3_036689 | 0.04065362 | 2.1840682 | up | noncoding | NONMMUT092200.1 |
| CUST_OEV3_068340 | 0.009296821 | 3.542545 | up | noncoding | TC1687655 |
| CUST_OEV3_057347 | 0.015779004 | 2.075759 | down | noncoding | NONMMUT052239.2 |
| CUST_OEV3_004460 | 0.020929076 | 2.0253313 | up | noncoding | NONMMUT134596.1 |
| CUST_OEV3_039798 | 0.028851733 | 2.7005384 | up | noncoding | NONMMUT088056.1 |
| CUST_OEV3_053098 | 0.007023137 | 5.8292747 | down | noncoding | NONMMUT065435.2 |
| CUST_OEV3_003547 | 0.028855959 | 2.103123 | down | noncoding | NONMMUT135799.1 |
| CUST_OEV3_005706 | 0.041096356 | 2.9379668 | down | noncoding | NONMMUT132999.1 |
| CUST_OEV3_026417 | 0.014327382 | 2.2789862 | down | noncoding | NONMMUT105395.1 |
| CUST_OEV3_012331 | 0.022324381 | 9.91537 | down | noncoding | NONMMUT124392.1 |
| CUST_OEV3_022005 | 0.009326951 | 5.2260823 | up | noncoding | NONMMUT111371.1 |
| CUST_OEV3_020819 | 0.023433125 | 2.2056923 | down | noncoding | NONMMUT113030.1 |
| CUST_OEV3_023013 | 0.03380462 | 2.0540552 | down | noncoding | NONMMUT109969.1 |
| CUST_OEV3_043974 | 0.035507537 | 2.6085422 | down | noncoding | NONMMUT082566.1 |
| CUST_OEV3_030511 | 0.01768943 | 2.233852 | down | noncoding | NONMMUT100020.1 |
| CUST_OEV3_090485 | 0.040350236 | 4.8894815 | up | noncoding | AK041984 |
| CUST_OEV3_013225 | 0.023903612 | 3.197866 | down | noncoding | NONMMUT123237.1 |
| CUST_OEV3_090523 | 9.17E-04 | 2.0072043 | down | noncoding | AK045052 |
| CUST_OEV3_057045 | 0.020814568 | 3.5283246 | down | noncoding | BU961416 |
| CUST_OEV3_008079 | 0.029799696 | 3.21232 | down | noncoding | XR_378748 |
| A_51_P368450 | 0.011306704 | 4.3848686 | down | noncoding | XR_403772 |
| CUST_OEV3_016745 | 0.003668997 | 4.0795565 | up | noncoding | NONMMUT118476.1 |
| CUST_OEV3_042726 | 0.024461769 | 4.772337 | up | noncoding | NONMMUT084171.1 |
| CUST_OEV3_035932 | 0.016825477 | 3.8414412 | down | noncoding | NONMMUT093181.1 |
| CUST_OEV3_016366 | 0.02962253 | 2.45067 | down | noncoding | NONMMUT119003.1 |
| CUST_OEV3_004030 | 0.00582344 | 3.9191506 | down | noncoding | NONMMUT135177.1 |
| CUST_OEV3_066045 | 0.0394531 | 2.5346842 | up | noncoding | AK048314 |
| CUST_OEV3_091825 | 0.04546995 | 5.005261 | up | noncoding | ENSMUST00000209674.1 |
| CUST_OEV3_048003 | 0.047471385 | 2.1869106 | down | noncoding | NONMMUT077361.1 |
| CUST_OEV3_019868 | 0.012814758 | 2.723633 | up | noncoding | NONMMUT114340.1 |
| CUST_OEV3_056180 | 0.021334872 | 3.1549842 | up | noncoding | AK035449 |
| CUST_OEV3_030248 | 0.013971437 | 2.1794698 | up | noncoding | NONMMUT100339.1 |
| CUST_OEV3_005896 | 0.013429915 | 2.2905772 | up | noncoding | XR_379571 |
| CUST_OEV3_040764 | 0.025135899 | 2.1595693 | up | noncoding | NONMMUT086775.1 |
| CUST_OEV3_024378 | 0.005037888 | 2.5648558 | up | noncoding | NONMMUT108109.1 |
| CUST_OEV3_039564 | 0.028790357 | 2.4260323 | down | noncoding | NONMMUT088337.1 |
| CUST_OEV3_004434 | 0.022145776 | 5.711236 | up | noncoding | NONMMUT134631.1 |
| CUST_OEV3_037221 | 0.01566239 | 3.197799 | up | noncoding | NONMMUT091456.1 |
| CUST_OEV3_090848 | 1.12E-04 | 2.3634295 | up | noncoding | AK132624 |
| CUST_OEV3_006874 | 0.002862069 | 3.5186105 | up | noncoding | NONMMUT131466.1 |
| CUST_OEV3_019549 | 0.03794275 | 2.0645015 | up | noncoding | NONMMUT114758.1 |
| CUST_OEV3_032416 | 0.027394826 | 2.2946143 | up | noncoding | XR_384384 |

| CUST_OEV3_051023 | 0.047210958 | 4.9417768 | up | noncoding | AK087903 |
| --- | --- | --- | --- | --- | --- |
| CUST_OEV3_067998 | 0.035120666 | 3.7970452 | up | noncoding | XR_383458 |
| CUST_OEV3_035835 | 0.028628714 | 2.2599802 | down | noncoding | NONMMUT093293.1 |
| CUST_OEV3_023011 | 0.007855076 | 2.5100613 | down | noncoding | NONMMUT109971.1 |
| CUST_OEV3_003652 | 0.037341624 | 2.3632689 | up | noncoding | NONMMUT135651.1 |
| CUST_OEV3_033185 | 0.022813143 | 6.4767437 | up | noncoding | NONMMUT096637.1 |
| CUST_OEV3_089962 | 4.73E-05 | 2.0511136 | down | noncoding | KnowTID_00007325 |
| CUST_OEV3_019786 | 0.049924295 | 2.4449313 | up | noncoding | NONMMUT114431.1 |
| CUST_OEV3_041942 | 0.007569074 | 11.535747 | up | noncoding | NONMMUT085180.1 |
| CUST_OEV3_050578 | 0.027761934 | 2.3291905 | down | noncoding | NONMMUT072717.2 |
| CUST_OEV3_017401 | 0.00356164 | 3.5938008 | up | noncoding | NONMMUT117630.1 |
| CUST_OEV3_037030 | 0.046174463 | 2.0510163 | down | noncoding | NONMMUT091730.1 |
| CUST_OEV3_023452 | 0.022497222 | 3.3413405 | up | noncoding | NONMMUT109391.1 |
| CUST_OEV3_040122 | 0.00350357 | 14.583159 | up | noncoding | NONMMUT087615.1 |
| CUST_OEV3_067518 | 0.047214177 | 2.4275868 | down | noncoding | XR_383788 |
| CUST_OEV3_027329 | 0.016241966 | 2.8320277 | up | noncoding | NONMMUT104199.1 |
| CUST_OEV3_044596 | 0.019177616 | 2.016633 | up | noncoding | NONMMUT081742.1 |
| CUST_OEV3_012556 | 0.029886384 | 2.4667432 | down | noncoding | NONMMUT124109.1 |
| CUST_OEV3_047605 | 0.03390328 | 3.3531828 | up | noncoding | NONMMUT077886.1 |
| CUST_OEV3_007035 | 0.036450557 | 3.166799 | up | noncoding | NONMMUT131263.1 |
| CUST_OEV3_051257 | 0.016706228 | 3.342328 | down | noncoding | AK051854 |
| CUST_OEV3_009719 | 0.003397195 | 2.2574728 | up | noncoding | NONMMUT127927.1 |
| CUST_OEV3_060091 | 0.047285732 | 3.4933176 | down | noncoding | AK038277 |
| CUST_OEV3_042565 | 0.02220021 | 2.0655172 | up | noncoding | NONMMUT084385.1 |
| CUST_OEV3_026914 | 0.03137575 | 3.7717214 | down | noncoding | NONMMUT104707.1 |
| CUST_OEV3_092053 | 0.048813418 | 2.067209 | up | noncoding | ENSMUST00000206886.1 |
| CUST_OEV3_091788 | 0.004589055 | 2.1073263 | up | noncoding | XR_378938 |
| CUST_OEV3_011796 | 0.018287646 | 2.1903439 | up | noncoding | NONMMUT125149.1 |
| A_66_P106320 | 0.005425262 | 2.117794 | down | noncoding | XR_870359 |
| CUST_OEV3_043389 | 0.018472483 | 5.3701043 | up | noncoding | NONMMUT083287.1 |
| A_66_P126104 | 0.002975473 | 2.406778 | down | noncoding | NR_045819 |
| CUST_OEV3_042626 | 0.03968899 | 3.2071939 | up | noncoding | NONMMUT084307.1 |
| CUST_OEV3_051188 | 0.007277549 | 2.8428624 | up | noncoding | AK015353 |
| CUST_OEV3_014261 | 0.001887346 | 5.5053763 | up | noncoding | NONMMUT121863.1 |
| CUST_OEV3_015071 | 0.001975855 | 2.5242553 | down | noncoding | NONMMUT120808.1 |
| CUST_OEV3_068966 | 9.94E-04 | 23.868917 | up | noncoding | NONMMUT017997.2 |
| CUST_OEV3_046752 | 0.007639435 | 3.8810773 | up | noncoding | NONMMUT078929.1 |
| CUST_OEV3_007753 | 0.016285418 | 3.4406133 | up | noncoding | NONMMUT130395.1 |
| CUST_OEV3_017730 | 0.031301852 | 2.2884629 | down | noncoding | NONMMUT117224.1 |
| CUST_OEV3_002691 | 0.01477828 | 2.3584402 | up | noncoding | TC1645746 |
| A_55_P2135203 | 0.004626252 | 3.2826076 | up | noncoding | XR_381388 |
| CUST_OEV3_068213 | 0.027811922 | 4.4456334 | up | noncoding | NONMMUT020353.2 |
| CUST_OEV3_062506 | 0.03963591 | 2.2216012 | up | noncoding | NONMMUT036921.2 |
| CUST_OEV3_001662 | 0.044958223 | 3.9661312 | up | noncoding | NovelTID_00001826 |
| CUST_OEV3_037460 | 0.031552225 | 3.3542612 | up | noncoding | NONMMUT091164.1 |
| CUST_OEV3_015866 | 0.010794898 | 6.2546873 | down | noncoding | NONMMUT119727.1 |
| CUST_OEV3_002723 | 0.011957286 | 2.0411372 | down | noncoding | NONMMUT136920.1 |
| CUST_OEV3_055415 | 0.014560262 | 3.867877 | up | noncoding | NONMMUT057855.2 |
| A_66_P124878 | 0.008835057 | 2.723471 | up | noncoding | XR_373735 |
| CUST_OEV3_002257 | 0.00159623 | 3.840103 | up | noncoding | NONMMUT137579.1 |
| CUST_OEV3_062605 | 0.023211142 | 4.003607 | up | noncoding | NONMMUT036490.2 |
| CUST_OEV3_009054 | 0.02484511 | 4.8306513 | up | noncoding | NONMMUT128790.1 |
| CUST_OEV3_035026 | 0.013139339 | 4.3483834 | up | noncoding | NONMMUT094306.1 |
| CUST_OEV3_025167 | 0.045143317 | 3.4432333 | down | noncoding | NONMMUT107052.1 |
| CUST_OEV3_000003 | 0.024284568 | 2.707727 | down | noncoding | AK137587 |
| CUST_OEV3_059881 | 0.024834465 | 2.2143686 | down | noncoding | AK083142 |
| CUST_OEV3_005362 | 0.015393883 | 2.622829 | up | noncoding | TC1687982 |
| CUST_OEV3_058485 | 0.03419903 | 2.4111693 | up | noncoding | NONMMUT048587.2 |
| A_55_P2580694 | 0.047258146 | 4.2758646 | up | noncoding | NR_131156 |
| CUST_OEV3_011580 | 0.012725162 | 2.8870542 | up | noncoding | NONMMUT125433.1 |
| CUST_OEV3_073353 | 0.030908823 | 2.1650393 | down | noncoding | XR_380218 |
| CUST_OEV3_062816 | 0.02026753 | 2.0259137 | down | noncoding | AK053057 |
| CUST_OEV3_090713 | 0.015770182 | 2.6582198 | up | noncoding | AK086035 |

| CUST_OEV3_036450 | 0.035658043 | 2.014974 | down | noncoding | NONMMUT092519.1 |
| --- | --- | --- | --- | --- | --- |
| CUST_OEV3_054731 | 0.007828755 | 2.2350993 | down | noncoding | AK040075 |
| CUST_OEV3_025397 | 0.006119763 | 3.2920625 | up | noncoding | NONMMUT106737.1 |
| CUST_OEV3_033764 | 0.021541236 | 2.0799353 | down | noncoding | NONMMUT095887.1 |
| CUST_OEV3_073831 | 0.029665768 | 2.9377904 | down | noncoding | NONMMUT003589.2 |
| CUST_OEV3_092587 | 0.01548144 | 3.517115 | up | noncoding | ENSMUST00000194390.1 |
| A_66_P114115 | 0.02449814 | 3.4545047 | up | noncoding | XR_861541 |
| CUST_OEV3_040249 | 0.005478075 | 2.1054883 | up | noncoding | XR_381793 |
| CUST_OEV3_038596 | 0.025828509 | 2.6411884 | down | noncoding | NONMMUT089614.1 |
| CUST_OEV3_024772 | 0.03706934 | 2.5980694 | down | noncoding | NONMMUT107577.1 |
| CUST_OEV3_072638 | 0.02848852 | 2.4111557 | up | noncoding | NONMMUT007052.2 |
| CUST_OEV3_064734 | 0.011550233 | 2.5206544 | up | noncoding | NONMMUT030436.2 |
| CUST_OEV3_066153 | 0.027688898 | 3.7711105 | up | noncoding | AK046362 |
| CUST_OEV3_006975 | 0.049656384 | 2.566219 | up | noncoding | XR_387878 |
| CUST_OEV3_035178 | 0.030317621 | 2.5359502 | up | noncoding | NONMMUT094116.1 |
| CUST_OEV3_038154 | 0.008029952 | 2.6468813 | up | noncoding | NONMMUT090226.1 |
| CUST_OEV3_058723 | 0.049153715 | 3.3920321 | down | noncoding | AK034073 |
| CUST_OEV3_027015 | 0.023983786 | 2.4116223 | down | noncoding | NONMMUT104586.1 |
| A_66_P121259 | 0.030546745 | 9.102482 | up | noncoding | XR_884721 |
| CUST_OEV3_038467 | 0.041666005 | 2.8073978 | up | noncoding | NONMMUT089807.1 |
| CUST_OEV3_045223 | 0.041563053 | 2.077683 | up | noncoding | NONMMUT080931.1 |
| A_52_P84347 | 0.019230524 | 2.2683604 | up | noncoding | NR_040593 |
| CUST_OEV3_009714 | 0.005976027 | 2.0655103 | down | noncoding | NONMMUT127935.1 |
| A_52_P624599 | 0.034211993 | 2.711491 | down | noncoding | XR_382470 |
| CUST_OEV3_037354 | 0.035535656 | 2.2682436 | up | noncoding | NONMMUT091286.1 |
| CUST_OEV3_069588 | 0.044309165 | 2.404296 | down | noncoding | NONMMUT016226.2 |
| CUST_OEV3_041302 | 0.008429552 | 2.2584388 | up | noncoding | NONMMUT086085.1 |
| CUST_OEV3_056597 | 0.03886941 | 2.5369344 | up | noncoding | NR_015517 |
| CUST_OEV3_068501 | 0.018067367 | 2.571428 | up | noncoding | AK047920 |
| CUST_OEV3_027569 | 0.012297434 | 2.670223 | down | noncoding | NONMMUT103894.1 |
| CUST_OEV3_040098 | 0.003495239 | 5.5986075 | up | noncoding | NONMMUT087645.1 |
| CUST_OEV3_030703 | 0.049091358 | 2.53533 | down | noncoding | NONMMUT099801.1 |
| CUST_OEV3_008554 | 0.008967279 | 3.999961 | down | noncoding | NONMMUT129434.1 |
| CUST_OEV3_026721 | 0.039824758 | 2.0954 | down | noncoding | NAP065879-1 |
| CUST_OEV3_015416 | 0.034429137 | 2.015335 | up | noncoding | NONMMUT120374.1 |
| CUST_OEV3_053731 | 0.004754087 | 4.294292 | up | noncoding | TC1718833 |
| CUST_OEV3_018890 | 0.045908563 | 2.5393748 | down | noncoding | NONMMUT115658.1 |
| CUST_OEV3_055276 | 0.030406052 | 2.107334 | down | noncoding | NONMMUT058222.2 |
| A_55_P2337706 | 0.004458932 | 11.354245 | up | noncoding | XR_387068 |
| A_55_P2543413 | 0.036066968 | 3.2312064 | up | noncoding | XR_880568 |
| CUST_OEV3_038782 | 0.03156938 | 3.3224058 | down | noncoding | NONMMUT089367.1 |
| CUST_OEV3_073489 | 0.006791299 | 2.2016332 | down | noncoding | AK140985 |
| CUST_OEV3_053628 | 0.006661269 | 2.2799513 | down | noncoding | AK087072 |
| CUST_OEV3_064020 | 0.027792834 | 2.2277484 | down | noncoding | AK079263 |
| CUST_OEV3_000232 | 0.007500887 | 6.3059487 | up | noncoding | NovelTID_00006474 |
| CUST_OEV3_062443 | 0.03708119 | 2.7308934 | up | noncoding | NONMMUT037050.2 |
| CUST_OEV3_022472 | 0.009778253 | 2.6568787 | up | noncoding | NONMMUT110746.1 |
| CUST_OEV3_016209 | 0.025463464 | 2.9617636 | up | noncoding | BE628551 |
| A_55_P2805912 | 0.028727155 | 2.9023087 | up | noncoding | NR_024068 |
| CUST_OEV3_044050 | 0.020733913 | 2.0232897 | down | noncoding | NONMMUT082476.1 |
| CUST_OEV3_044911 | 2.76E-04 | 5.9115076 | up | noncoding | NONMMUT081366.1 |
| CUST_OEV3_045207 | 0.011185037 | 2.1614308 | down | noncoding | NONMMUT080953.1 |
| CUST_OEV3_023093 | 0.022570238 | 2.3256834 | down | noncoding | NONMMUT109867.1 |
| CUST_OEV3_015803 | 0.04120048 | 3.1141272 | down | noncoding | NONMMUT119821.1 |
| CUST_OEV3_027233 | 0.016304808 | 2.0847702 | down | noncoding | NONMMUT104318.1 |
| CUST_OEV3_010523 | 0.038651627 | 4.570794 | up | noncoding | NONMMUT126820.1 |
| CUST_OEV3_060309 | 0.041735586 | 2.1898491 | up | noncoding | NONMMUT043297.2 |
| CUST_OEV3_005665 | 0.004887526 | 2.8649147 | down | noncoding | AK034317 |
| CUST_OEV3_062366 | 0.006176388 | 2.8046105 | up | noncoding | NONMMUT037210.2 |
| CUST_OEV3_034971 | 0.01701438 | 2.5719278 | up | noncoding | NONMMUT094379.1 |
| CUST_OEV3_074267 | 0.03895666 | 3.5238767 | up | noncoding | NONMMUT002170.2 |
| CUST_OEV3_057509 | 0.033588916 | 2.2062414 | down | noncoding | AK133710 |
| CUST_OEV3_011132 | 0.024795003 | 2.1407707 | up | noncoding | NONMMUT125983.1 |

| CUST_OEV3_000172 | 0.046921194 | 2.58991 | down | noncoding | ENSMUST00000124904 |
| --- | --- | --- | --- | --- | --- |
| A_55_P1969800 | 0.025287997 | 3.172171 | up | noncoding | XR_863788 |
| CUST_OEV3_000165 | 0.039706644 | 2.0385644 | up | noncoding | NovelTID_00006661 |
| CUST_OEV3_050587 | 0.022429913 | 2.143454 | up | noncoding | NAP061102-1 |
| CUST_OEV3_059459 | 0.031975754 | 2.417113 | up | noncoding | NONMMUT046033.2 |
| CUST_OEV3_013744 | 0.01765646 | 2.0117466 | up | noncoding | NONMMUT122569.1 |
| CUST_OEV3_021766 | 0.001175912 | 2.2250555 | down | noncoding | NONMMUT111726.1 |
| CUST_OEV3_048386 | 0.04196495 | 4.3552084 | up | noncoding | NONMMUT076852.1 |
| CUST_OEV3_089835 | 0.005392525 | 2.1996896 | up | noncoding | AK138454 |
| CUST_OEV3_090054 | 0.03767164 | 3.0541022 | up | noncoding | AK076807 |
| CUST_OEV3_025812 | 0.007344774 | 2.3537662 | down | noncoding | NONMMUT106199.1 |
| CUST_OEV3_061890 | 0.018400652 | 3.090474 | down | noncoding | AK138771 |
| CUST_OEV3_069184 | 0.018987538 | 2.2382882 | down | noncoding | AK015450 |
| CUST_OEV3_047429 | 0.04481742 | 5.3982697 | down | noncoding | NONMMUT078111.1 |
| CUST_OEV3_074787 | 0.002728142 | 2.1944866 | up | noncoding | AK054094 |
| CUST_OEV3_012155 | 0.045514192 | 2.2626386 | up | noncoding | NONMMUT124622.1 |
| CUST_OEV3_028008 | 0.001494844 | 2.596015 | up | noncoding | NONMMUT103351.1 |
| CUST_OEV3_074300 | 0.046257634 | 2.5979424 | up | noncoding | NONMMUT002040.2 |
| CUST_OEV3_033007 | 0.046353124 | 2.5685232 | down | noncoding | NONMMUT096868.1 |
| CUST_OEV3_042382 | 0.022464223 | 2.6801684 | down | noncoding | NONMMUT084618.1 |
| CUST_OEV3_072695 | 0.02456017 | 2.0032814 | down | noncoding | AK079185 |
| A_55_P2317580 | 0.01908832 | 2.2418275 | up | noncoding | NR_045902 |
| CUST_OEV3_008783 | 0.003060066 | 5.014889 | up | noncoding | NONMMUT129165.1 |
| CUST_OEV3_017058 | 0.002366658 | 2.352144 | up | noncoding | NONMMUT118075.1 |
| CUST_OEV3_065971 | 0.006479818 | 2.0791523 | down | noncoding | AK021303 |
| CUST_OEV3_070554 | 0.02839749 | 5.2723827 | up | noncoding | NONMMUT013527.2 |
| CUST_OEV3_046846 | 0.01179789 | 2.322178 | up | noncoding | NONMMUT078789.1 |
| CUST_OEV3_042136 | 0.036392707 | 2.1317906 | up | noncoding | NONMMUT084944.1 |
| CUST_OEV3_017012 | 0.005182943 | 2.0557826 | down | noncoding | NONMMUT118136.1 |
| CUST_OEV3_027185 | 0.003789353 | 2.2182255 | down | noncoding | NONMMUT104380.1 |
| CUST_OEV3_021091 | 0.020868406 | 2.0629356 | up | noncoding | NONMMUT112666.1 |
| CUST_OEV3_093272 | 0.006731256 | 2.2498665 | up | noncoding | ENSMUST00000158631.1 |
| CUST_OEV3_030906 | 0.049638513 | 2.0478373 | up | noncoding | NONMMUT099521.1 |
| CUST_OEV3_074056 | 0.025470156 | 3.3459666 | up | noncoding | AK016925 |
| A_55_P2508293 | 0.023361132 | 2.0938857 | up | noncoding | NR_045199 |
| CUST_OEV3_038934 | 0.04739676 | 2.0206802 | up | noncoding | NONMMUT089164.1 |
| CUST_OEV3_032446 | 0.00701218 | 2.2773395 | up | noncoding | NONMMUT097586.1 |
| CUST_OEV3_065550 | 0.032735698 | 2.3170977 | up | noncoding | AK017399 |
| CUST_OEV3_044147 | 7.21E-04 | 7.541498 | up | noncoding | XR_380660 |
| CUST_OEV3_063603 | 0.015427688 | 2.4734082 | up | noncoding | AK142309 |
| CUST_OEV3_069121 | 0.043597132 | 2.4138916 | up | noncoding | NONMMUT017523.2 |
| CUST_OEV3_009988 | 0.002513806 | 3.517615 | up | noncoding | NONMMUT127556.1 |
| A_66_P101763 | 0.0319144 | 3.051754 | up | noncoding | XR_373237 |
| CUST_OEV3_052260 | 0.035436213 | 2.3394752 | down | noncoding | AK141258 |
| CUST_OEV3_064675 | 0.025084605 | 4.8103056 | up | noncoding | AK081053 |
| CUST_OEV3_032894 | 0.049637277 | 2.2488353 | up | noncoding | NONMMUT097017.1 |
| CUST_OEV3_045765 | 0.02275566 | 2.1811395 | down | noncoding | NONMMUT080211.1 |
| CUST_OEV3_020601 | 0.006753841 | 4.7406635 | up | noncoding | NONMMUT113346.1 |
| CUST_OEV3_046299 | 0.019891925 | 3.4958136 | up | noncoding | NONMMUT079516.1 |
| CUST_OEV3_073419 | 0.007013598 | 2.413101 | down | noncoding | NONMMUT004882.2 |
| CUST_OEV3_035354 | 0.011578474 | 2.0138235 | down | noncoding | NONMMUT093896.1 |
| CUST_OEV3_019010 | 0.015880454 | 2.0144753 | down | noncoding | NONMMUT115495.1 |
| CUST_OEV3_038641 | 0.00584533 | 4.4266725 | up | noncoding | NONMMUT089556.1 |
| CUST_OEV3_068301 | 0.02155887 | 2.354549 | up | noncoding | NONMMUT020121.2 |
| CUST_OEV3_006369 | 0.013034095 | 2.0110114 | up | noncoding | NONMMUT132153.1 |
| CUST_OEV3_005553 | 0.00245133 | 6.662636 | up | noncoding | NONMMUT133193.1 |
| CUST_OEV3_011795 | 0.03948097 | 2.879107 | down | noncoding | NONMMUT125150.1 |
| CUST_OEV3_016307 | 0.020905651 | 3.907063 | up | noncoding | NONMMUT119077.1 |
| CUST_OEV3_028437 | 0.02118779 | 2.3394132 | down | noncoding | NONMMUT102816.1 |
| CUST_OEV3_091300 | 0.041609313 | 2.6416228 | up | noncoding | AK089387 |
| CUST_OEV3_063500 | 0.00191267 | 6.0390644 | up | noncoding | XR_386574 |
| CUST_OEV3_010240 | 0.001040414 | 3.3038802 | down | noncoding | NONMMUT127220.1 |
| CUST_OEV3_015938 | 0.01019288 | 2.0070684 | up | noncoding | NONMMUT119641.1 |

| CUST_OEV3_026344 | 0.006331648 | 2.1403754 | up | noncoding | NONMMUT105490.1 |
| --- | --- | --- | --- | --- | --- |
| CUST_OEV3_003026 | 0.048199765 | 2.0914543 | down | noncoding | NONMMUT136506.1 |
| CUST_OEV3_068347 | 0.041954283 | 2.2757578 | up | noncoding | NONMMUT019991.2 |
| CUST_OEV3_006908 | 0.035171937 | 2.4724717 | up | noncoding | NONMMUT131425.1 |
| CUST_OEV3_039004 | 0.006490108 | 2.926408 | up | noncoding | NONMMUT089062.1 |
| CUST_OEV3_059209 | 0.027612379 | 2.1368678 | down | noncoding | AK047550 |
| CUST_OEV3_066757 | 0.019434165 | 2.1465194 | up | noncoding | XR_384329 |
| CUST_OEV3_029670 | 0.032340683 | 2.0290477 | up | noncoding | NONMMUT101145.1 |
| CUST_OEV3_051709 | 0.002996444 | 2.4534967 | up | noncoding | NONMMUT069266.2 |
| CUST_OEV3_057793 | 0.006537617 | 2.954284 | up | noncoding | NR_030671 |
| CUST_OEV3_021518 | 0.026198523 | 2.5815601 | up | noncoding | NONMMUT112059.1 |
| CUST_OEV3_063459 | 0.003982295 | 3.24264 | up | noncoding | AK005117 |
| CUST_OEV3_032151 | 0.001132931 | 2.113352 | up | noncoding | NONMMUT097962.1 |
| CUST_OEV3_052026 | 0.04239633 | 3.4314246 | up | noncoding | AK139724 |
| CUST_OEV3_053358 | 0.025332494 | 3.2190368 | down | noncoding | AK132510 |
| CUST_OEV3_071504 | 0.016679158 | 2.0615532 | down | noncoding | AK013574 |
| CUST_OEV3_064084 | 0.001728211 | 5.515767 | up | noncoding | AK037518 |
| CUST_OEV3_004094 | 0.011789418 | 2.1997783 | up | noncoding | NONMMUT135088.1 |
| CUST_OEV3_023336 | 0.047837686 | 2.1722796 | down | noncoding | NONMMUT109546.1 |
| CUST_OEV3_030954 | 0.01563605 | 9.075783 | up | noncoding | NONMMUT099453.1 |
| CUST_OEV3_059849 | 0.002202522 | 2.0766208 | up | noncoding | AK086215 |
| A_51_P498274 | 0.026748862 | 2.21023 | up | noncoding | NR_037691 |
| CUST_OEV3_005972 | 0.031622563 | 2.6766486 | up | noncoding | NONMMUT132663.1 |
| CUST_OEV3_067901 | 0.003534127 | 2.2886882 | down | noncoding | AK051167 |
| CUST_OEV3_018809 | 0.018886195 | 6.00409 | up | noncoding | NONMMUT115759.1 |
| CUST_OEV3_012928 | 0.028044952 | 2.0024467 | up | noncoding | XR_377567 |
| A_55_P1992904 | 0.029011678 | 2.0969694 | up | noncoding | XR_384917 |
| CUST_OEV3_032931 | 0.03138096 | 2.098022 | down | noncoding | NONMMUT096969.1 |
| CUST_OEV3_027455 | 0.016701775 | 2.5130172 | up | noncoding | NONMMUT104037.1 |
| CUST_OEV3_015233 | 0.009575091 | 21.79603 | up | noncoding | NONMMUT120599.1 |
| CUST_OEV3_007306 | 0.033665735 | 4.3606305 | up | noncoding | NONMMUT130909.1 |
| CUST_OEV3_042285 | 0.047887295 | 2.088036 | down | noncoding | NONMMUT084749.1 |
| CUST_OEV3_040737 | 7.31E-05 | 6.785405 | up | noncoding | NONMMUT086804.1 |
| CUST_OEV3_002463 | 0.008058387 | 2.3575268 | down | noncoding | NONMMUT137293.1 |
| CUST_OEV3_043646 | 0.016817452 | 2.338895 | up | noncoding | NONMMUT082957.1 |
| CUST_OEV3_006353 | 0.023963049 | 3.3312259 | up | noncoding | XR_379188 |
| A_55_P2343665 | 0.008888884 | 4.8108206 | up | noncoding | NR_028445 |
| CUST_OEV3_002405 | 0.010921764 | 4.5343714 | down | noncoding | NONMMUT137369.1 |
| CUST_OEV3_049044 | 0.019110618 | 4.238133 | up | noncoding | XR_398716 |
| CUST_OEV3_043818 | 0.04177909 | 2.7103791 | up | noncoding | NONMMUT082754.1 |
| CUST_OEV3_047208 | 0.007418195 | 2.0930283 | down | noncoding | NONMMUT078382.1 |
| CUST_OEV3_022647 | 0.013871505 | 2.5029783 | up | noncoding | NONMMUT110493.1 |
| CUST_OEV3_040326 | 0.002691771 | 2.6976008 | down | noncoding | NONMMUT087343.1 |
| CUST_OEV3_004645 | 0.02676817 | 2.0914404 | down | noncoding | NONMMUT134360.1 |
| CUST_OEV3_044013 | 0.040893007 | 2.0528243 | up | noncoding | NONMMUT082522.1 |
| CUST_OEV3_090345 | 8.53E-04 | 3.139649 | up | noncoding | AK140593 |
| CUST_OEV3_060840 | 0.04481863 | 2.521097 | down | noncoding | NONMMUT041532.2 |
| A_66_P102275 | 0.024596447 | 2.2619147 | down | noncoding | XR_875282 |
| CUST_OEV3_011169 | 0.010643635 | 2.586616 | up | noncoding | NONMMUT125929.1 |
| CUST_OEV3_058336 | 0.018190335 | 2.026747 | up | noncoding | AK139163 |
| CUST_OEV3_015473 | 0.045595124 | 2.2731762 | up | noncoding | NONMMUT120296.1 |
| CUST_OEV3_044864 | 0.041759394 | 2.2075784 | down | noncoding | NONMMUT081414.1 |
| CUST_OEV3_032570 | 0.039112933 | 5.7791977 | up | noncoding | NONMMUT097431.1 |
| CUST_OEV3_092461 | 0.010160279 | 2.7993894 | up | noncoding | ENSMUST00000198003.1 |
| CUST_OEV3_016977 | 0.012019315 | 3.0149639 | up | noncoding | NONMMUT118181.1 |
| CUST_OEV3_015751 | 0.03809263 | 2.0471666 | up | noncoding | NONMMUT119907.1 |
| CUST_OEV3_058943 | 0.005307579 | 3.8445477 | down | noncoding | AK045886 |
| CUST_OEV3_062628 | 0.017762309 | 5.5908937 | up | noncoding | NONMMUT036396.2 |
| CUST_OEV3_039694 | 0.001346 | 2.7328572 | up | noncoding | NONMMUT088184.1 |
| CUST_OEV3_039439 | 0.04298423 | 2.2444127 | down | noncoding | NONMMUT088509.1 |
| CUST_OEV3_009647 | 0.014513849 | 2.0515053 | up | noncoding | NONMMUT128026.1 |
| CUST_OEV3_010156 | 0.015982108 | 2.4990053 | up | noncoding | NONMMUT127327.1 |
| CUST_OEV3_016422 | 6.18E-05 | 3.1743884 | up | noncoding | NONMMUT118917.1 |

| CUST_OEV3_007431 | 0.020681279 | 6.055336 | up | noncoding | NONMMUT130767.1 |
| --- | --- | --- | --- | --- | --- |
| CUST_OEV3_049820 | 0.00126619 | 4.919535 | up | noncoding | AK020855 |
| CUST_OEV3_056567 | 0.00897227 | 3.761896 | up | noncoding | AK008905 |
| CUST_OEV3_024346 | 0.044225816 | 2.2213428 | down | noncoding | NONMMUT108159.1 |
| CUST_OEV3_024837 | 0.020941835 | 3.2974672 | up | noncoding | NONMMUT107494.1 |
| CUST_OEV3_043291 | 0.049562518 | 2.2853894 | down | noncoding | NONMMUT083416.1 |
| CUST_OEV3_018425 | 0.03449714 | 3.4235048 | down | noncoding | NONMMUT116281.1 |
| CUST_OEV3_092625 | 1.99E-04 | 3.2903013 | up | noncoding | AK029621 |
| CUST_OEV3_028026 | 0.028026456 | 2.0541 | up | noncoding | NONMMUT103327.1 |
| CUST_OEV3_041852 | 0.004589139 | 4.9893537 | up | noncoding | NONMMUT085298.1 |
| CUST_OEV3_055076 | 0.048585985 | 2.8027556 | up | noncoding | NONMMUT058705.2 |
| CUST_OEV3_003695 | 0.007338647 | 41.31269 | up | noncoding | NONMMUT135597.1 |
| CUST_OEV3_064415 | 2.45E-04 | 4.7274084 | up | noncoding | AK148417 |
| A_55_P2143000 | 0.023648256 | 2.3169484 | up | noncoding | XR_382804 |
| CUST_OEV3_050099 | 0.047325242 | 2.3393993 | down | noncoding | AK037277 |
| CUST_OEV3_038260 | 0.010060668 | 7.6437807 | down | noncoding | TC1616118 |
| CUST_OEV3_091833 | 0.003925545 | 2.428203 | up | noncoding | AK142309 |
| CUST_OEV3_054386 | 0.04676719 | 4.013306 | up | noncoding | AK153016 |
| CUST_OEV3_003314 | 0.008496597 | 3.760897 | up | noncoding | NONMMUT136107.1 |
| CUST_OEV3_040287 | 0.007123906 | 2.2410038 | up | noncoding | NONMMUT087389.1 |
| CUST_OEV3_066412 | 0.038125627 | 2.4400086 | up | noncoding | AK141490 |
| CUST_OEV3_066823 | 0.00302341 | 2.2309108 | up | noncoding | AK033475 |
| CUST_OEV3_003711 | 0.01103282 | 3.6811037 | up | noncoding | NONMMUT135579.1 |
| CUST_OEV3_066155 | 0.005667758 | 2.6087875 | up | noncoding | NONMMUT026328.2 |
| A_55_P2538469 | 0.038892623 | 2.1828623 | down | noncoding | XR_400605 |
| CUST_OEV3_038729 | 0.001454197 | 5.4410906 | up | noncoding | NONMMUT089445.1 |
| CUST_OEV3_069623 | 0.001043502 | 8.374751 | up | noncoding | BB712881 |
| CUST_OEV3_025462 | 0.038974423 | 5.0227413 | up | noncoding | NONMMUT106654.1 |
| CUST_OEV3_049453 | 0.023298455 | 4.3301215 | up | noncoding | TC1605663 |
| CUST_OEV3_091005 | 0.02384553 | 2.6920176 | up | noncoding | AK141179 |
| A_55_P2142222 | 0.010748188 | 4.9689994 | up | noncoding | NR_033450 |
| CUST_OEV3_015621 | 0.04151204 | 2.8643458 | down | noncoding | NONMMUT120107.1 |
| CUST_OEV3_042127 | 0.033478145 | 2.155694 | up | noncoding | NONMMUT084954.1 |
| CUST_OEV3_053220 | 0.010980654 | 2.4292283 | up | noncoding | AK086392 |
| CUST_OEV3_008086 | 0.036566626 | 21.916727 | up | noncoding | NONMMUT130005.1 |
| CUST_OEV3_047099 | 0.037761226 | 2.9666765 | up | noncoding | NONMMUT078518.1 |
| CUST_OEV3_043882 | 0.02825178 | 2.5448859 | down | noncoding | NONMMUT082672.1 |
| CUST_OEV3_072401 | 0.04943321 | 2.2994947 | down | noncoding | NONMMUT007737.2 |
| CUST_OEV3_038189 | 0.040856108 | 4.776708 | down | noncoding | NONMMUT090179.1 |
| CUST_OEV3_030147 | 0.02586604 | 2.0636883 | down | noncoding | NONMMUT100462.1 |
| CUST_OEV3_023816 | 0.039812207 | 2.3936949 | up | noncoding | NONMMUT108927.1 |
| CUST_OEV3_056612 | 0.044077035 | 4.5355124 | up | noncoding | NONMMUT054545.2 |
| CUST_OEV3_042879 | 0.04351577 | 3.4326484 | up | noncoding | NONMMUT083965.1 |
| CUST_OEV3_092665 | 0.005361567 | 6.5519013 | up | noncoding | ENSMUST00000191792.1 |
| CUST_OEV3_034839 | 0.002127056 | 2.1031222 | down | noncoding | NONMMUT094552.1 |
| CUST_OEV3_019737 | 0.028182462 | 3.4194033 | up | noncoding | NONMMUT114493.1 |
| CUST_OEV3_001622 | 0.028575154 | 3.2156143 | up | noncoding | NovelTID_00001943 |
| CUST_OEV3_091062 | 0.028845789 | 2.261035 | up | noncoding | AK133090 |
| CUST_OEV3_018511 | 0.014142415 | 2.601099 | up | noncoding | NONMMUT116168.1 |
| CUST_OEV3_071935 | 0.008208048 | 2.0072556 | up | noncoding | AK013657 |
| CUST_OEV3_044445 | 0.014087122 | 3.4442213 | up | noncoding | NONMMUT081946.1 |
| CUST_OEV3_029674 | 0.005156454 | 2.1745343 | up | noncoding | NONMMUT101141.1 |
| CUST_OEV3_042771 | 0.002027238 | 2.2538123 | down | noncoding | NONMMUT084101.1 |
| CUST_OEV3_004709 | 0.026990166 | 2.2010868 | up | noncoding | NONMMUT134284.1 |
| CUST_OEV3_016068 | 0.04221263 | 3.5638533 | down | noncoding | NONMMUT119428.1 |
| CUST_OEV3_056443 | 0.03821617 | 2.1164403 | up | noncoding | NONMMUT055062.2 |
| CUST_OEV3_012040 | 0.02871283 | 2.8232322 | down | noncoding | NONMMUT124801.1 |
| CUST_OEV3_038503 | 0.007446897 | 2.0632055 | up | noncoding | NONMMUT089755.1 |
| CUST_OEV3_040155 | 0.001891521 | 6.302428 | down | noncoding | NONMMUT087574.1 |
| CUST_OEV3_039016 | 0.022481553 | 3.5286431 | up | noncoding | XR_394798 |
| CUST_OEV3_090092 | 0.005390497 | 3.0716772 | up | noncoding | AK080111 |
| CUST_OEV3_043104 | 0.04137681 | 3.0513597 | down | noncoding | NONMMUT083679.1 |
| CUST_OEV3_058775 | 0.048467096 | 2.4417975 | up | noncoding | NONMMUT047877.2 |

| CUST_OEV3_015568 | 0.028992286 | 3.3391414 | up | noncoding | ENSMUST00000031308 |
| --- | --- | --- | --- | --- | --- |
| CUST_OEV3_033558 | 0.04913566 | 2.874447 | up | noncoding | NONMMUT096160.1 |
| CUST_OEV3_054505 | 0.016487565 | 4.36291 | up | noncoding | XR_404000 |
| CUST_OEV3_050321 | 0.002890193 | 2.3850756 | up | noncoding | AK018439 |
| CUST_OEV3_053281 | 0.024077728 | 2.122903 | down | noncoding | XR_404761 |
| CUST_OEV3_057303 | 0.02622879 | 2.5627193 | down | noncoding | AK042656 |
| A_51_P397117 | 0.023593236 | 2.7956896 | up | noncoding | NR_040290 |
| CUST_OEV3_069895 | 0.0174137 | 2.226407 | up | noncoding | AK141926 |
| CUST_OEV3_056148 | 0.017895034 | 5.658411 | up | noncoding | NONMMUT055895.2 |
| CUST_OEV3_061732 | 0.039597906 | 3.1503265 | up | noncoding | AK086411 |
| CUST_OEV3_064075 | 0.011233502 | 2.8933773 | up | noncoding | AK042901 |
| CUST_OEV3_024779 | 0.026988437 | 2.213539 | up | noncoding | XR_374828 |
| CUST_OEV3_012683 | 0.005896901 | 2.7385705 | up | noncoding | NONMMUT123939.1 |
| CUST_OEV3_064169 | 0.043604203 | 2.1364377 | down | noncoding | NONMMUT032074.2 |
| CUST_OEV3_010363 | 0.008314306 | 2.5316114 | up | noncoding | NONMMUT127070.1 |
| CUST_OEV3_068009 | 0.02258222 | 3.6068597 | up | noncoding | AK080290 |
| CUST_OEV3_037713 | 0.046576668 | 2.402669 | down | noncoding | NONMMUT090824.1 |
| CUST_OEV3_017899 | 0.009733838 | 15.814638 | up | noncoding | NONMMUT116996.1 |
| CUST_OEV3_010651 | 0.030480888 | 3.5836759 | up | noncoding | NONMMUT126636.1 |
| CUST_OEV3_055812 | 0.030788507 | 4.4502482 | up | noncoding | XR_377634 |
| CUST_OEV3_058978 | 0.046258662 | 2.5068655 | up | noncoding | NONMMUT047361.2 |
| CUST_OEV3_001295 | 0.026744619 | 3.6565073 | up | noncoding | XR_385744 |
| CUST_OEV3_069178 | 0.002908697 | 2.146602 | down | noncoding | NONMMUT017339.2 |
| CUST_OEV3_039673 | 0.014998333 | 4.216551 | up | noncoding | NONMMUT088214.1 |
| CUST_OEV3_053293 | 0.024044445 | 3.3214967 | up | noncoding | NONMMUT064896.2 |
| CUST_OEV3_064768 | 0.007216048 | 2.9292028 | up | noncoding | NONMMUT030344.2 |
| CUST_OEV3_058822 | 0.005982254 | 4.8505287 | up | noncoding | AK142825 |
| CUST_OEV3_071733 | 0.004909517 | 2.994275 | up | noncoding | AK045881 |
| CUST_OEV3_033681 | 0.006719768 | 3.7447886 | up | noncoding | NONMMUT095994.1 |
| CUST_OEV3_040817 | 0.047757875 | 3.4275603 | up | noncoding | NONMMUT086716.1 |
| CUST_OEV3_003809 | 0.012358032 | 2.1394677 | up | noncoding | NONMMUT135468.1 |
| CUST_OEV3_026538 | 0.026206264 | 3.97595 | up | noncoding | NONMMUT105240.1 |
| CUST_OEV3_090829 | 0.00452589 | 4.802069 | up | noncoding | AK006004 |
| CUST_OEV3_017096 | 0.011649492 | 2.3946698 | up | noncoding | NONMMUT118018.1 |
| CUST_OEV3_060941 | 0.035060957 | 3.7991097 | down | noncoding | NONMMUT041212.2 |
| CUST_OEV3_093738 | 0.01941384 | 2.690392 | down | noncoding | ENSMUST00000128846.1 |
| CUST_OEV3_072075 | 0.005981737 | 2.7477806 | down | noncoding | AK038790 |
| CUST_OEV3_038945 | 0.002149398 | 2.1280048 | up | noncoding | NONMMUT089145.1 |
| CUST_OEV3_041212 | 0.02300763 | 4.1205797 | up | noncoding | NONMMUT086195.1 |
| CUST_OEV3_045793 | 0.008336256 | 2.176148 | up | noncoding | NONMMUT080177.1 |
| CUST_OEV3_048817 | 0.029666288 | 2.0649717 | up | noncoding | NONMMUT076260.1 |
| A_55_P2508923 | 0.00561475 | 2.1346638 | up | noncoding | NR_045085 |
| CUST_OEV3_003371 | 0.006308365 | 5.681218 | up | noncoding | NONMMUT136025.1 |
