# Supplementary material for: Transcriptome Sequencing of CeRNA Network Constructing in Status Epilepticus Mice Treated by Low-Frequency Repetitive Transcranial Magnetic Stimulation
Source: J Mol Neurosci. 2023 May 3;73(4-5):316–26. doi: 10.1007/s12031-023-02108-z (PMC10200785; doi:10.1007/s12031-023-02108-z)
Supplement: Supplementary file 2 — Supplementary file2 (DOCX 88 KB) [file 12031_2023_2108_MOESM2_ESM.docx]

**Table** **S2:** **Significantly** **and** **differentially** **expressed** **mRNAs** **in** **low** **frequency** **rTMS** **and** **sham** **rTMS** **mice.**

| **ProbeName** | **P** **value** | **Fold** **Change** **(abs)** | **Regulation** | **GeneID** | **GeneSymbol** |
| --- | --- | --- | --- | --- | --- |
| A_66_P106951 | 7.00E-06 | 2.407717 | up | unknown | unknown |
| A_66_P139295 | 2.32E-05 | 3.559306 | up | 53333 | Tomm40 |
| A_55_P2111478 | 5.10E-05 | 3.377501 | up | 77782 | Polq |
| A_55_P2069087 | 1.65E-04 | 6.6614447 | up | 620672 | Vmn2r67 |
| A_55_P1956195 | 2.25E-04 | 4.8620887 | up | 100041953 | Gm10094 |
| A_55_P2177233 | 2.56E-04 | 2.9531584 | up | 67469 | Abhd5 |
| A_66_P137439 | 2.79E-04 | 3.4696481 | up | 668880 | Stard9 |
| A_55_P2184996 | 2.95E-04 | 5.698601 | up | 237958 | Sppl2c |
| A_55_P2111855 | 3.28E-04 | 9.687664 | up | 74246 | Gale |
| A_55_P2123826 | 4.32E-04 | 2.2311437 | down | 18025 | Nfe2l3 |
| A_55_P2082096 | 6.03E-04 | 6.6999826 | up | unknown | unknown |
| A_55_P2023200 | 6.18E-04 | 2.7613194 | up | 56727 | Miox |
| A_55_P2731821 | 6.23E-04 | 3.7987273 | up | 20186 | Nr1h4 |
| A_55_P2021014 | 6.53E-04 | 3.2979915 | up | 403175 | Tigd4 |
| A_51_P144134 | 6.56E-04 | 2.5520852 | up | 70840 | Slc22a16 |
| A_55_P2019547 | 6.69E-04 | 7.0716844 | up | 12788 | Cnga1 |
| A_55_P2116744 | 6.83E-04 | 5.737025 | up | 22437 | Xirp1 |
| A_51_P453406 | 8.20E-04 | 2.7116828 | up | unknown | unknown |
| A_55_P1958043 | 9.75E-04 | 2.4701421 | up | 11754 | Aoc3 |
| A_55_P1970655 | 9.98E-04 | 2.778913 | up | 277250 | Kdm3b |
| A_52_P476877 | 0.00108066 | 7.716663 | up | 71137 | Rfx4 |
| A_55_P2817334 | 0.00111351 | 5.9998627 | up | 108837 | Ibtk |
| A_55_P1982499 | 0.00119469 | 2.4893618 | up | 235379 | Gldn |
| A_55_P2768961 | 0.00139796 | 3.8749223 | up | unknown | unknown |
| A_52_P666855 | 0.00142199 | 2.1118388 | up | 16706 | Ksr1 |
| A_66_P109459 | 0.00147554 | 2.0409577 | down | 228836 | Dlgap4 |
| A_65_P14340 | 0.00147938 | 11.231072 | up | 245945 | Rbm47 |
| A_55_P2072576 | 0.00148269 | 2.7228336 | up | 93883 | Pcdhb12 |
| A_55_P2129696 | 0.00152332 | 2.3079822 | up | 55994 | Smad9 |
| A_55_P1968799 | 0.0015238 | 2.0975409 | down | 69664 | Krtap1-5 |
| A_55_P2498943 | 0.00165046 | 3.126814 | up | 80288 | Bcl9l |
| A_55_P1974303 | 0.00169442 | 6.3032055 | up | 259015 | Olfr1038-ps |
| A_55_P2071286 | 0.00174412 | 4.2261705 | up | 353499 | Tmc4 |
| A_51_P495386 | 0.00192867 | 6.194349 | up | 407790 | Ndufa4l2 |
| A_52_P474145 | 0.00207085 | 2.6505942 | down | 30962 | Slc7a9 |
| A_55_P2104835 | 0.00211108 | 2.0825162 | up | 18438 | P2rx4 |
| A_51_P376776 | 0.00220056 | 7.3724103 | up | 12323 | Camk2b |
| A_55_P2913932 | 0.00222581 | 3.1961658 | up | 72181 | Nsun4 |
| A_55_P2800330 | 0.00223573 | 5.8710475 | up | 217826 | Kcnk13 |
| A_55_P2743777 | 0.00228571 | 2.1520774 | up | 224674 | Slc37a1 |
| A_55_P2122135 | 0.00229993 | 2.88819 | up | 258801 | Olfr907 |
| A_52_P337246 | 0.00232681 | 18.508688 | up | 16392 | Isl1 |
| A_55_P1971244 | 0.00242868 | 2.915863 | up | 407790 | Ndufa4l2 |
| A_55_P2068167 | 0.00245079 | 2.2883282 | up | 68564 | Nufip2 |
| A_55_P2049473 | 0.00246024 | 2.0121562 | up | 24056 | Sh3bp5 |
| A_66_P127929 | 0.00248344 | 2.860497 | up | 102643244 | Gm16252 |
| A_55_P2631999 | 0.00249423 | 2.8480222 | up | 15186 | Hdc |
| A_55_P1959041 | 0.00260848 | 13.649063 | up | 403088 | Tcaf3 |
| A_55_P1987610 | 0.00274575 | 5.8413305 | up | 72277 | 1700030F18Rik |
| A_51_P270364 | 0.00274965 | 2.4028692 | up | 109136 | Mmaa |
| A_55_P2073099 | 0.00283077 | 2.3872018 | up | 22040 | Trex1 |
| A_51_P148744 | 0.00283827 | 3.7025282 | up | 403174 | Msantd1 |
| A_66_P139430 | 0.00293621 | 2.0450249 | up | unknown | unknown |
| A_55_P2037081 | 0.00313547 | 7.2978315 | up | 112422 | 2610305D13Rik |
| A_55_P2758828 | 0.00317272 | 5.072544 | up | 18673 | Phb |
| A_66_P123987 | 0.00318854 | 2.0490592 | down | 232974 | Gm4881 |
| A_52_P172201 | 0.00319743 | 2.9868164 | up | 231503 | Tmem150c |
| A_55_P2126695 | 0.00332242 | 5.3534317 | up | unknown | unknown |
| A_51_P181175 | 0.00335932 | 2.7212574 | up | 58522 | Trim54 |
| A_55_P2748793 | 0.00342943 | 2.2091346 | up | 269423 | 3110057O12Rik |

| A_55_P2032265 | 0.00355775 | 2.0906734 | down | 73634 | 1700125H20Rik |
| --- | --- | --- | --- | --- | --- |
| A_52_P232637 | 0.00358786 | 2.3943563 | up | 13363 | Dhh |
| A_66_P107192 | 0.00365814 | 2.3168979 | down | 71412 | Dhrs2 |
| A_51_P166099 | 0.00373715 | 2.0865943 | down | 56629 | Dnase2b |
| A_55_P2398399 | 0.00396126 | 2.9266257 | up | 56079 | Astn2 |
| A_66_P130035 | 0.00398857 | 2.223952 | down | 16617 | Klk1b24 |
| A_51_P508474 | 0.00399685 | 2.2541502 | down | 258830 | Olfr103 |
| A_55_P2739439 | 0.00401312 | 2.843054 | up | 71375 | Foxn3 |
| A_55_P2042778 | 0.00408671 | 2.0456123 | up | 242202 | Pde5a |
| A_55_P1993522 | 0.0041348 | 2.2416356 | up | 320661 | D5Ertd579e |
| A_52_P296382 | 0.00420359 | 3.4597516 | up | 13709 | Elf1 |
| A_66_P124103 | 0.00422384 | 2.6301246 | up | unknown | unknown |
| A_55_P2001023 | 0.00422436 | 2.1072845 | up | 100061 | Lrrc19 |
| A_55_P2924238 | 0.00424845 | 6.9029922 | up | 234684 | Lrrc29 |
| A_51_P307901 | 0.00428081 | 2.280221 | down | 67687 | 1700011L22Rik |
| A_55_P2727609 | 0.00428101 | 2.3434064 | up | 77018 | Col25a1 |
| A_51_P107140 | 0.00428641 | 2.0313704 | up | 53617 | Krt35 |
| A_55_P2067266 | 0.00436892 | 2.278502 | up | 102642370 | LOC102642370 |
| A_55_P2852406 | 0.00441337 | 3.3834326 | up | 266632 | Irak4 |
| A_55_P2503910 | 0.00448608 | 2.0180945 | up | 17242 | Mdk |
| A_55_P1952334 | 0.00450092 | 3.9505885 | up | 98303 | D630023F18Rik |
| A_55_P2084716 | 0.00451135 | 15.380757 | up | 65257 | Asb3 |
| A_55_P2084542 | 0.00458707 | 2.1639476 | up | 100502985 | Gm10814 |
| A_66_P123030 | 0.00465351 | 4.809684 | up | 73250 | Ceacam5 |
| A_55_P2715046 | 0.00480752 | 2.3286293 | down | 13486 | Dr1 |
| A_66_P121397 | 0.00487244 | 31.719162 | up | 380755 | Lsmem1 |
| A_51_P492591 | 0.00506408 | 4.5542665 | up | unknown | unknown |
| A_55_P1981674 | 0.00506757 | 2.5389745 | up | 108147 | Atic |
| A_66_P106292 | 0.00511383 | 2.7738376 | up | unknown | unknown |
| A_51_P221632 | 0.00514386 | 2.3008692 | up | 67326 | 1700037H04Rik |
| A_55_P2787697 | 0.0051726 | 2.5793214 | up | 12585 | Cdr2 |
| A_55_P2180949 | 0.00520964 | 2.3805523 | down | 16822 | Lcp2 |
| A_55_P2800883 | 0.00524438 | 5.606026 | up | unknown | unknown |
| A_55_P2030239 | 0.00531797 | 2.374388 | up | 330554 | Fan1 |
| A_66_P115004 | 0.00532698 | 2.2849588 | up | 216643 | Gabrp |
| A_55_P2765280 | 0.00539745 | 3.0905669 | up | 14767 | Nmur1 |
| A_55_P1963144 | 0.00549831 | 2.7877002 | up | 97287 | Mtmr14 |
| A_66_P135383 | 0.0056067 | 3.24587 | up | unknown | unknown |
| A_52_P150547 | 0.00562698 | 2.0300744 | up | 12525 | Cd8a |
| A_55_P2907097 | 0.0057459 | 2.7855155 | up | 69761 | 1600015I10Rik |
| A_55_P2117155 | 0.00584704 | 2.3446817 | up | 11814 | Apoc3 |
| A_66_P117739 | 0.00589034 | 2.0277493 | up | 21428 | Mlx |
| A_51_P291501 | 0.00590332 | 2.6204119 | up | 218630 | Ccno |
| A_51_P144632 | 0.00595181 | 2.6745148 | up | 54390 | Sit1 |
| A_55_P2495582 | 0.00596393 | 2.8702726 | up | 71932 | Ephx3 |
| A_55_P2002757 | 0.00639683 | 2.1079378 | up | 17060 | Blnk |
| A_66_P131931 | 0.00652429 | 2.4958327 | down | 20871 | Aurkc |
| A_55_P2802560 | 0.00662065 | 2.4862638 | up | 75172 | Ccdc146 |
| A_55_P1982340 | 0.00679811 | 2.3849747 | down | unknown | unknown |
| A_51_P385086 | 0.00698291 | 2.7803004 | up | 21832 | Thpo |
| A_66_P113952 | 0.00701175 | 2.8977978 | down | 380701 | Slc47a2 |
| A_55_P2002963 | 0.0070949 | 3.2795365 | up | 12721 | Coro1a |
| A_55_P2784657 | 0.00712631 | 18.573566 | up | 56525 | Zfp235 |
| A_55_P2731191 | 0.00721693 | 3.5423062 | up | 54169 | Kat6b |
| A_52_P326513 | 0.0073028 | 2.025887 | up | 16492 | Kcna4 |
| A_55_P2797255 | 0.00736782 | 3.6745152 | up | 22695 | Zfp36 |
| A_55_P1970335 | 0.00754286 | 3.0293572 | up | 240819 | Teddm1a |
| A_55_P2195077 | 0.00783984 | 2.1113868 | up | 67925 | 1700066D14Rik |
| A_55_P2741494 | 0.00784858 | 2.831863 | up | 231855 | Ap5z1 |
| A_51_P120875 | 0.00787011 | 3.5113807 | up | 259036 | Olfr713 |
| A_55_P2130555 | 0.00791095 | 4.113143 | up | 100042784 | Prdm11 |
| A_55_P2219982 | 0.0079263 | 3.0208974 | down | 15401 | Hoxa4 |
| A_55_P2062589 | 0.0079887 | 7.3218665 | up | 17773 | Mtnr1a |

| A_55_P2719724 | 0.00820592 | 2.09458 | down | 67851 | 1700021F05Rik |
| --- | --- | --- | --- | --- | --- |
| A_66_P101834 | 0.00827293 | 2.088319 | up | 329872 | Frem1 |
| A_55_P2014319 | 0.00841501 | 13.073602 | up | 278097 | Armcx6 |
| A_51_P331827 | 0.00842935 | 2.772122 | up | 103775 | Slc25a41 |
| A_55_P2732311 | 0.00851486 | 2.1146772 | up | 320631 | Abca15 |
| A_55_P2101963 | 0.00853563 | 2.2602177 | down | 69928 | Apitd1 |
| A_55_P2088755 | 0.00867199 | 4.9001007 | up | 665891 | Krtap4-1 |
| A_51_P305052 | 0.00871237 | 2.049643 | down | 243958 | Siglecg |
| A_55_P2212968 | 0.008729 | 6.142181 | up | 12503 | Cd247 |
| A_51_P299527 | 0.0088055 | 15.281648 | up | 236149 | Slc22a26 |
| A_52_P236709 | 0.00887923 | 2.225242 | up | 170574 | Sp7 |
| A_55_P1957245 | 0.00889308 | 2.341748 | up | unknown | unknown |
| A_51_P183746 | 0.00894114 | 4.1897054 | up | 20204 | Prrx2 |
| A_55_P2736400 | 0.00903031 | 2.5377178 | up | 54189 | Rabep1 |
| A_55_P2182975 | 0.0093555 | 2.5231686 | up | 231602 | P2rx2 |
| A_66_P111015 | 0.00939328 | 2.2707486 | up | 77397 | 9530003J23Rik |
| A_55_P2137586 | 0.00939557 | 3.4005792 | up | 260305 | Nphp4 |
| A_55_P2192577 | 0.00977767 | 2.1204727 | up | 69389 | H2bfm |
| A_55_P2739888 | 0.00978012 | 2.2942193 | up | unknown | unknown |
| A_55_P2742166 | 0.01018499 | 2.8516426 | up | 70527 | Stambp |
| A_52_P236448 | 0.01018977 | 2.2760046 | down | 18053 | Ngfr |
| A_55_P2732982 | 0.01020444 | 3.0867193 | up | 56741 | Igdcc4 |
| A_55_P2865314 | 0.01030478 | 11.032246 | up | 622480 | Spocd1 |
| A_66_P104309 | 0.01047018 | 2.589871 | down | 17906 | Myl2 |
| A_52_P408970 | 0.01065596 | 2.341118 | down | unknown | unknown |
| A_55_P1975732 | 0.01068497 | 3.1356237 | up | 74206 | Sipa1l3 |
| A_51_P306710 | 0.01085985 | 2.084082 | up | 60363 | Cldn15 |
| A_52_P564444 | 0.01117555 | 5.772979 | up | 328830 | A530064D06Rik |
| A_51_P323531 | 0.01129544 | 2.1813471 | up | 75538 | Fam71e1 |
| A_55_P2090194 | 0.01142694 | 2.070216 | up | unknown | unknown |
| A_55_P2792405 | 0.01180399 | 2.0744593 | up | 109901 | Cela1 |
| A_66_P105826 | 0.01186523 | 2.5348005 | up | 230577 | Pars2 |
| A_55_P2720848 | 0.01202621 | 2.7053053 | up | 69046 | Isca1 |
| A_55_P2076631 | 0.01203825 | 3.3138382 | up | 71683 | Gypc |
| A_66_P115417 | 0.01215493 | 2.8777337 | down | unknown | unknown |
| A_66_P114809 | 0.0123678 | 2.1648512 | down | 84544 | Cd96 |
| A_51_P114222 | 0.01249595 | 2.7459693 | down | 16183 | Il2 |
| A_55_P2321503 | 0.01266965 | 2.063852 | up | unknown | unknown |
| A_55_P2840505 | 0.01287467 | 2.4363163 | up | 230393 | Focad |
| A_51_P141546 | 0.01300244 | 2.6443818 | down | 18406 | Orm2 |
| A_55_P2713360 | 0.01305506 | 2.1319106 | down | 56463 | Snd1 |
| A_51_P217517 | 0.01306441 | 2.229699 | up | 27390 | Mmel1 |
| A_51_P175483 | 0.0130748 | 2.7218466 | up | 26385 | Grk6 |
| A_66_P111317 | 0.01310607 | 2.2233808 | up | 213389 | Prdm9 |
| A_55_P2846241 | 0.01310905 | 3.2179236 | up | 212547 | BC027231 |
| A_51_P445532 | 0.01318596 | 4.752856 | up | 14654 | Glra1 |
| A_55_P2407222 | 0.01339279 | 4.0060015 | up | unknown | unknown |
| A_55_P1989048 | 0.0134064 | 2.0960202 | up | 74140 | Tm9sf1 |
| A_55_P2640309 | 0.01345325 | 9.299478 | up | 55989 | Nop58 |
| A_55_P2596694 | 0.0135285 | 4.0611057 | up | 19893 | Rpgr |
| A_55_P2920191 | 0.01361863 | 2.521967 | up | 66870 | Serbp1 |
| A_55_P2025974 | 0.01362912 | 2.8772388 | up | 20174 | Ruvbl2 |
| A_66_P103142 | 0.01363813 | 2.0400615 | up | 12638 | Cftr |
| A_51_P358445 | 0.01367831 | 2.7555056 | up | 13419 | Dnase1 |
| A_52_P169982 | 0.01382521 | 2.1505508 | down | 110935 | Atp6v1b1 |
| A_55_P2129373 | 0.01410336 | 2.5785415 | up | 268958 | Capn11 |
| A_51_P489452 | 0.01414776 | 2.0862405 | up | 12583 | Cdo1 |
| A_51_P246345 | 0.01416152 | 2.201324 | down | 17898 | Myl7 |
| A_55_P2805463 | 0.01421446 | 2.4633791 | up | 234865 | Nup133 |
| A_55_P2042202 | 0.01431084 | 2.0561128 | up | 56703 | Pigo |
| A_55_P2016792 | 0.01433952 | 2.7426548 | up | 234515 | Inpp4b |
| A_55_P2019397 | 0.01434233 | 2.7738128 | up | 56462 | Mtch1 |
| A_55_P2357254 | 0.01437166 | 2.4536963 | up | 68191 | Taco1os |

| A_51_P108108 | 0.01449283 | 5.2470574 | up | 211228 | Lrrc25 |
| --- | --- | --- | --- | --- | --- |
| A_55_P1995580 | 0.01453232 | 3.1020677 | down | 17232 | Mcpt9 |
| A_55_P2740547 | 0.0146213 | 2.014201 | up | 72144 | Slc37a3 |
| A_55_P2743867 | 0.01482506 | 2.0398557 | up | 75209 | Sv2c |
| A_55_P2157770 | 0.01486374 | 2.017457 | up | 23956 | Neu2 |
| A_55_P2486714 | 0.01500709 | 7.317565 | up | 70873 | Cnbd2 |
| A_55_P2797544 | 0.01508887 | 2.0888393 | up | 70099 | Smc4 |
| A_51_P519251 | 0.0152528 | 2.9463434 | up | 56312 | Nupr1 |
| A_51_P290904 | 0.01533615 | 3.205009 | up | 242570 | Raver2 |
| A_52_P480939 | 0.01538962 | 2.4585824 | up | 238799 | Tnpo1 |
| A_55_P1982638 | 0.01549851 | 3.1903002 | down | 639910 | Gm20767 |
| A_55_P2017755 | 0.01558183 | 2.0136118 | up | 69748 | Aldh16a1 |
| A_55_P2084797 | 0.0156235 | 2.0047438 | up | 16764 | Aff3 |
| A_52_P193029 | 0.01563152 | 2.329728 | down | 97212 | Hadha |
| A_55_P2501090 | 0.01579041 | 3.2393887 | up | 243499 | Lrrtm4 |
| A_52_P637418 | 0.01590153 | 2.5913706 | up | 212198 | Wdr25 |
| A_55_P2075703 | 0.01590189 | 10.027749 | down | 380845 | Gm904 |
| A_52_P463154 | 0.01607518 | 2.1014678 | down | 258196 | Olfr309 |
| A_55_P2029289 | 0.01609362 | 2.2695558 | up | 17125 | Smad1 |
| A_51_P241995 | 0.01639839 | 5.5445385 | up | 53867 | Col5a3 |
| A_55_P2160750 | 0.01639869 | 2.2505403 | up | 77018 | Col25a1 |
| A_51_P484158 | 0.01648625 | 5.1020947 | up | 70358 | Steap1 |
| A_52_P127270 | 0.01658506 | 2.1198924 | down | 330830 | Drc7 |
| A_55_P2122898 | 0.01661969 | 2.5818145 | up | 57751 | Rnf25 |
| A_52_P580634 | 0.01665449 | 2.9315035 | up | 633285 | Rbm46 |
| A_55_P2718052 | 0.01683298 | 2.3026752 | up | 57750 | Wdr12 |
| A_55_P2002702 | 0.01684441 | 2.1094475 | up | 66942 | Ddx18 |
| A_51_P106815 | 0.01695022 | 2.7139955 | up | 16565 | Kif21b |
| A_55_P2904432 | 0.01701046 | 10.433024 | up | 71829 | Ddi1 |
| A_55_P1991500 | 0.0173495 | 2.0043702 | up | 108689 | Obfc1 |
| A_66_P102232 | 0.01777528 | 2.0886636 | down | 15361 | Hmga1 |
| A_66_P123440 | 0.0178554 | 2.0064707 | up | 66801 | Prkrip1 |
| A_55_P2067366 | 0.01792542 | 2.407175 | up | 622675 | Zfp827 |
| A_52_P85334 | 0.01815536 | 3.9798682 | up | 258742 | Olfr146 |
| A_52_P361435 | 0.01821139 | 4.6041346 | up | 241514 | Zfp804a |
| A_66_P136711 | 0.01827465 | 2.120896 | down | unknown | unknown |
| A_51_P227594 | 0.01842621 | 3.0972354 | down | 171206 | Vmn1r27 |
| A_55_P2177712 | 0.0186184 | 2.1094823 | up | 102638515 | LOC102638515 |
| A_51_P207693 | 0.01864153 | 3.1010263 | up | 232813 | Shisa7 |
| A_55_P1958133 | 0.01872565 | 2.4733708 | up | 103836 | Zfp692 |
| A_51_P302346 | 0.01882826 | 3.1723619 | up | 258854 | Olfr985 |
| A_55_P2722045 | 0.0188335 | 3.3569746 | up | 22295 | Cdh23 |
| A_66_P124550 | 0.01890872 | 2.4418564 | up | unknown | unknown |
| A_55_P2718698 | 0.0189655 | 2.5048292 | up | 107823 | Whsc1 |
| A_52_P426768 | 0.01910994 | 3.682508 | up | 56222 | Cited4 |
| A_51_P409194 | 0.0191172 | 2.7399857 | up | 244216 | Zfp771 |
| A_55_P2714204 | 0.01930397 | 4.651619 | up | 207704 | Gtpbp10 |
| A_51_P195598 | 0.01933501 | 2.5230918 | down | 258766 | Olfr259 |
| A_55_P2011922 | 0.01939504 | 2.4825633 | up | 216438 | 9-Mar |
| A_55_P2937962 | 0.01950418 | 4.042813 | up | 668310 | Cc2d2b |
| A_55_P2042958 | 0.01950845 | 2.1026843 | up | 19360 | Rad50 |
| A_66_P122383 | 0.01951832 | 6.9107804 | up | 382088 | Omt2b |
| A_55_P2743240 | 0.01957479 | 3.0491166 | down | 218030 | Pou6f2 |
| A_55_P2017998 | 0.01959339 | 4.380901 | up | 192663 | Abcg4 |
| A_55_P2063608 | 0.01979036 | 2.222595 | up | unknown | unknown |
| A_55_P2721084 | 0.01991257 | 2.1309102 | up | 12788 | Cnga1 |
| A_66_P108965 | 0.01994268 | 2.12533 | up | 100340 | Smpdl3b |
| A_55_P2141729 | 0.02000214 | 2.2280354 | up | 66618 | Snrnp27 |
| A_55_P2025796 | 0.02003502 | 4.9398613 | up | 208836 | Fanci |
| A_55_P2851873 | 0.02009085 | 2.0545633 | up | 14325 | Ftl1 |
| A_51_P233546 | 0.02012071 | 2.2562745 | up | 23920 | Insrr |
| A_55_P2822152 | 0.02016521 | 2.1941357 | up | unknown | unknown |
| A_66_P124021 | 0.02022023 | 2.3822968 | up | unknown | unknown |

| A_52_P10041 | 0.0202275 | 2.237069 | up | 11677 | Akr1b3 |
| --- | --- | --- | --- | --- | --- |
| A_55_P2099700 | 0.0203047 | 4.4161725 | up | 74764 | Klc4 |
| A_55_P1982211 | 0.02032025 | 2.0606546 | up | unknown | unknown |
| A_52_P481319 | 0.02036922 | 3.500331 | up | unknown | unknown |
| A_55_P2932651 | 0.02039065 | 2.013102 | up | 72649 | Tmem209 |
| A_52_P564413 | 0.02048923 | 5.258483 | up | 18705 | Pik3c2g |
| A_52_P462217 | 0.02059194 | 3.7751043 | up | 11350 | Abl1 |
| A_55_P2714375 | 0.02070746 | 2.2770565 | down | 71354 | Wdr31 |
| A_66_P116497 | 0.02080479 | 2.069662 | down | 14997 | H2-M9 |
| A_51_P418935 | 0.02097948 | 5.126635 | up | 415115 | Neurl2 |
| A_55_P2024327 | 0.02099275 | 2.274478 | up | 232811 | Suv420h2 |
| A_55_P2068708 | 0.02116488 | 2.6938987 | down | 622052 | Gm6280 |
| A_66_P108903 | 0.02122878 | 2.9329112 | up | unknown | unknown |
| A_55_P2120876 | 0.02138895 | 3.1941707 | up | 381812 | Cracr2a |
| A_51_P328001 | 0.02147089 | 2.5232873 | up | 20389 | Sftpc |
| A_55_P2052563 | 0.02179877 | 2.014988 | up | 15901 | Id1 |
| A_55_P1999601 | 0.02188353 | 2.0694423 | up | 100038620 | Gm10631 |
| A_66_P111186 | 0.02211321 | 3.0432217 | up | 14814 | Grin2d |
| A_55_P2363883 | 0.02226057 | 2.2848382 | up | 56036 | Ccnl2 |
| A_55_P2731456 | 0.02241947 | 2.5836635 | down | 16871 | Lhx3 |
| A_55_P2002578 | 0.02246222 | 2.0849335 | up | 13849 | Ephx1 |
| A_55_P2148519 | 0.02255726 | 4.838887 | up | 240817 | Teddm2 |
| A_55_P2744827 | 0.02263742 | 2.3447018 | down | 60505 | Il21 |
| A_52_P445944 | 0.02267182 | 10.019148 | up | 78923 | Chsy3 |
| A_55_P2042844 | 0.02283795 | 2.2463279 | up | 18094 | Nkx2-9 |
| A_51_P155723 | 0.0228521 | 4.286811 | up | 27405 | Abcg3 |
| A_55_P2717381 | 0.02310734 | 2.1428916 | down | 14613 | Gja5 |
| A_52_P294834 | 0.02319824 | 2.443626 | up | 71903 | Ces2f |
| A_55_P2916847 | 0.02320353 | 2.3160052 | down | 269016 | Sh3rf2 |
| A_66_P120023 | 0.02324927 | 3.8296003 | up | 19989 | Rpl7 |
| A_55_P1984198 | 0.02327203 | 2.4684193 | up | 14404 | Gabre |
| A_51_P279100 | 0.02327296 | 2.5652092 | up | 19224 | Ptgs1 |
| A_51_P514902 | 0.02331605 | 2.1165676 | down | 258292 | Olfr446 |
| A_52_P577748 | 0.02338488 | 13.842422 | up | 107321 | Lpxn |
| A_55_P2729343 | 0.02350715 | 2.0005336 | up | 72318 | Cyth4 |
| A_52_P318653 | 0.02377019 | 2.1110077 | down | 227929 | Cytip |
| A_55_P2803170 | 0.02412562 | 4.451368 | up | 338351 | Akap17b |
| A_55_P1969771 | 0.02413303 | 2.0617037 | up | 381833 | Prb1 |
| A_51_P375958 | 0.0241525 | 2.0362155 | up | 75622 | Spaca3 |
| A_51_P272023 | 0.02419181 | 2.7304294 | down | 66825 | Rnf186 |
| A_51_P452682 | 0.02419996 | 2.877718 | up | 75507 | Pou5f2 |
| A_55_P2166698 | 0.02436609 | 4.974133 | down | 14356 | Timm10b |
| A_52_P9777 | 0.02452783 | 2.456461 | down | 277939 | C2cd3 |
| A_66_P121636 | 0.02457114 | 2.4418802 | up | 319713 | Ablim3 |
| A_55_P2726779 | 0.02471115 | 4.8054414 | down | unknown | unknown |
| A_55_P2143693 | 0.02489282 | 2.0288072 | up | 269589 | Sytl1 |
| A_55_P2179821 | 0.02489911 | 2.0719259 | down | 435528 | Glyatl3 |
| A_55_P2317435 | 0.02494171 | 2.445944 | down | 98401 | AI594674 |
| A_55_P2022404 | 0.02495646 | 2.0425894 | up | 66467 | Gtf2h5 |
| A_52_P78714 | 0.02500485 | 9.534537 | up | 16775 | Lama4 |
| A_52_P564908 | 0.02503811 | 2.4996364 | up | 76846 | Rps9 |
| A_55_P1993728 | 0.02533074 | 2.083876 | down | 77462 | Tmem116 |
| A_51_P259631 | 0.02537814 | 2.0570378 | up | 22116 | Tsks |
| A_55_P1974063 | 0.02551279 | 5.050147 | up | 100040000 | Gm2545 |
| A_55_P2720158 | 0.0256758 | 2.25832 | up | 11878 | Arx |
| A_52_P654703 | 0.0257549 | 4.9954457 | up | 70928 | Trim69 |
| A_55_P2133835 | 0.02598308 | 2.08464 | up | 69441 | 1700023F06Rik |
| A_51_P126620 | 0.02645134 | 2.2592459 | up | 258189 | Olfr624 |
| A_55_P2088440 | 0.02648061 | 2.538283 | up | 70807 | Arrdc2 |
| A_55_P2083694 | 0.02650064 | 2.0245817 | up | 110751 | Adam33 |
| A_66_P135403 | 0.02652952 | 3.6190376 | up | 100155 | AI481877 |
| A_51_P335716 | 0.02656391 | 3.3778503 | up | 102247 | Agpat6 |
| A_66_P100146 | 0.02674431 | 2.0993052 | up | unknown | unknown |

| A_55_P2717977 | 0.0267968 | 2.2818239 | up | 55961 | Slc13a1 |
| --- | --- | --- | --- | --- | --- |
| A_55_P1954653 | 0.02688415 | 5.535202 | up | 330188 | Ccdc63 |
| A_51_P393114 | 0.02714227 | 2.4261289 | down | 258621 | Olfr344 |
| A_55_P2719619 | 0.02721803 | 2.2491906 | up | 224224 | Impg2 |
| A_55_P1999102 | 0.02721961 | 2.5304565 | up | 74116 | Pi16 |
| A_66_P137413 | 0.02729154 | 2.0828397 | up | unknown | unknown |
| A_55_P2054300 | 0.02761936 | 2.7112832 | up | 381903 | Alg8 |
| A_55_P2905311 | 0.02771438 | 2.5928228 | down | 63913 | Fam129a |
| A_51_P247542 | 0.02777788 | 2.170037 | down | 237930 | Ttll6 |
| A_65_P13809 | 0.02789448 | 2.36635 | up | 26430 | Parg |
| A_55_P2608430 | 0.0280141 | 2.1454074 | up | 26965 | Cul1 |
| A_51_P151586 | 0.02807803 | 2.1229384 | down | 14841 | Gsg2 |
| A_52_P175634 | 0.02828477 | 4.118692 | up | 333424 | A4gnt |
| A_55_P2717334 | 0.02842863 | 2.0859075 | up | 18771 | Pknox1 |
| A_66_P117836 | 0.02845886 | 2.5699055 | up | 18188 | Nrtn |
| A_51_P455155 | 0.02850585 | 2.839388 | down | 16499 | Kcnab3 |
| A_51_P358122 | 0.0285694 | 2.4300032 | down | 257932 | Olfr332 |
| A_55_P2119440 | 0.02861839 | 2.1494005 | up | 67048 | Vma21 |
| A_55_P1978676 | 0.02866786 | 3.3060927 | up | 68929 | Mospd3 |
| A_55_P2737749 | 0.02874325 | 2.7513037 | down | 57321 | Terf2ip |
| A_55_P2041350 | 0.02878065 | 2.456594 | up | 223706 | Cyp2d34 |
| A_55_P2935470 | 0.0287851 | 4.4794 | up | 19725 | Rfx2 |
| A_55_P2500722 | 0.02911738 | 7.1641617 | up | 21847 | Klf10 |
| A_55_P2714329 | 0.02915515 | 2.4137409 | up | 73430 | 1700049G17Rik |
| A_55_P2037186 | 0.02925992 | 2.4758203 | up | 231642 | Alkbh2 |
| A_52_P534235 | 0.02934675 | 2.04022 | down | 20811 | Srms |
| A_55_P2078138 | 0.02935662 | 2.8884802 | down | 21906 | Otop1 |
| A_55_P2058761 | 0.02940933 | 2.537581 | up | 14378 | G6pc2 |
| A_66_P115118 | 0.02944833 | 2.0373776 | down | 258008 | Olfr1513 |
| A_66_P107763 | 0.02945205 | 2.557869 | down | 257883 | Olfr1357 |
| A_55_P2030848 | 0.02957778 | 4.708955 | up | 71159 | 4933416I08Rik |
| A_52_P571109 | 0.0296517 | 2.9671474 | down | unknown | unknown |
| A_51_P128463 | 0.02969705 | 2.7946663 | up | 72690 | Grrp1 |
| A_55_P2040081 | 0.02973223 | 4.091515 | up | 628456 | Gm6880 |
| A_51_P248395 | 0.02973524 | 3.7143464 | down | 258178 | Olfr180 |
| A_55_P2731826 | 0.02993751 | 6.105753 | up | 75739 | Mpp7 |
| A_55_P2713405 | 0.02997795 | 2.8676705 | up | 50492 | Thop1 |
| A_55_P2935256 | 0.03011642 | 2.9155884 | up | 225182 | Rbbp8 |
| A_55_P2026761 | 0.03017734 | 2.1342695 | up | unknown | unknown |
| A_55_P1999833 | 0.0301895 | 2.831787 | up | 23963 | Tenm1 |
| A_55_P2083093 | 0.03025074 | 2.1592937 | up | 73738 | Haus7 |
| A_51_P446558 | 0.0304341 | 2.2595625 | up | 69773 | 1810026J23Rik |
| A_52_P550226 | 0.03049674 | 2.5738204 | up | 67888 | Tmem100 |
| A_51_P380279 | 0.03065949 | 2.1230807 | up | 71425 | Bpifb9a |
| A_55_P2127795 | 0.03083296 | 2.5324943 | up | 50760 | Fbxo17 |
| A_52_P44765 | 0.03084684 | 2.2524655 | up | 226849 | Ppp2r5a |
| A_52_P385489 | 0.03085308 | 2.0494635 | up | 140491 | Ppp1r3a |
| A_55_P2027831 | 0.03119532 | 2.4632318 | up | 12959 | Cryba4 |
| A_55_P2056280 | 0.03134937 | 2.2102284 | down | 72338 | Wdr89 |
| A_51_P295610 | 0.03140831 | 2.2661877 | up | 66445 | Cyc1 |
| A_66_P140947 | 0.03151342 | 3.5121677 | up | 12367 | Casp3 |
| A_51_P228936 | 0.03158328 | 3.3430417 | down | 259064 | Olfr124 |
| A_55_P2276221 | 0.03169436 | 2.2128718 | down | unknown | unknown |
| A_55_P2000289 | 0.03179802 | 2.8046267 | up | 23937 | Mab21l2 |
| A_55_P2724809 | 0.03181831 | 2.4555128 | up | 68675 | Fam172a |
| A_55_P2067246 | 0.03205807 | 2.1001065 | up | 75469 | Spata19 |
| A_55_P2154797 | 0.03206888 | 2.585688 | up | 72650 | 2810006K23Rik |
| A_55_P2737752 | 0.03220532 | 2.0095894 | up | 29875 | Iqgap1 |
| A_52_P551771 | 0.0322204 | 2.0413058 | down | 258520 | Olfr849 |
| A_55_P2001489 | 0.03263934 | 2.3349953 | down | 329244 | Il19 |
| A_55_P1968250 | 0.03274661 | 4.0953426 | up | 230459 | Cyp2j13 |
| A_55_P1994739 | 0.03283314 | 3.0904627 | down | unknown | unknown |
| A_55_P2078780 | 0.03299028 | 2.4947517 | down | 18612 | Etv4 |

| A_66_P128082 | 0.0330285 | 4.3349705 | up | unknown | unknown |
| --- | --- | --- | --- | --- | --- |
| A_55_P1957911 | 0.03305073 | 2.493044 | down | 21968 | Tom1 |
| A_55_P2879536 | 0.03305679 | 2.0169656 | up | 194952 | Jmjd4 |
| A_51_P203653 | 0.03328011 | 2.2671287 | down | 320571 | Atp8b5 |
| A_55_P2355676 | 0.03336127 | 4.5621033 | up | 71217 | 4933431I19Rik |
| A_55_P2144771 | 0.03352019 | 2.4192727 | down | 245578 | Pcdh11x |
| A_66_P109774 | 0.03362379 | 2.503079 | up | unknown | unknown |
| A_66_P119424 | 0.03363383 | 2.628627 | up | 140494 | Atp6v0a4 |
| A_66_P106070 | 0.03375661 | 2.1355186 | up | 319187 | Hist1h2bn |
| A_55_P2916433 | 0.0338611 | 2.611102 | up | 171580 | Mical1 |
| A_55_P2176917 | 0.03391008 | 2.0165904 | up | unknown | unknown |
| A_66_P111655 | 0.03405751 | 3.8368397 | up | 240960 | Dnah14 |
| A_55_P2715896 | 0.0340589 | 2.1985943 | up | 223658 | Mroh1 |
| A_55_P2806002 | 0.03419177 | 2.0153577 | up | 16164 | Il13ra1 |
| A_55_P2047043 | 0.03442524 | 2.5168276 | down | 228785 | Mylk2 |
| A_55_P2506985 | 0.03444328 | 2.3298063 | up | 16818 | Lck |
| A_52_P47598 | 0.03458584 | 11.051398 | up | 54139 | Irf6 |
| A_55_P1955821 | 0.03461198 | 3.4891174 | up | 100504346 | Gm13304 |
| A_55_P1992592 | 0.03469131 | 2.231847 | up | 16192 | Il5ra |
| A_52_P189246 | 0.03469599 | 2.7823079 | up | 17772 | Mtm1 |
| A_52_P150236 | 0.03472554 | 2.2761188 | down | 55938 | Apom |
| A_66_P107176 | 0.03475323 | 4.1459026 | up | 237256 | Zc3h12d |
| A_52_P92557 | 0.0347847 | 3.4962747 | down | 225876 | Kdm2a |
| A_55_P2003363 | 0.03507066 | 2.16141 | down | 14232 | Fkbp8 |
| A_51_P408974 | 0.03518067 | 2.0920732 | down | 258785 | Olfr1230 |
| A_55_P1966271 | 0.03523182 | 2.8254554 | down | 100503041 | Pdzd7 |
| A_55_P2139092 | 0.03527283 | 6.341942 | up | 434123 | Obox8 |
| A_55_P2012146 | 0.03534752 | 7.581716 | up | 72789 | Veph1 |
| A_55_P2803555 | 0.03555765 | 2.3139837 | up | 101095 | Zfp282 |
| A_51_P162144 | 0.03558363 | 2.8167288 | up | 18431 | Oca2 |
| A_55_P2279685 | 0.03580669 | 2.877931 | up | 100504421 | 2900076A07Rik |
| A_55_P2112637 | 0.03581265 | 5.426919 | up | 226243 | Habp2 |
| A_51_P191572 | 0.03600715 | 2.1219745 | down | 258704 | Olfr411 |
| A_55_P2182392 | 0.03602001 | 3.3627853 | up | 100113398 | Adat3 |
| A_55_P2043083 | 0.03626658 | 2.2209508 | down | 277753 | Cyp4a12a |
| A_55_P2020976 | 0.03642229 | 2.7688913 | down | 78911 | Trim42 |
| A_55_P2739132 | 0.03645537 | 2.0033622 | down | 93836 | Rnf111 |
| A_52_P517140 | 0.03649197 | 2.0648892 | up | 230796 | Wdtc1 |
| A_55_P2180091 | 0.03651331 | 2.8977232 | down | 67867 | Lrrc28 |
| A_52_P855955 | 0.03655512 | 3.2624376 | up | 94212 | Pag1 |
| A_55_P2830861 | 0.03694552 | 3.0247607 | up | 100515 | Zfp518b |
| A_55_P1972623 | 0.03707758 | 2.2353005 | up | 328643 | Vwa5b2 |
| A_66_P101550 | 0.03719008 | 2.3277097 | down | 102634429 | LOC102634429 |
| A_55_P2382105 | 0.03731269 | 2.990725 | up | 232406 | BC035044 |
| A_52_P134195 | 0.03731601 | 2.1276307 | up | 26366 | Ceacam10 |
| A_52_P732441 | 0.03789687 | 2.6183333 | up | 632687 | 10-Mar |
| A_55_P1974961 | 0.0379662 | 2.4707594 | down | 263406 | Plekhg3 |
| A_51_P290387 | 0.03798687 | 2.3421574 | up | 71578 | Sval1 |
| A_66_P135653 | 0.03800717 | 2.5627415 | down | 105242927 | Gm38999 |
| A_55_P2730028 | 0.0380297 | 2.9385448 | up | 171207 | Arhgap4 |
| A_55_P1958652 | 0.03822858 | 2.1342745 | up | 11841 | Arf2 |
| A_55_P2170405 | 0.03827373 | 3.5336459 | up | unknown | unknown |
| A_51_P267314 | 0.03840689 | 2.0695667 | up | 108995 | Tbc1d10c |
| A_51_P424130 | 0.03849919 | 2.3512998 | up | 69724 | Rnaseh2a |
| A_55_P2714539 | 0.03852508 | 2.365606 | down | 227095 | Hibch |
| A_55_P2043486 | 0.03873948 | 2.860064 | up | 17702 | Msx2 |
| A_55_P2088149 | 0.03876936 | 2.4579203 | up | unknown | unknown |
| A_55_P1966874 | 0.0388455 | 3.0357776 | down | 234542 | Rtbdn |
| A_51_P511315 | 0.03896093 | 2.612814 | up | 19200 | Pstpip1 |
| A_55_P2002975 | 0.03900851 | 2.9176435 | down | 233230 | Mrgprb4 |
| A_55_P1966239 | 0.03964028 | 3.5431926 | down | 27205 | Podxl |
| A_55_P2036615 | 0.03968674 | 2.842888 | up | 321000 | Lrif1 |
| A_51_P386688 | 0.03991725 | 2.0042517 | up | 170939 | Krtap19-9b |

| A_55_P2904595 | 0.04004739 | 5.0661397 | up | 16833 | Ldhc |
| --- | --- | --- | --- | --- | --- |
| A_55_P2028734 | 0.04034088 | 4.374251 | up | 27424 | Klra16 |
| A_55_P2184370 | 0.0404245 | 2.4181805 | up | unknown | unknown |
| A_55_P2106525 | 0.04081488 | 2.1214113 | down | 74080 | Nmnat3 |
| A_65_P15913 | 0.04121216 | 2.3116522 | down | 26364 | Adgre5 |
| A_55_P2105195 | 0.04145817 | 2.7875242 | down | 83564 | Nlrp4c |
| A_66_P106743 | 0.04151637 | 2.2847943 | up | unknown | unknown |
| A_55_P2640507 | 0.04151953 | 2.6193984 | down | 74355 | Smchd1 |
| A_55_P2055504 | 0.04152108 | 4.396158 | up | 29865 | Cabp5 |
| A_55_P2928917 | 0.04160496 | 4.346603 | up | 71885 | 2310003H01Rik |
| A_66_P106836 | 0.04163694 | 2.070812 | up | 98256 | Kmo |
| A_55_P2055727 | 0.04165122 | 2.673257 | down | 113849 | Vmn1r52 |
| A_52_P526021 | 0.04167592 | 2.2531934 | up | 76117 | Arhgap15 |
| A_55_P2076032 | 0.04168497 | 5.336294 | up | 54652 | Cacna1f |
| A_55_P2744216 | 0.04174619 | 2.4504197 | up | 214253 | Etnk2 |
| A_51_P242978 | 0.04175309 | 2.0174036 | down | 330830 | Drc7 |
| A_51_P422934 | 0.04184054 | 2.0911286 | down | 258956 | Olfr535 |
| A_51_P261560 | 0.04194162 | 2.4917035 | up | 73376 | Tex33 |
| A_52_P97595 | 0.04194197 | 2.1802745 | up | 268780 | Egflam |
| A_55_P2046463 | 0.04204704 | 2.5530043 | up | 259117 | Olfr560 |
| A_55_P2099890 | 0.04205731 | 2.480703 | down | 246103 | Atxn7 |
| A_55_P2736994 | 0.04228219 | 4.384041 | up | 269473 | Lrig2 |
| A_66_P125410 | 0.04246701 | 2.0231457 | down | 258982 | Olfr1272 |
| A_55_P2062627 | 0.04278868 | 2.158122 | up | 664968 | Tmem238 |
| A_55_P2743046 | 0.04280976 | 2.603942 | up | 15364 | Hmga2 |
| A_51_P116609 | 0.04285887 | 2.1369052 | down | 66654 | Tex12 |
| A_55_P2070686 | 0.04308945 | 2.2954595 | up | 69368 | Wdfy1 |
| A_51_P497756 | 0.04315066 | 2.1867025 | down | 258963 | Olfr539 |
| A_55_P2043657 | 0.04322718 | 2.3746061 | up | 210503 | Zfp677 |
| A_52_P72186 | 0.04326192 | 2.1530046 | up | unknown | unknown |
| A_55_P2119962 | 0.0433795 | 2.1319852 | up | 73747 | 1110034G24Rik |
| A_66_P102072 | 0.04345147 | 4.1856775 | up | 237558 | Myrfl |
| A_51_P355829 | 0.0434872 | 2.621158 | up | 15967 | Ifna4 |
| A_52_P493493 | 0.04356389 | 3.195221 | up | 16924 | Lnx1 |
| A_52_P195735 | 0.04362167 | 2.632869 | down | 258439 | Olfr1309 |
| A_55_P1977426 | 0.04363159 | 2.1871166 | down | 232790 | Oscar |
| A_66_P110104 | 0.04365906 | 2.1398666 | up | 74376 | Myo18b |
| A_55_P2483987 | 0.04366679 | 2.8913832 | down | 67575 | 4930430A15Rik |
| A_55_P1986296 | 0.04396721 | 2.366683 | up | 21346 | Tagln2 |
| A_55_P2141826 | 0.04408489 | 3.61843 | down | unknown | unknown |
| A_55_P1996911 | 0.04414959 | 3.3424318 | up | 19414 | Rasa3 |
| A_66_P128378 | 0.04435417 | 2.1932569 | down | 217695 | Zfyve1 |
| A_55_P2769938 | 0.04517301 | 3.4933903 | up | unknown | unknown |
| A_51_P207622 | 0.04540262 | 2.2670312 | down | 14264 | Fmod |
| A_55_P1966583 | 0.04558189 | 2.3730512 | down | 270757 | Bpifc |
| A_51_P135118 | 0.04559181 | 2.7295456 | down | 268905 | Krtap13-1 |
| A_55_P2732683 | 0.0457769 | 3.5487912 | up | 14397 | Gabra4 |
| A_51_P273888 | 0.04583946 | 3.6039212 | down | 56410 | Cbln3 |
| A_66_P116724 | 0.0464629 | 2.1681716 | down | 330222 | Sdk1 |
| A_55_P2718830 | 0.04647128 | 2.8691497 | up | 227099 | Pms1 |
| A_66_P130527 | 0.04648576 | 2.1287518 | up | 19120 | Prm3 |
| A_55_P2097161 | 0.04675687 | 2.069025 | down | 74482 | Ifitm7 |
| A_55_P2113964 | 0.04716394 | 4.439684 | down | 66977 | Nuf2 |
| A_55_P2717628 | 0.04748821 | 3.0547462 | down | 67991 | Nacc2 |
| A_66_P138319 | 0.04777191 | 2.4764743 | up | 93732 | Acox2 |
| A_55_P2003005 | 0.04788964 | 2.2429888 | down | 12340 | Capza1 |
| A_55_P2480883 | 0.04805974 | 2.2959738 | up | 240817 | Teddm2 |
| A_55_P2927371 | 0.0480818 | 2.2183595 | down | 245650 | Gucy2f |
| A_55_P2105321 | 0.04836515 | 2.0997396 | up | 56215 | Acin1 |
| A_55_P2487134 | 0.04840269 | 2.4177043 | up | 71735 | Lrwd1 |
| A_55_P2296641 | 0.04856692 | 2.9274886 | up | unknown | unknown |
| A_55_P2713825 | 0.04865637 | 4.9715176 | up | 50931 | Il27ra |
| A_55_P2491853 | 0.04877225 | 2.7095122 | up | 271981 | Tbck |

| A_55_P2848939 | 0.04878349 | 3.901723 | up | 258105 | Olfr1162 |
| --- | --- | --- | --- | --- | --- |
| A_55_P2043782 | 0.04883374 | 3.254014 | up | 17364 | Trpm1 |
| A_55_P2029235 | 0.04884283 | 2.258692 | down | 12231 | Btn1a1 |
| A_55_P2114959 | 0.04900271 | 2.2614603 | down | 100038862 | Btnl1 |
| A_55_P2036352 | 0.04931345 | 2.1757896 | down | 18256 | Oc90 |
| A_51_P458707 | 0.04952877 | 2.0558584 | up | 257929 | Olfr299 |
| A_66_P101441 | 0.04960865 | 2.1670582 | up | unknown | unknown |
| A_55_P2057189 | 0.04962394 | 2.0124774 | up | 105242475 | LOC105242475 |
| A_55_P2163143 | 0.04991396 | 2.8010108 | up | 12567 | Cdk4 |
