# Supplementary material for: Transcriptome Sequencing of CeRNA Network Constructing in Status Epilepticus Mice Treated by Low-Frequency Repetitive Transcranial Magnetic Stimulation
Source: J Mol Neurosci. 2023 May 3;73(4-5):316–26. doi: 10.1007/s12031-023-02108-z (PMC10200785; doi:10.1007/s12031-023-02108-z)
Supplement: Supplementary file 3 — Supplementary file3 (DOCX 55 KB) [file 12031_2023_2108_MOESM3_ESM.docx]

| **Table S3: Significantly and differentially expressed circRNAs in low frequency rTMS and sham rTMS mice.** | | | | | |
| --- | --- | --- | --- | --- | --- |
| **ProbeName** | **P value** | **Fold Change (abs)** | **Regulation** | **BioType** | **TargetID** |
| CUST_OEV3_075223 | 0.011916226 | 2.7766454 | up | circRNA | mmu_circ_0016213 |
| CUST_OEV3_075505 | 0.045878273 | 2.405776 | down | circRNA | mmu_circ_0015913 |
| CUST_OEV3_076315 | 0.012413913 | 2.8816564 | up | circRNA | mmu_circ_0015009 |
| CUST_OEV3_078357 | 0.014256814 | 7.418505 | up | circRNA | mmu_circ_0012690 |
| CUST_OEV3_078941 | 0.020426456 | 2.3008132 | down | circRNA | mmu_circ_0012035 |
| CUST_OEV3_077137 | 0.037396036 | 2.059973 | up | circRNA | mmu_circ_0014033 |
| CUST_OEV3_082899 | 0.027195312 | 3.0159116 | down | circRNA | mmu_circ_0007636 |
| CUST_OEV3_077264 | 0.013792573 | 3.7865853 | up | circRNA | mmu_circ_0013874 |
| CUST_OEV3_075574 | 0.041225787 | 2.1008162 | down | circRNA | mmu_circ_0015842 |
| CUST_OEV3_080921 | 0.027680682 | 2.5952165 | up | circRNA | mmu_circ_0009832 |
| CUST_OEV3_078015 | 0.024114389 | 3.2088847 | down | circRNA | mmu_circ_0013060 |
| CUST_OEV3_081764 | 0.023947103 | 2.1997616 | down | circRNA | mmu_circ_0008884 |
| CUST_OEV3_085140 | 0.018858206 | 2.492509 | up | circRNA | mmu_circ_0005146 |
| CUST_OEV3_078481 | 0.005582575 | 5.581935 | up | circRNA | mmu_circ_0012559 |
| CUST_OEV3_078244 | 0.03538183 | 2.4885259 | down | circRNA | mmu_circ_0012809 |
| CUST_OEV3_075975 | 0.0206438 | 6.8861384 | up | circRNA | mmu_circ_0015397 |
| CUST_OEV3_088982 | 0.026562426 | 4.7360263 | up | circRNA | mmu_circ_0000903 |
| CUST_OEV3_083312 | 0.009874239 | 2.179657 | down | circRNA | mmu_circ_0007185 |
| CUST_OEV3_085943 | 0.0230236 | 2.7652576 | up | circRNA | mmu_circ_0004274 |
| CUST_OEV3_085088 | 0.028253429 | 2.6412873 | down | circRNA | mmu_circ_0005202 |
| CUST_OEV3_078347 | 0.021277523 | 2.1305397 | up | circRNA | mmu_circ_0012701 |
| CUST_OEV3_078116 | 0.045594804 | 2.2130473 | up | circRNA | mmu_circ_0012942 |
| CUST_OEV3_079709 | 0.031230807 | 5.0362496 | up | circRNA | mmu_circ_0011143 |
| CUST_OEV3_083696 | 0.010187009 | 2.3969343 | up | circRNA | mmu_circ_0006765 |
| CUST_OEV3_082140 | 0.012602084 | 2.645616 | up | circRNA | mmu_circ_0008473 |
| CUST_OEV3_077404 | 0.04783677 | 2.0589247 | down | circRNA | mmu_circ_0013728 |
| CUST_OEV3_076353 | 0.049191866 | 2.2894998 | up | circRNA | mmu_circ_0014969 |
| CUST_OEV3_079344 | 0.00453877 | 2.6161866 | up | circRNA | mmu_circ_0011587 |
| CUST_OEV3_081640 | 0.014042418 | 6.118217 | up | circRNA | mmu_circ_0009024 |
| CUST_OEV3_078295 | 0.04634966 | 2.172075 | down | circRNA | mmu_circ_0012756 |
| CUST_OEV3_084825 | 0.049435955 | 3.9411898 | up | circRNA | mmu_circ_0005496 |
| CUST_OEV3_078841 | 0.046688616 | 2.3442726 | down | circRNA | mmu_circ_0012150 |
| CUST_OEV3_082017 | 0.0287797 | 2.8616934 | up | circRNA | mmu_circ_0008611 |
| CUST_OEV3_078289 | 4.70E-04 | 2.2048614 | up | circRNA | mmu_circ_0012762 |
| CUST_OEV3_085441 | 0.021440873 | 3.866071 | up | circRNA | mmu_circ_0004825 |
| CUST_OEV3_083158 | 0.006687671 | 3.24422 | up | circRNA | mmu_circ_0007357 |
| CUST_OEV3_077564 | 0.001395866 | 4.0077257 | up | circRNA | mmu_circ_0013545 |
| CUST_OEV3_081749 | 0.029459666 | 3.9966724 | up | circRNA | mmu_circ_0008899 |
| CUST_OEV3_083742 | 0.0336946 | 3.4652128 | up | circRNA | mmu_circ_0006697 |
| CUST_OEV3_079714 | 0.004836645 | 2.3533392 | up | circRNA | mmu_circ_0011138 |
| CUST_OEV3_080087 | 0.03628608 | 2.091554 | up | circRNA | mmu_circ_0010729 |
| CUST_OEV3_089186 | 0.009684107 | 4.887528 | down | circRNA | mmu_circ_0000635 |
| CUST_OEV3_087071 | 0.04448394 | 2.4026833 | down | circRNA | mmu_circ_0003021 |
| CUST_OEV3_082093 | 0.007895573 | 3.2518303 | up | circRNA | mmu_circ_0008525 |
| CUST_OEV3_081037 | 0.020610798 | 2.1541388 | up | circRNA | mmu_circ_0009697 |
| CUST_OEV3_084442 | 0.032599553 | 3.9319358 | up | circRNA | mmu_circ_0005927 |
| CUST_OEV3_088313 | 0.00968512 | 2.27532 | down | circRNA | mmu_circ_0001647 |
| CUST_OEV3_080166 | 0.01671619 | 2.523653 | up | circRNA | mmu_circ_0010648 |
| CUST_OEV3_079550 | 0.031007284 | 2.7747784 | down | circRNA | mmu_circ_0011333 |
| CUST_OEV3_087644 | 0.00897599 | 2.1183789 | up | circRNA | mmu_circ_0002356 |
| CUST_OEV3_075901 | 0.020722095 | 3.164446 | up | circRNA | mmu_circ_0015488 |
| CUST_OEV3_078269 | 0.00658454 | 2.1135175 | up | circRNA | mmu_circ_0012784 |
| CUST_OEV3_080923 | 0.03342279 | 3.3359919 | down | circRNA | mmu_circ_0009830 |
| CUST_OEV3_082903 | 0.021870153 | 2.3611982 | up | circRNA | mmu_circ_0007632 |
| CUST_OEV3_089196 | 0.019808216 | 3.6990054 | down | circRNA | mmu_circ_0000622 |
| CUST_OEV3_081354 | 0.015463353 | 2.2145991 | down | circRNA | mmu_circ_0009335 |
| CUST_OEV3_086938 | 0.00567472 | 2.0398662 | up | circRNA | mmu_circ_0003163 |
| CUST_OEV3_081092 | 0.020197442 | 2.828791 | down | circRNA | mmu_circ_0009637 |
| CUST_OEV3_078776 | 0.04747405 | 2.4618847 | up | circRNA | mmu_circ_0012243 |
| CUST_OEV3_080336 | 0.019936087 | 2.6400323 | up | circRNA | mmu_circ_0010470 |
| CUST_OEV3_089099 | 0.02631377 | 3.0335088 | up | circRNA | mmu_circ_0000733 |
| CUST_OEV3_076412 | 0.04675212 | 4.082362 | up | circRNA | mmu_circ_0014890 |
| CUST_OEV3_086182 | 0.020351268 | 5.580052 | down | circRNA | mmu_circ_0004023 |
| CUST_OEV3_079825 | 0.037341364 | 2.0364919 | down | circRNA | mmu_circ_0011019 |
| CUST_OEV3_075469 | 0.001238337 | 6.9768004 | up | circRNA | mmu_circ_0015950 |
| CUST_OEV3_080882 | 0.011238304 | 5.4420075 | down | circRNA | mmu_circ_0009879 |
| CUST_OEV3_082536 | 0.002837797 | 3.8174028 | down | circRNA | mmu_circ_0008050 |
| CUST_OEV3_087597 | 0.040494017 | 2.9653003 | up | circRNA | mmu_circ_0002407 |
| CUST_OEV3_081921 | 0.020155644 | 2.806125 | up | circRNA | mmu_circ_0008716 |
| CUST_OEV3_081736 | 0.03882732 | 3.1380446 | up | circRNA | mmu_circ_0008912 |
| CUST_OEV3_083452 | 0.004568223 | 2.0153232 | up | circRNA | mmu_circ_0007038 |
| CUST_OEV3_086117 | 0.024018345 | 2.265468 | down | circRNA | mmu_circ_0004089 |
| CUST_OEV3_084180 | 0.01914274 | 4.4409413 | up | circRNA | mmu_circ_0006211 |
| CUST_OEV3_087122 | 0.036612812 | 2.7014089 | up | circRNA | mmu_circ_0002965 |
| CUST_OEV3_087323 | 0.03157274 | 2.0914772 | down | circRNA | mmu_circ_0002734 |
| CUST_OEV3_082015 | 0.003918833 | 5.691587 | up | circRNA | mmu_circ_0008613 |
| CUST_OEV3_088152 | 0.00810094 | 3.960312 | down | circRNA | mmu_circ_0001824 |
| CUST_OEV3_089416 | 0.009816395 | 3.5952742 | up | circRNA | mmu_circ_0000378 |
| CUST_OEV3_082210 | 0.004067516 | 2.2686622 | down | circRNA | mmu_circ_0008399 |
| CUST_OEV3_084398 | 0.004411934 | 2.9536874 | up | circRNA | mmu_circ_0005977 |
| CUST_OEV3_075852 | 0.028813919 | 4.2939615 | up | circRNA | mmu_circ_0015540 |
| CUST_OEV3_077189 | 0.020898566 | 2.0479326 | down | circRNA | mmu_circ_0013967 |
| CUST_OEV3_087300 | 0.028925803 | 2.6130593 | up | circRNA | mmu_circ_0002759 |
| CUST_OEV3_081612 | 0.019380802 | 2.9392986 | up | circRNA | mmu_circ_0009054 |
| CUST_OEV3_076553 | 0.03194998 | 2.0551412 | up | circRNA | mmu_circ_0014738 |
| CUST_OEV3_081048 | 0.046358768 | 4.1175323 | up | circRNA | mmu_circ_0009685 |
| CUST_OEV3_083201 | 0.002245485 | 2.1391447 | down | circRNA | mmu_circ_0007309 |
| CUST_OEV3_081202 | 0.005793046 | 2.4017782 | up | circRNA | mmu_circ_0009501 |
| CUST_OEV3_084087 | 0.013132694 | 2.4776695 | up | circRNA | mmu_circ_0006316 |
| CUST_OEV3_085381 | 0.03452297 | 2.0717664 | down | circRNA | mmu_circ_0004889 |
| CUST_OEV3_079627 | 0.028116342 | 2.1106951 | up | circRNA | mmu_circ_0011242 |
| CUST_OEV3_083130 | 0.011823913 | 3.5349653 | up | circRNA | mmu_circ_0007388 |
| CUST_OEV3_080456 | 0.04218083 | 2.3546798 | down | circRNA | mmu_circ_0010342 |
| CUST_OEV3_076248 | 0.03864357 | 2.144536 | up | circRNA | mmu_circ_0015090 |
| CUST_OEV3_084341 | 0.03786092 | 2.3741157 | down | circRNA | mmu_circ_0006043 |
| CUST_OEV3_077969 | 0.031330157 | 2.5338037 | up | circRNA | mmu_circ_0013117 |
| CUST_OEV3_087390 | 0.011381004 | 2.8487034 | down | circRNA | mmu_circ_0002664 |
| CUST_OEV3_075076 | 0.04824495 | 3.531194 | down | circRNA | mmu_circ_0016365 |
| CUST_OEV3_084321 | 0.033194732 | 2.1319094 | up | circRNA | mmu_circ_0006065 |
| CUST_OEV3_088227 | 0.023396982 | 2.7006285 | up | circRNA | mmu_circ_0001742 |
| CUST_OEV3_078477 | 0.03476783 | 7.739493 | up | circRNA | mmu_circ_0012564 |
| CUST_OEV3_077196 | 0.036701277 | 5.003159 | up | circRNA | mmu_circ_0013960 |
| CUST_OEV3_077689 | 0.009510524 | 3.1787932 | up | circRNA | mmu_circ_0013413 |
| CUST_OEV3_081140 | 0.028084803 | 2.4121242 | up | circRNA | mmu_circ_0009576 |
| CUST_OEV3_076218 | 0.04325028 | 3.7375674 | up | circRNA | mmu_circ_0015121 |
| CUST_OEV3_081588 | 0.013911017 | 6.514084 | up | circRNA | mmu_circ_0009082 |
| CUST_OEV3_078457 | 0.026699914 | 3.3906488 | up | circRNA | mmu_circ_0012584 |
| CUST_OEV3_078739 | 0.00745998 | 2.0131514 | up | circRNA | mmu_circ_0012289 |
| CUST_OEV3_080325 | 0.005720609 | 4.555698 | up | circRNA | mmu_circ_0010483 |
| CUST_OEV3_086093 | 0.049435157 | 4.5981627 | up | circRNA | mmu_circ_0004113 |
| CUST_OEV3_083724 | 0.045496307 | 2.7683368 | up | circRNA | mmu_circ_0006719 |
| CUST_OEV3_085242 | 0.032109756 | 5.566197 | up | circRNA | mmu_circ_0005038 |
| CUST_OEV3_076602 | 0.003769731 | 2.8401031 | up | circRNA | mmu_circ_0014684 |
| CUST_OEV3_081117 | 0.0381501 | 2.0242298 | up | circRNA | mmu_circ_0009601 |
| CUST_OEV3_086883 | 0.01281204 | 6.4097233 | up | circRNA | mmu_circ_0003227 |
| CUST_OEV3_083925 | 0.025960477 | 2.247593 | up | circRNA | mmu_circ_0006490 |
| CUST_OEV3_078761 | 0.03109283 | 2.6293952 | up | circRNA | mmu_circ_0012261 |
| CUST_OEV3_078885 | 0.034070667 | 2.4567523 | up | circRNA | mmu_circ_0012099 |
| CUST_OEV3_075959 | 0.029757222 | 4.3435955 | up | circRNA | mmu_circ_0015416 |
| CUST_OEV3_078455 | 0.031061567 | 3.4892478 | down | circRNA | mmu_circ_0012586 |
| CUST_OEV3_081626 | 0.018157398 | 2.8959844 | up | circRNA | mmu_circ_0009039 |
| CUST_OEV3_088456 | 0.041008197 | 3.2086084 | down | circRNA | mmu_circ_0001485 |
| CUST_OEV3_083723 | 0.046917975 | 3.001427 | up | circRNA | mmu_circ_0006720 |
| CUST_OEV3_081857 | 0.02853447 | 2.381801 | down | circRNA | mmu_circ_0008788 |
| CUST_OEV3_087198 | 0.04613606 | 2.320592 | up | circRNA | mmu_circ_0002886 |
| CUST_OEV3_084377 | 0.021241825 | 2.0201755 | up | circRNA | mmu_circ_0005999 |
| CUST_OEV3_084966 | 0.043848522 | 2.4622827 | up | circRNA | mmu_circ_0005337 |
| CUST_OEV3_076667 | 0.00102323 | 4.4725547 | up | circRNA | mmu_circ_0014604 |
| CUST_OEV3_089336 | 0.029169744 | 2.1405392 | up | circRNA | mmu_circ_0000466 |
| CUST_OEV3_079222 | 0.002477077 | 2.166965 | up | circRNA | mmu_circ_0011727 |
| CUST_OEV3_088181 | 0.008624311 | 4.1295056 | up | circRNA | mmu_circ_0001792 |
| CUST_OEV3_085504 | 0.018539088 | 3.208356 | up | circRNA | mmu_circ_0004761 |
| CUST_OEV3_077186 | 0.01793936 | 2.2236736 | up | circRNA | mmu_circ_0013970 |
| CUST_OEV3_089565 | 0.043035213 | 2.5709577 | up | circRNA | mmu_circ_0000217 |
| CUST_OEV3_084115 | 0.018294336 | 2.0450087 | down | circRNA | mmu_circ_0006285 |
| CUST_OEV3_085932 | 0.01233205 | 2.2278419 | up | circRNA | mmu_circ_0004287 |
| CUST_OEV3_082414 | 0.037539925 | 2.5841067 | down | circRNA | mmu_circ_0008186 |
| CUST_OEV3_080225 | 0.005717419 | 2.0861454 | up | circRNA | mmu_circ_0010584 |
| CUST_OEV3_078267 | 0.032077957 | 2.2983115 | up | circRNA | mmu_circ_0012786 |
| CUST_OEV3_083013 | 0.018845188 | 2.4966269 | up | circRNA | mmu_circ_0007510 |
| CUST_OEV3_079331 | 0.001336636 | 10.467694 | up | circRNA | mmu_circ_0011602 |
| CUST_OEV3_083767 | 0.00806914 | 2.7838883 | down | circRNA | mmu_circ_0006667 |
| CUST_OEV3_082790 | 0.012360903 | 4.8256726 | up | circRNA | mmu_circ_0007753 |
| CUST_OEV3_083644 | 0.024497056 | 2.340438 | down | circRNA | mmu_circ_0006827 |
| CUST_OEV3_085059 | 0.025699731 | 2.823937 | up | circRNA | mmu_circ_0005231 |
| CUST_OEV3_081770 | 0.004836491 | 2.2661996 | down | circRNA | mmu_circ_0008878 |
| CUST_OEV3_084210 | 0.04913367 | 3.1618094 | up | circRNA | mmu_circ_0006181 |
| CUST_OEV3_079816 | 0.009750338 | 2.1426961 | up | circRNA | mmu_circ_0011029 |
| CUST_OEV3_081225 | 0.033751402 | 2.9403207 | up | circRNA | mmu_circ_0009474 |
| CUST_OEV3_079999 | 0.032252867 | 4.1723433 | up | circRNA | mmu_circ_0010821 |
| CUST_OEV3_079028 | 0.049152046 | 4.430825 | up | circRNA | mmu_circ_0011937 |
| CUST_OEV3_080938 | 0.033695515 | 3.0861113 | down | circRNA | mmu_circ_0009810 |
| CUST_OEV3_078012 | 0.020577202 | 3.160875 | up | circRNA | mmu_circ_0013063 |
| CUST_OEV3_076693 | 0.004963584 | 2.947808 | up | circRNA | mmu_circ_0014574 |
| CUST_OEV3_089546 | 0.034778308 | 3.5495656 | up | circRNA | mmu_circ_0000236 |
| CUST_OEV3_082981 | 0.008882876 | 2.1237159 | up | circRNA | mmu_circ_0007546 |
| CUST_OEV3_084753 | 0.011117912 | 2.059525 | down | circRNA | mmu_circ_0005574 |
| CUST_OEV3_079850 | 0.007411595 | 2.2852829 | up | circRNA | mmu_circ_0010992 |
| CUST_OEV3_086090 | 0.035950694 | 2.1297913 | up | circRNA | mmu_circ_0004116 |
| CUST_OEV3_086670 | 0.019181322 | 2.4432237 | down | circRNA | mmu_circ_0003485 |
| CUST_OEV3_082755 | 0.006760546 | 2.1096938 | up | circRNA | mmu_circ_0007791 |
| CUST_OEV3_080950 | 0.013709752 | 2.3650222 | down | circRNA | mmu_circ_0009797 |
| CUST_OEV3_087723 | 0.019696234 | 3.847592 | up | circRNA | mmu_circ_0002273 |
| CUST_OEV3_078010 | 0.003137125 | 8.333121 | up | circRNA | mmu_circ_0013065 |
| CUST_OEV3_081077 | 0.016454643 | 2.2331889 | up | circRNA | mmu_circ_0009653 |
| CUST_OEV3_086305 | 0.005593765 | 5.1133037 | up | circRNA | mmu_circ_0003890 |
| CUST_OEV3_083633 | 0.004036869 | 2.790688 | down | circRNA | mmu_circ_0006841 |
| CUST_OEV3_087861 | 0.048068162 | 2.4636583 | up | circRNA | mmu_circ_0002132 |
| CUST_OEV3_076805 | 0.010147267 | 2.1608815 | down | circRNA | mmu_circ_0014451 |
| CUST_OEV3_076565 | 0.031749077 | 2.112135 | down | circRNA | mmu_circ_0014725 |
| CUST_OEV3_077722 | 0.034573212 | 2.281951 | down | circRNA | mmu_circ_0013376 |
| CUST_OEV3_079633 | 0.003841606 | 4.087294 | up | circRNA | mmu_circ_0011233 |
| CUST_OEV3_079929 | 0.008798416 | 2.0137568 | down | circRNA | mmu_circ_0010904 |
| CUST_OEV3_075437 | 0.022877876 | 2.7882912 | up | circRNA | mmu_circ_0015982 |
| CUST_OEV3_089226 | 0.027731495 | 2.5342882 | up | circRNA | mmu_circ_0000587 |
| CUST_OEV3_087027 | 0.001023086 | 2.2259877 | up | circRNA | mmu_circ_0003070 |
| CUST_OEV3_083459 | 0.001438287 | 2.1032023 | up | circRNA | mmu_circ_0007029 |
| CUST_OEV3_087036 | 0.004892477 | 2.1092362 | up | circRNA | mmu_circ_0003061 |
| CUST_OEV3_075680 | 0.012630295 | 2.132019 | up | circRNA | mmu_circ_0015730 |
| CUST_OEV3_084236 | 0.009225712 | 2.6112452 | up | circRNA | mmu_circ_0006155 |
| CUST_OEV3_080908 | 0.023917004 | 3.4494867 | up | circRNA | mmu_circ_0009849 |
| CUST_OEV3_080502 | 0.018063078 | 32.658432 | up | circRNA | mmu_circ_0010292 |
| CUST_OEV3_079298 | 0.028529301 | 2.2922516 | down | circRNA | mmu_circ_0011644 |
| CUST_OEV3_079869 | 0.009884933 | 3.9918697 | up | circRNA | mmu_circ_0010970 |
| CUST_OEV3_077631 | 5.44E-05 | 8.049362 | up | circRNA | mmu_circ_0013476 |
| CUST_OEV3_079219 | 0.00415642 | 2.5490048 | down | circRNA | mmu_circ_0011731 |
| CUST_OEV3_085067 | 3.24E-04 | 4.803432 | up | circRNA | mmu_circ_0005223 |
| CUST_OEV3_087658 | 0.002702773 | 10.328449 | up | circRNA | mmu_circ_0002342 |
| CUST_OEV3_080158 | 0.015991481 | 6.7893085 | up | circRNA | mmu_circ_0010656 |
| CUST_OEV3_080026 | 0.03648143 | 10.3061 | up | circRNA | mmu_circ_0010792 |
| CUST_OEV3_083421 | 0.028833203 | 2.413003 | down | circRNA | mmu_circ_0007070 |
| CUST_OEV3_075443 | 0.023141785 | 5.947884 | up | circRNA | mmu_circ_0015976 |
| CUST_OEV3_079855 | 0.034190375 | 2.4437478 | down | circRNA | mmu_circ_0010987 |
| CUST_OEV3_078599 | 0.012898039 | 2.6388834 | up | circRNA | mmu_circ_0012433 |
| CUST_OEV3_085786 | 0.04663424 | 2.1681292 | up | circRNA | mmu_circ_0004446 |
| CUST_OEV3_084815 | 0.039839778 | 3.3610818 | up | circRNA | mmu_circ_0005506 |
| CUST_OEV3_080784 | 0.03162463 | 2.9314294 | up | circRNA | mmu_circ_0009994 |
| CUST_OEV3_077975 | 0.044442583 | 2.532552 | up | circRNA | mmu_circ_0013110 |
| CUST_OEV3_086723 | 0.02837179 | 2.1244733 | up | circRNA | mmu_circ_0003418 |
| CUST_OEV3_083181 | 0.035841487 | 4.023392 | up | circRNA | mmu_circ_0007332 |
| CUST_OEV3_082297 | 0.023760717 | 2.137487 | up | circRNA | mmu_circ_0008308 |
| CUST_OEV3_075194 | 0.03674605 | 3.0735672 | up | circRNA | mmu_circ_0016243 |
| CUST_OEV3_082588 | 0.015507639 | 3.5024557 | up | circRNA | mmu_circ_0007976 |
| CUST_OEV3_081081 | 0.013476386 | 2.3248158 | up | circRNA | mmu_circ_0009648 |
| CUST_OEV3_078223 | 0.011729271 | 4.4615946 | up | circRNA | mmu_circ_0012830 |
| CUST_OEV3_088297 | 0.010218432 | 2.5619073 | down | circRNA | mmu_circ_0001666 |
| CUST_OEV3_082038 | 0.04917081 | 2.0982041 | up | circRNA | mmu_circ_0008588 |
| CUST_OEV3_077251 | 0.040077705 | 2.0214283 | down | circRNA | mmu_circ_0013898 |
| CUST_OEV3_080058 | 0.011484025 | 3.4135168 | up | circRNA | mmu_circ_0010759 |
| CUST_OEV3_086418 | 1.78E-04 | 2.0780044 | down | circRNA | mmu_circ_0003764 |
| CUST_OEV3_079139 | 0.046374183 | 2.3106668 | up | circRNA | mmu_circ_0011816 |
| CUST_OEV3_083711 | 0.013660309 | 2.5053327 | down | circRNA | mmu_circ_0006739 |
| CUST_OEV3_084895 | 0.02973732 | 3.9542797 | up | circRNA | mmu_circ_0005422 |
| CUST_OEV3_084071 | 0.027363729 | 2.3118858 | up | circRNA | mmu_circ_0006333 |
| CUST_OEV3_088155 | 0.029674795 | 4.672563 | up | circRNA | mmu_circ_0001821 |
| CUST_OEV3_087662 | 0.011353142 | 3.3289723 | up | circRNA | mmu_circ_0002338 |
| CUST_OEV3_077253 | 0.027435316 | 3.2572522 | up | circRNA | mmu_circ_0013896 |
| CUST_OEV3_087126 | 0.01041752 | 3.8360295 | down | circRNA | mmu_circ_0002961 |
| CUST_OEV3_087266 | 0.04678435 | 2.3009772 | up | circRNA | mmu_circ_0002802 |
| CUST_OEV3_081330 | 0.008054824 | 17.951895 | up | circRNA | mmu_circ_0009360 |
| CUST_OEV3_082211 | 0.012578756 | 2.28194 | down | circRNA | mmu_circ_0008398 |
| CUST_OEV3_088936 | 0.039697275 | 2.227888 | up | circRNA | mmu_circ_0000955 |
| CUST_OEV3_089463 | 0.009354603 | 5.9731827 | up | circRNA | mmu_circ_0000326 |
| CUST_OEV3_086783 | 0.046356726 | 2.0149753 | up | circRNA | mmu_circ_0003352 |
| CUST_OEV3_080396 | 0.033147093 | 6.0351157 | up | circRNA | mmu_circ_0010405 |
| CUST_OEV3_083416 | 0.028902669 | 2.011514 | down | circRNA | mmu_circ_0007075 |
| CUST_OEV3_080697 | 0.013093606 | 2.3848166 | up | circRNA | mmu_circ_0010086 |
| CUST_OEV3_075022 | 0.018364366 | 2.6819558 | up | circRNA | mmu_circ_0016428 |
| CUST_OEV3_084104 | 0.020538377 | 7.04845 | up | circRNA | mmu_circ_0006297 |
| CUST_OEV3_081970 | 0.04383145 | 3.6972868 | up | circRNA | mmu_circ_0008661 |
| CUST_OEV3_081758 | 0.049336363 | 2.0314271 | up | circRNA | mmu_circ_0008890 |
| CUST_OEV3_077394 | 0.017423369 | 2.501653 | up | circRNA | mmu_circ_0013738 |
| CUST_OEV3_077407 | 0.01613635 | 2.1780071 | up | circRNA | mmu_circ_0013725 |
| CUST_OEV3_088774 | 0.02472867 | 3.830836 | up | circRNA | mmu_circ_0001131 |
| CUST_OEV3_085060 | 0.020961506 | 13.054667 | up | circRNA | mmu_circ_0005230 |
| CUST_OEV3_084522 | 0.029481541 | 2.3815584 | up | circRNA | mmu_circ_0005821 |
| CUST_OEV3_089619 | 0.0331043 | 2.3580863 | up | circRNA | mmu_circ_0000154 |
| CUST_OEV3_085068 | 0.018951498 | 3.1008828 | up | circRNA | mmu_circ_0005222 |
| CUST_OEV3_079254 | 0.027941894 | 2.321253 | up | circRNA | mmu_circ_0011693 |
| CUST_OEV3_089215 | 0.02172764 | 2.393701 | up | circRNA | mmu_circ_0000599 |
| CUST_OEV3_077011 | 0.005068177 | 5.5099745 | up | circRNA | mmu_circ_0014229 |
| CUST_OEV3_081064 | 0.008420176 | 2.4012845 | up | circRNA | mmu_circ_0009667 |
| CUST_OEV3_079943 | 0.01239996 | 6.2048626 | up | circRNA | mmu_circ_0010885 |
| CUST_OEV3_075067 | 0.03474294 | 3.5157316 | up | circRNA | mmu_circ_0016375 |
| CUST_OEV3_086320 | 0.004503602 | 5.5336347 | up | circRNA | mmu_circ_0003873 |
| CUST_OEV3_081861 | 0.031042973 | 2.585812 | down | circRNA | mmu_circ_0008783 |
| CUST_OEV3_080395 | 0.023363732 | 2.4094362 | up | circRNA | mmu_circ_0010406 |
| CUST_OEV3_087937 | 0.020464571 | 2.6540446 | down | circRNA | mmu_circ_0002051 |
| CUST_OEV3_077045 | 0.013135739 | 2.5343323 | up | circRNA | mmu_circ_0014179 |
| CUST_OEV3_080861 | 0.001382667 | 2.9571474 | up | circRNA | mmu_circ_0009902 |
| CUST_OEV3_086408 | 0.032774333 | 6.3923497 | up | circRNA | mmu_circ_0003778 |
| CUST_OEV3_088163 | 0.007794273 | 2.8911676 | up | circRNA | mmu_circ_0001813 |
| CUST_OEV3_084583 | 0.008960236 | 2.262264 | up | circRNA | mmu_circ_0005753 |
| CUST_OEV3_085761 | 0.016854933 | 2.900153 | up | circRNA | mmu_circ_0004472 |
| CUST_OEV3_085646 | 0.042563558 | 2.0297937 | up | circRNA | mmu_circ_0004598 |
| CUST_OEV3_087437 | 0.042169735 | 2.3428953 | up | circRNA | mmu_circ_0002605 |
| CUST_OEV3_082226 | 0.006682358 | 2.0477877 | down | circRNA | mmu_circ_0008383 |
| CUST_OEV3_078403 | 0.039240234 | 6.3284593 | up | circRNA | mmu_circ_0012643 |
| CUST_OEV3_086541 | 0.005270343 | 4.270502 | up | circRNA | mmu_circ_0003631 |
| CUST_OEV3_083720 | 0.005905259 | 2.42746 | up | circRNA | mmu_circ_0006724 |
| CUST_OEV3_081262 | 0.028052961 | 2.369493 | up | circRNA | mmu_circ_0009432 |
| CUST_OEV3_079695 | 0.021190166 | 5.0790734 | up | circRNA | mmu_circ_0011159 |
| CUST_OEV3_077765 | 0.025104124 | 3.413397 | up | circRNA | mmu_circ_0013329 |
| CUST_OEV3_079171 | 0.01242084 | 2.3969693 | up | circRNA | mmu_circ_0011781 |
| CUST_OEV3_075990 | 0.012918987 | 5.1894813 | up | circRNA | mmu_circ_0015380 |
| CUST_OEV3_081996 | 0.04326192 | 2.2721157 | down | circRNA | mmu_circ_0008633 |
| CUST_OEV3_085146 | 0.028008582 | 2.9289157 | up | circRNA | mmu_circ_0005139 |
| CUST_OEV3_082134 | 0.037941284 | 2.1344578 | up | circRNA | mmu_circ_0008479 |
| CUST_OEV3_083576 | 0.018859118 | 2.8620338 | up | circRNA | mmu_circ_0006904 |
| CUST_OEV3_083770 | 0.04332486 | 2.9342723 | up | circRNA | mmu_circ_0006664 |
| CUST_OEV3_081686 | 0.027675057 | 2.3108318 | down | circRNA | mmu_circ_0008973 |
| CUST_OEV3_075808 | 0.04826719 | 2.326499 | up | circRNA | mmu_circ_0015588 |
| CUST_OEV3_075537 | 0.001033635 | 5.4625316 | up | circRNA | mmu_circ_0015879 |
| CUST_OEV3_081533 | 0.013570256 | 2.427083 | up | circRNA | mmu_circ_0009147 |
| CUST_OEV3_087352 | 0.045587637 | 2.1509895 | up | circRNA | mmu_circ_0002704 |
| CUST_OEV3_082631 | 0.018584715 | 4.434498 | up | circRNA | mmu_circ_0007931 |
| CUST_OEV3_081321 | 0.014998357 | 6.5712743 | up | circRNA | mmu_circ_0009369 |
