# Supplementary material for: Transcriptome Sequencing of CeRNA Network Constructing in Status Epilepticus Mice Treated by Low-Frequency Repetitive Transcranial Magnetic Stimulation
Source: J Mol Neurosci. 2023 May 3;73(4-5):316–26. doi: 10.1007/s12031-023-02108-z (PMC10200785; doi:10.1007/s12031-023-02108-z)
Supplement: Supplementary file 4 — Supplementary file4 (DOCX 27 KB) [file 12031_2023_2108_MOESM4_ESM.docx]

| **Table S4: Significantly and differentially expressed miRNAs in low frequency rTMS and sham rTMS mice.** | | | | | |
| --- | --- | --- | --- | --- | --- |
| **systematic_name** | **P value** | **Fold Change (abs)** | **Regulation** | **mirbase accession No.** | **chr** |
| mmu-miR-126a-5p | 0.047000967 | 4.7278733 | up | MIMAT0000137 | chr2 |
| mmu-miR-138-2-3p | 0.001305748 | 7.06985 | up | MIMAT0016987 | chr8 |
| mmu-miR-186-5p | 1.45E-04 | 6.605041 | up | MIMAT0000215 | chr3 |
| mmu-miR-1934-3p | 0.00167549 | 5.630047 | down | MIMAT0017341 | chr11 |
| mmu-miR-19a-3p | 3.03E-04 | 5.226106 | down | MIMAT0000651 | chr14 |
| mmu-miR-28a-5p | 7.30E-04 | 7.5164533 | up | MIMAT0000653 | chr16 |
| mmu-miR-290a-3p | 0.032653846 | 3.8973336 | up | MIMAT0004572 | chr7 |
| mmu-miR-29b-1-5p | 0.001455994 | 5.789716 | up | MIMAT0004523 | chr6 |
| mmu-miR-3102-3p | 0.003777162 | 6.034577 | up | MIMAT0014936 |  |
| mmu-miR-342-5p | 4.11E-04 | 6.8185186 | up | MIMAT0004653 |  |
| mmu-miR-376a-5p | 0.001334289 | 7.269522 | up | MIMAT0003387 | chr12 |
| mmu-miR-379-3p | 7.24E-04 | 6.252736 | up | MIMAT0017080 | chr12 |
| mmu-miR-6973b-5p | 6.17E-05 | 6.1005855 | down | MIMAT0027908 |  |
| mmu-miR-6984-3p | 0.017434716 | 28.903051 | up | MIMAT0027871 |  |
| mmu-miR-7216-5p | 7.27E-04 | 6.935633 | up | MIMAT0028400 |  |
| mmu-miR-7222-3p | 4.53E-04 | 63.628048 | down | MIMAT0028413 |  |
| mmu-miR-744-5p | 0.005290075 | 4.4185333 | down | MIMAT0004187 | chr11 |
