# Supplementary figures and images for: Transcriptome Sequencing of CeRNA Network Constructing in Status Epilepticus Mice Treated by Low-Frequency Repetitive Transcranial Magnetic Stimulation
Source: J Mol Neurosci. 2023 May 3;73(4-5):316–26. doi: 10.1007/s12031-023-02108-z (PMC10200785; doi:10.1007/s12031-023-02108-z)

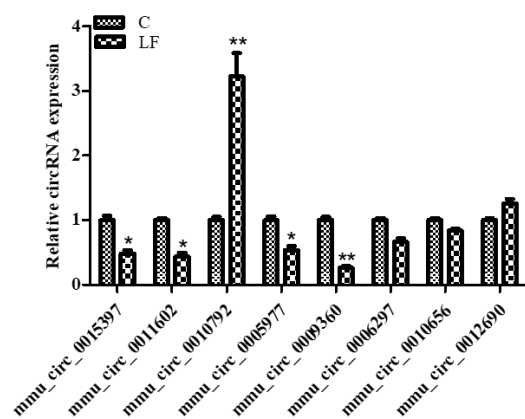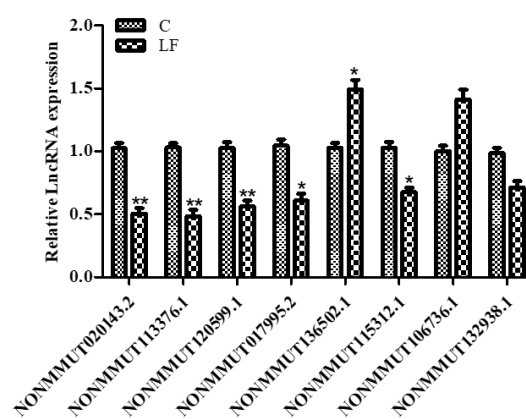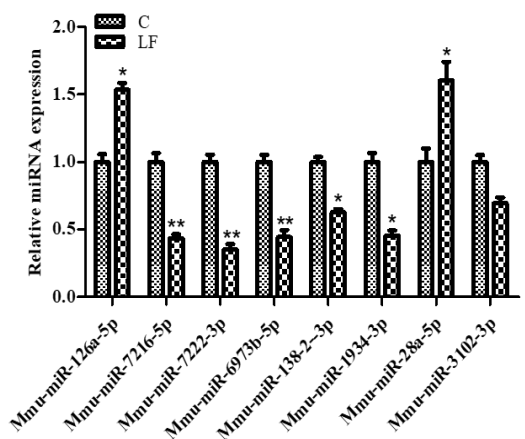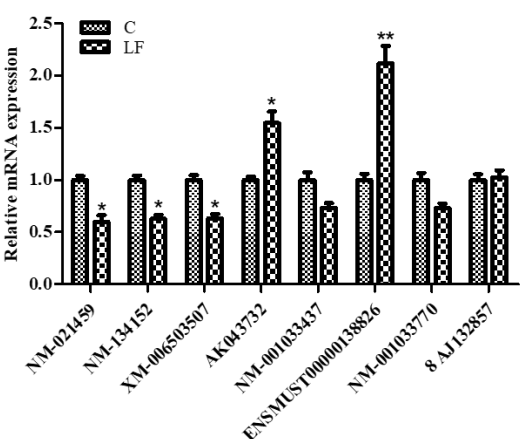

Supplement: Supplementary file 5 — Supplementary file5 (PDF 52 KB) [file 12031_2023_2108_MOESM5_ESM.pdf]
